# Label-free Raman spectroscopy for cancer therapy response: from methodological framework to clinical translation

**DOI:** 10.1039/d6cs00966b

**Published:** 2026-10-28

**Authors:** Victor Alcolea-Rodriguez, Arianna Bresci, Anna Mühlig, Chiara Agrimi, Carlo Morasso, Dario Polli, Orlando Guntinas-Lichius, Renzo Vanna

**Affiliations:** a CNR-Institute for Photonics and Nanotechnologies (CNR-IFN) P.zza Leonardo Da Vinci 32 20133 Milan Italy victor.alcolea@polimi.it renzo.vanna@cnr.it; b Department of Physics, Politecnico di Milano P.zza Leonardo da Vinci 32 20133 Milan Italy; c Laser Biomedical Research Center, G. R. Harrison Spectroscopy Laboratory, Massachusetts Institute of Technology 77 Massachusetts Avenue Cambridge, MA 02139 USA; d Clinical Biophotonics, Department of Otorhinolaryngology, Jena University Hospital 07747 Jena Germany; e Comprehensive Cancer Center Central Germany 07747 Jena Germany; f Laboratory of Nanomedicine, Istituti Clinici Scientifici Maugeri IRCCS Pavia Italy; g Department of Otorhinolaryngology, Jena University Hospital 07747 Jena Germany

## Abstract

The early detection of therapy-induced biomolecular changes and the prediction of therapeutic response remain major challenges in oncology. Current clinical methods often assess treatment response through macroscopic or morphological endpoints, whereas molecular alterations associated with sensitivity, resistance, or recurrence may arise earlier and remain undetected. Raman spectroscopy offers a label-free and non-destructive approach to probe the intrinsic biochemical composition of cells, tissues, and biofluids, providing spectral and spatial information that can support therapy response prediction and longitudinal monitoring. This review discusses Raman-based technologies for cancer therapy assessment, from preclinical models to emerging clinical applications. We first outline the clinical need for rapid, non-destructive, and molecularly informative tools, considering tumor heterogeneity, therapy resistance, the tumor microenvironment, and current standards for response evaluation. We then provide a methodological framework for selecting Raman modalities, including spontaneous Raman (SR) spectroscopy, surface-enhanced Raman spectroscopy (SERS), stimulated Raman spectroscopy (SRS), and coherent anti-Stokes Raman spectroscopy (CARS), together with key considerations on acquisition strategies, multimodal integration, experimental design, preprocessing, feature analysis, and machine learning. We summarize Raman spectral biomarkers associated with therapy-induced changes in nucleic acids, proteins, lipids, metabolites, extracellular matrix components, and anticancer drugs, and critically examine studies using Raman spectroscopy to predict or monitor responses to major therapeutic approaches. Finally, we discuss barriers to clinical translation, including acquisition speed, inter-instrument reproducibility, preclinical model limitations, regulatory requirements, workflow integration, clinical trials, and prospective validation. Overall, Raman spectroscopy emerges as a promising platform for molecular phenotyping in precision oncology, provided that future studies progress toward standardized, clinically validated, and actionable workflows.

## Abstract 2

This review critically connects Raman modalities, molecular readouts, data analysis and clinical translation for cancer therapy-response studies.

## Graphical abstract

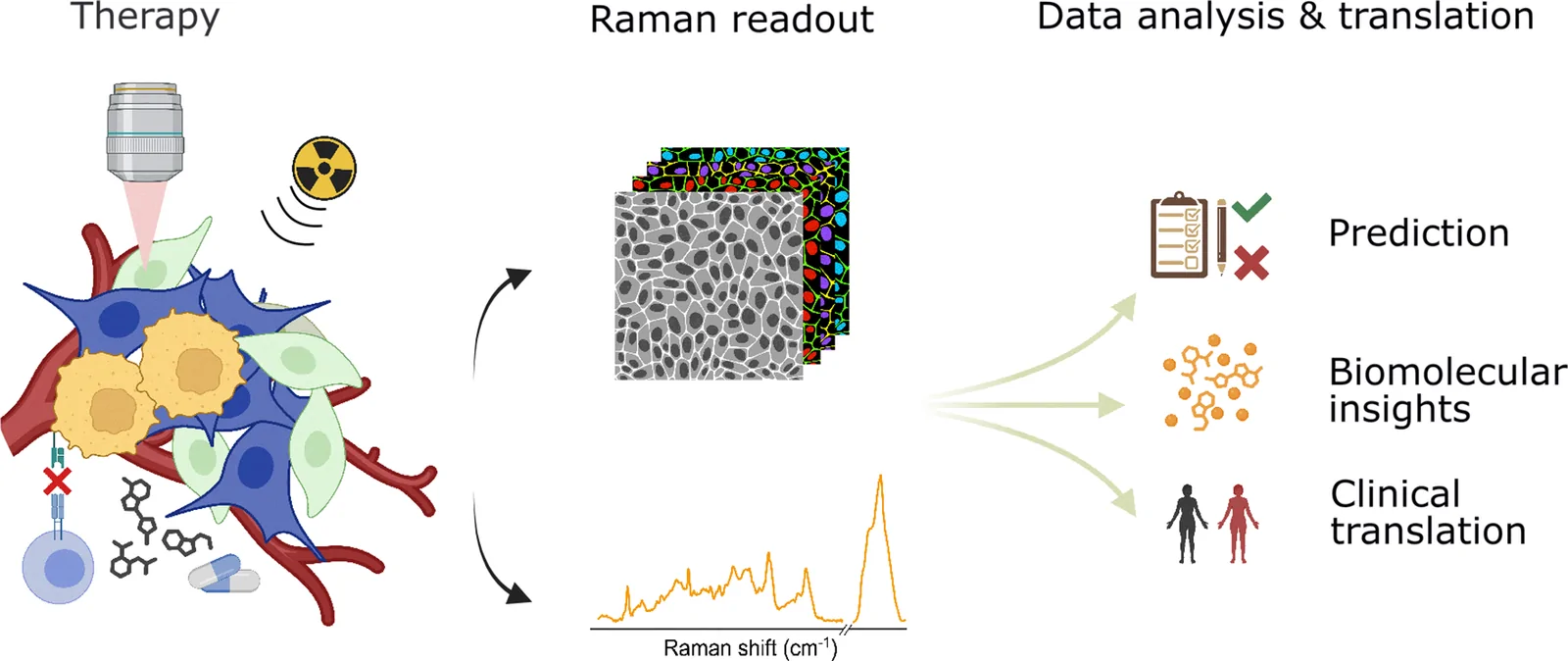

## 1 Introduction

Understanding how tumors and their surrounding microenvironment respond to anticancer therapy remains a central challenge in oncology. Capturing these dynamic processes is essential to evaluate treatment efficacy, detect resistance, and ultimately predict patient outcomes. These responses are driven by a variety of biomolecular alterations that may arise early during treatment and can occur at the genetic, transcriptional, or metabolic level, often before any morphological changes become detectable through conventional clinical methods such as imaging or histopathology.^1–3^ Raman spectroscopy, in particular, offers a label-free, non-destructive approach to assess the biochemical composition of biological samples by detecting inelastic light scattering associated with molecular vibrations.^4^ When combined with spatial information, Raman-based imaging techniques can generate high-resolution bioimages that reveal both morphological features and biomolecular characteristics within tumors and the tumor microenvironment (TME).^5^

Raman-based technologies are emerging as powerful tools for monitoring cancer therapy response, enabling real-time, longitudinal tracking of treatment-induced molecular changes. By capturing both the biochemical composition and spatial architecture of the tumor microenvironment, these approaches may allow early detection of resistance and accurate assessment of therapeutic efficacy.^6,7^ Identification of biochemical biomarkers linked to clinical outcomes further extends their potential from monitoring to prediction, enabling the anticipation of complete or partial response, stable disease, or progression. Particularly when spatially resolved Raman images are obtained, integrating biochemical and morphological features can further enhance the performance of predictive models, including those powered by artificial intelligence.^8^

Building on these capabilities, Raman spectroscopy has been applied in preclinical research to assess therapy efficacy. This includes studies using *in vitro* cell cultures,^5,9^*ex vivo* (*e.g.*, tumor xenografts or tumor biopsies),^10,11^ and more recently, in a limited number of *in vivo*^12^ experimental models. These investigations have contributed to understanding drug mechanisms of action by mapping therapy-induced metabolic and biomolecular alterations. In recent years owing to its ability to distinguish between healthy and malignant tissue, Raman spectroscopy has increasingly been integrated into clinical procedures, particularly for intraoperative guidance during surgical treatment. These Raman-based tools have been successfully applied to delineate tumor margins in real time.^13–15^ Furthermore, their clinical utility is being investigated in the context of non-invasive therapy monitoring, for example through the analysis of biofluids to assess systemic treatment response over time.

This review links the clinical need for accurate cancer therapy response prediction and monitoring with recent advances in Raman-based technologies. Table 1 defines key concepts and terminology used throughout the manuscript. Section 2 outlines cancer resistance mechanisms and current gold-standard methods in research and clinical practice. Section 3 describes label-free Raman modalities and criteria for their selection. Section 4 addresses the main Raman signatures related to spectral biomarkers of therapy-induced changes in the tumor microenvironment, while Section 5 presents strategies for experimental design and data analysis. Sections 6 and 7 summarize key findings from Raman-based studies published between 2010 and 2026, covering both prediction and monitoring, respectively. Section 8 summarizes the main Raman biomarkers reported in the literature, while Section 9 addresses challenges, perspectives, and future directions for clinical translation. Finally, Section 10 outlines the literature search protocol.

**Table 1:** Key terminology and definitions used in this literature review

Concept	Definition
Anticancer therapy	Anticancer therapy refers to treatments designed to prevent, inhibit, remove, or eliminate cancer cells. These include local treatments such as surgery and radiotherapy, as well as systemic treatments such as chemotherapy, immunotherapy, targeted therapy, and emerging cell- or gene-based approaches, which may be combined to improve patient outcomes
Therapy prediction	The process of anticipating an individual's response to medical treatment based on their biochemical profile, clinical indicators, and advanced diagnostic tools, aimed at ensuring precise and effective healthcare
Therapy response	The measurable change in a disease state or progression resulting from a therapeutic intervention. In oncology, therapy response is typically assessed using imaging techniques, biomarkers, or clinical evaluations to determine tumor regression, stability, or progression
Treatment resistance	A phenomenon where a disease, such as cancer, does not respond to a therapy that was previously effective. This resistance can be intrinsic or acquired and poses a significant challenge in disease management, often leading to treatment failure. Typically, resistance is described according to the therapy involved, *e.g.*, chemoresistance, radioresistance or immunotherapy resistance
Therapy effectiveness	The measure of how well a therapeutic intervention achieves its intended clinical outcomes, quantified by improvements in patient health status, quality of life, symptom reduction, and the retardation or prevention of disease progression. It refers to treatment achievements in real-world clinical settings
Therapy efficacy	Similar to therapy effectiveness, it measures how well a therapeutic intervention achieves its intended clinical outcomes, but under ideal and controlled conditions, such as in randomized clinical trials
Therapy efficiency	Refers to the ability of a therapy to achieve desired clinical outcomes while minimizing resource use (time, cost, effort) or risk
Therapy monitoring	The systematic and continuous assessment of an individual's (cell, tissue or patient) response to a specific treatment over time, aimed at evaluating its efficacy and detecting any adverse effects
Therapy simulation	Refers to the process of using virtual or physical models to observe, analyze, and refine the response of a treatment before it is applied to real patients. This approach allows clinicians to predict outcomes and adjust parameters before delivering the therapy
Tumor biomarker	A biological molecule found in blood, other body fluids, or tissues whose increase or decrease in concentration is a sign of a normal or abnormal process, or of a condition or disease. A biomarker may be used to see how well the body responds to a treatment
Human xenografts	A human xenograft refers to the transplantation of human cells, tissues, or organs into a different species, typically an animal model (*e.g.*, mice or rats), to replicate human biological responses and study disease mechanisms, drug efficacy, and other physiological processes in a living system

Although previous reviews have extensively discussed Raman-based approaches in oncology, their focus is generally restricted either to a single cancer type or to specific cancer therapies, such as radiotherapy,^16^ chemotherapy,^17^ surgery^15^ and immunotherapy.^6^ Additional reviews have highlighted applications for drug localization,^7^ therapy response,^18^ and resistance assessment^19^ with relevance for cancer treatment.^20^ From a clinical translation perspective, Pence *et al.* (2016) provide an overview of *in vivo* studies using Raman spectroscopy for diagnostics,^21^ while a more recent work by Cutshaw *et al.* (2023) focuses on Raman techniques for metabolic profiling and precision medicine.^22^ This review provides a comprehensive methodological and translational framework including experimental planning, Raman modality selection, and an overview of the most robust analytical pipelines to generate clinically relevant insights into the biomolecular effects of anticancer therapies and their prediction. In addition, it provides a systematic summary of the principal findings reported between 2010 and 2026, highlighting tissue-, cellular-, and model-specific biochemical responses to therapy, and identifying key Raman spectral biomarkers that have been proposed to define therapeutic response or resistance. By combining methodological guidance with an evidence-based synthesis of reported spectral biomarkers, this review aims to serve as a practical and critical framework for researchers entering or advancing the field of Raman-based cancer therapy assessment. Rather than only cataloguing existing applications, we highlight how Raman modalities, experimental design, data analysis, and clinical validation should be aligned to generate reproducible, interpretable, and clinically actionable information.

## 2 Cancer therapy response monitoring and prediction

### 2.1 Clinical demand for effective therapy response monitoring and prediction

#### 2.1.1 Tumor heterogeneity and cancer therapies

Cancer management represents a significant societal, public health, and economic challenge in the 21st century, as this group of diseases is responsible for nearly one in six deaths (16.8%) globally.^23^ Cancer arises from complex biochemical changes at the sub-cellular level, leading to gradual alterations that typically result in a heterogeneous intratumoral composition, particularly in solid tumors.^24,25^ Cancer genome diversification occurs during tumorigenesis as a dynamic evolutionary process. Randomly generated mutations give rise to the accumulation of subclonal populations within tumors, resulting in pronounced spatial heterogeneity in their genetic composition. This heterogeneity is evident not only across distinct anatomical sites (*e.g.*, primary tumors *versus* liver or brain metastases), but also between different regions within the same tumor.^26,27^ This clonal diversity complicates both diagnosis and treatment (Fig. 1).^28,29^

**Fig. 1:**
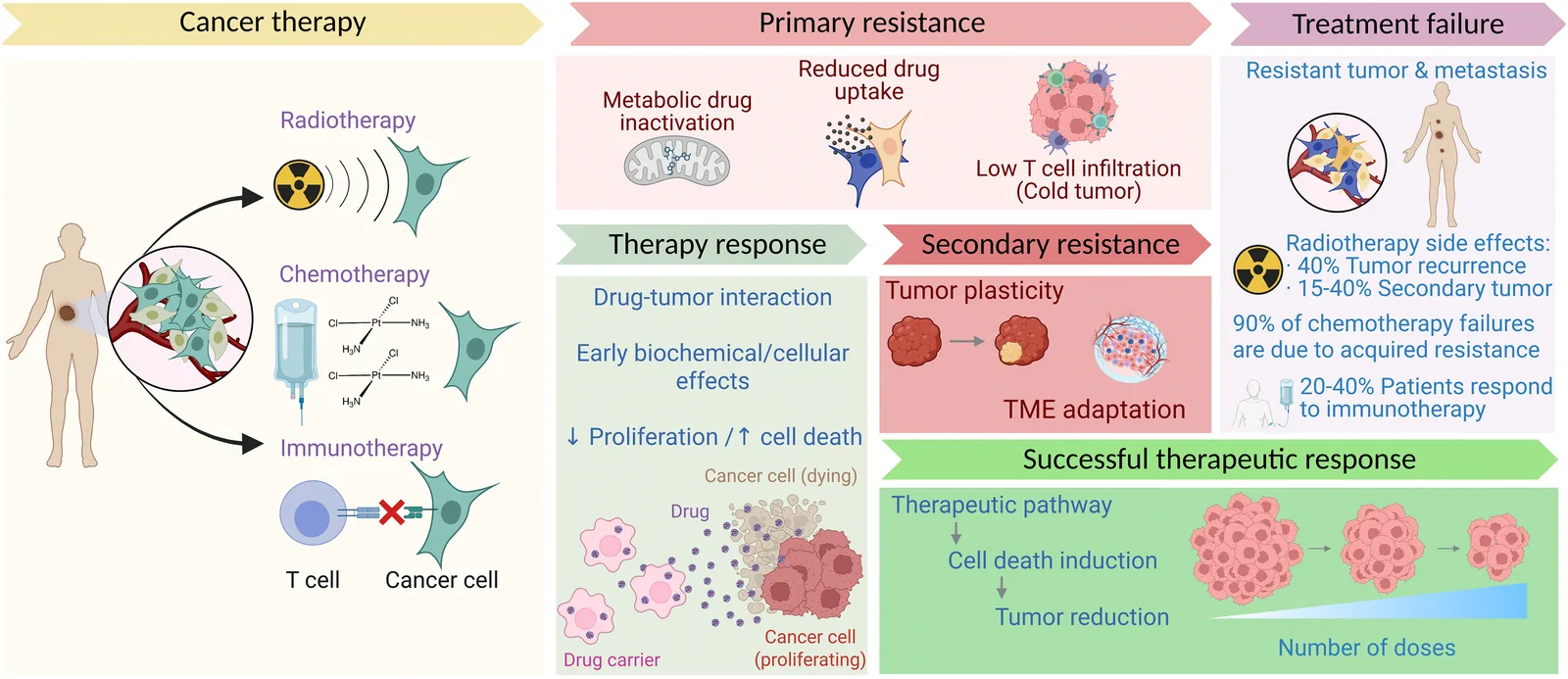
Overview of cancer therapy strategies, resistance mechanisms and tumor therapy response.

The complexity of tumors has driven substantial efforts to develop a wide range of therapeutic strategies, from established modalities such as surgery, chemotherapy, and radiotherapy to more recent advances in immunotherapies, nanoparticle-based therapies,^30,31^ small-molecule targeted agents, antibody–drug conjugates (ADCs), cell-based therapies and gene therapies.^32^ Chemotherapy remains a cornerstone of systemic cancer therapy – it uses cytotoxic drugs to target rapidly dividing cells and reduce tumor burden. By disrupting DNA replication, mitosis, and metabolic activity, these agents induce cancer cell death. Chemotherapy can be applied neoadjuvantly to shrink tumors before surgery, adjuvantly to prevent recurrence, as primary therapy for advanced cancers, or in combination with other therapies. However, its non-selective action also damages healthy proliferating cells, causing side effects.

Radiotherapy relies on the use of ionizing radiation to damage the DNA of tumor cells, ultimately leading to cell death. Radiotherapy can be used as an alternative treatment or, more frequently, in combination with chemotherapy to manage localized tumors, particularly when organ preservation is desired. The induction of apoptosis and necrosis in tumor cells is produced through the generation of reactive oxygen species (ROS) and free radicals, and possesses immunomodulatory capabilities by stimulating the production of tumor-specific antigens and boosting the initiation and activation of cytotoxic T cells.^33^

Targeted therapies and new immunotherapies are modern approaches to overcome the lack of selectivity associated with chemotherapy and radiotherapy. Targeted therapies exploit specific molecular alterations driving tumor progression - such as mutations, amplifications, or aberrant signaling pathways - to selectively inhibit cancer cell survival and proliferation. Representative examples of targeted therapies include tyrosine kinase inhibitors (TKIs), such as HER2 inhibitors, widely used in HER2-positive breast cancer, and EGFR inhibitors in the management of lung cancer.^34,35^ Immunotherapy acts by stimulating or enhancing the immune system to recognize and attack cancer cells more effectively. In this context, the introduction of immune checkpoint inhibitors (ICIs) has revolutionized cancer treatment.^36^ Specifically, cytotoxic T-lymphocyte-associated protein 4 (CTLA-4) and programmed cell death protein 1 (PD-1) are inhibitory receptors found on the surface of T cells. Programmed death-ligand 1 (PD-L1) is a ligand mainly found on cancer cells that binds PD-1. These molecules are part of the immune checkpoint pathways that regulate immune responses, particularly by preventing overactivation of T cells, resulting in enhanced resistance of cancer cells to endogenous anti-tumor immune responses. ICIs are treatments that block these pathways (*e.g.*, anti-CTLA-4, anti-PD-1, and anti-PD-L1 therapies) to enhance the immune system's ability to detect and kill cancer cells, leading to significant advancements in cancer treatment.^37,38^ Nevertheless, these therapies do not elicit uniform responses across patients, as their efficacy strongly depends on tumor type, microenvironment, and individual immune variability.

#### 2.1.2 Tumor resistance mechanisms

Despite this growing arsenal of treatments, the dynamic evolution of tumors often leads to the development of therapy-resistant cellular clones.^39^ From the cellular point of view, therapy-resistant subpopulations are often enriched in cancer stem cells (CSCs) or tumor-initiating cells (TICs) phenotypes; this group of cells constitutes a small population within tumors, known for their ability to self-renew and proliferate indefinitely.^40^ Their plasticity and ability to dynamically adapt to environmental cues, often described as tumor plasticity, are central contributors to treatment failure, metastasis, and immune evasion.^41–43^ Additionally, the inherent resistance of CSCs significantly contributes to cancer recurrence.^40,44^

Resistance to anticancer therapies is commonly classified as either primary or secondary.^45^ Primary (intrinsic) resistance refers to an inherent insensitivity of cancer cells to a given treatment, often due to a lack of dependence on the therapeutic target or the presence of pre-existing alternative survival pathways, resulting in reduced treatment effectiveness.^46^ Secondary or acquired (extrinsic) resistance, in contrast, arises as an adaptive response to therapy. It develops over time through selective pressure that drives genetic or epigenetic changes, enabling tumor cells to evade treatment effects. The mechanisms underlying resistance are diverse and still not fully understood.^47,48^

In the context of targeted therapies, resistance is typically acquired (*i.e.*, secondary) and associated with mutations in the drug target, activation of bypass signaling pathways, or enhanced tumor cell plasticity. Similarly, chemotherapy often leads to acquired resistance, which is considered to be responsible for up to 90% of treatment failures^49,50^ and a major cause of cancer-related mortality.^51^ When first-line treatments fail, patients must rely on second-line chemotherapy regimens, which, however, are often associated with limited effectiveness and poorer prognosis.^52,53^ The lack of validated predictive biomarkers also means chemotherapy is frequently administered without clear evidence of clinical benefit, exposing patients to potentially avoidable toxicity and side effects unnecessarily.^54^

In contrast, resistance to immunotherapy is frequently intrinsic (*i.e.*, primary), shaped by pre-existing tumor–host–microenvironment interactions such as low T cell infiltration, defective antigen presentation, or immunosuppressive signaling. Nevertheless, resistance can also develop after an initial response, particularly in cases of partial response, illustrating a transition from primary to secondary resistance.^55^ Overall, despite the promising results in responders, clinical benefit is restricted to a subset of patients, with reported response rates ranging from 20% to 40%.^46,56,57^

Radiotherapy is also commonly associated with primary resistance, with concerning treatment failure given its widespread use (adopted in around 50% of cancer patients^58^). Approximately 40% of patients experience recurrence within five years of treatment, and 15–40% of survivors are at risk of developing secondary malignancies in the irradiated area. These recurrences significantly contribute to overall cancer-related mortality.^59–61^ The current studies suggest that radiation resistance is associated with multiple factors, including tumor heterogeneity, adaptation of the TME, and genetic alterations produced by the incident radiation.^33,62–64^

Besides the traditionally recognized role of genetic mutations in tumor resistance,^41^ increasing evidence emphasizes the importance of non-genetic mechanisms, including epigenetic remodeling, phenotypic plasticity, and dynamic cell state transitions.^65,66^ For instance, some cancer cells can adopt a drug-tolerant persistent state through metabolic reprogramming, allowing them to survive under therapeutic stress, a phenomenon known as the “resistance continuum”.^39,67^ A prominent example of these non-genetic adaptive cellular states is therapy-induced senescence (TIS),^68^ a state in which tumor cells, although metabolically active, permanently exit the cell cycle in response to cytotoxic stress such as DNA damage, oxidative injury, or oncogene activation. These senescent cells are characterized by distinct morphological changes, chromatin remodeling, and the release of bioactive factors known collectively as the senescence-associated secretory phenotype (SASP), which can profoundly reshape the TME by modulating immune responses, inflammation, and tissue remodeling.^69^ While TIS initially contributes to tumor suppression by arresting proliferation and enabling immune-mediated clearance, its long-term presence may paradoxically promote tumor progression, immune evasion, and therapeutic resistance, thereby complicating effective cancer treatment.^5^

In general, as described by Holohan *et al.* (2013) and supported by Lei *et al.* (2023), tumor resistance mechanisms commonly involve increased drug efflux, target mutations, enhanced DNA damage repair,^70^ the activation of alternative survival pathways, evasion of cell death, drug inactivation, reduced drug uptake, tumor plasticity, and modulation by the TME, as shown in Fig. 1.^51,71^

#### 2.1.3 Role of the tumor microenvironment

The TME refers to the complex and dynamic ecosystem surrounding tumor cells; it is shaped by a diverse range of cells, including tumor cells and immune cells, as well as structural and stromal components such as cancer-associated fibroblasts (CAFs), the extracellular matrix (ECM), adipocytes, and chemokines (Fig. 2, left panel), all sustained by an extensive vascular network.^72–74^ The spatial organization of this complex niche contributes to microenvironmental heterogeneity (explained in Section 2.1.1), influencing tumor behavior, therapeutic responses, and ultimately immune regulation and tumor-associated immunosuppression.^41,73,75–77^

**Fig. 2:**
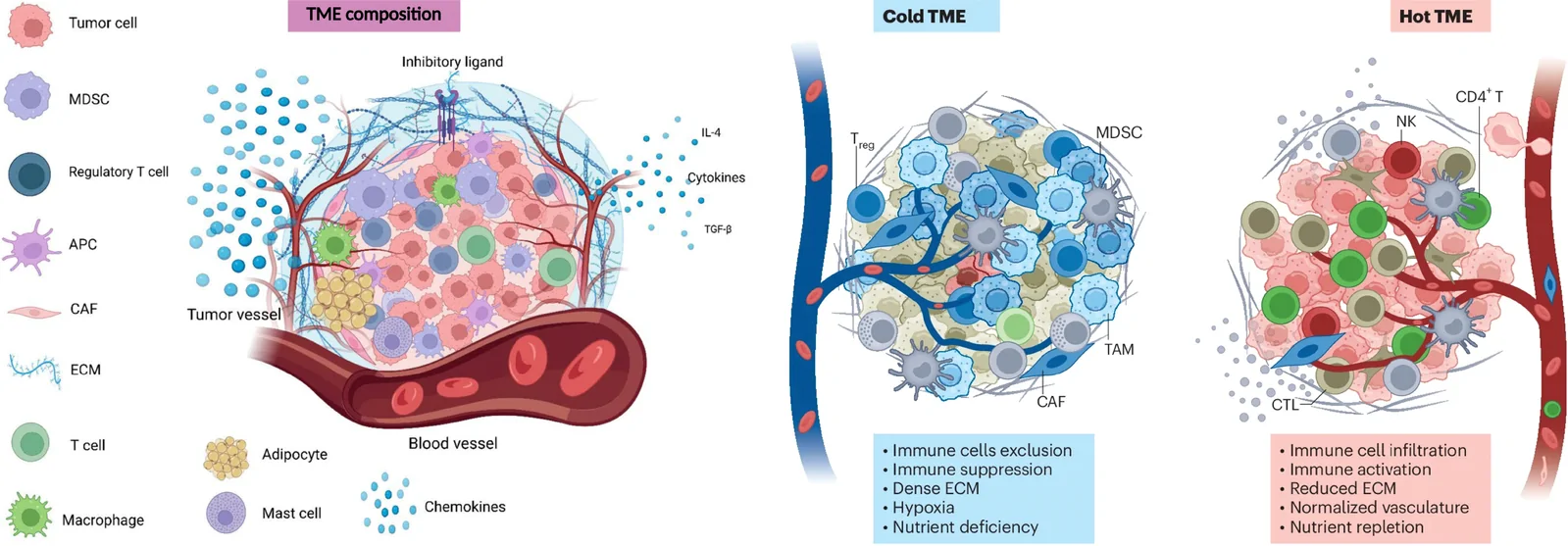
Schematic representation of tumor microenvironment composition and immune landscape. Left panel: General TME showing tumor cells, stromal components (cancer-associated fibroblasts [CAFs] and extracellular matrix [ECM]), immune cells (T cells, regulatory T cells, macrophages, mast cells, myeloid-derived suppressor cells [MDSCs], antigen-presenting cells [APCs]), adipocytes, blood vessels, and chemokines/cytokines (adapted from Khosravi *et al.*, 2024^73^ exclusive licensee Sun Yat-sen University Cancer Center. Distributed under a CC BY-NC-ND 4.0 license). Middle panel: Cold TME, characterized by immune exclusion, suppression, dense ECM, hypoxia, and nutrient deficiency, featuring tumor-associated macrophages (TAMs). Right panel: Hot TME, characterized by immune infiltration, activation, reduced ECM, normalized vasculature, and nutrient repletion, highlighting natural killer (NK) cells, CD4+ T cells, and cytotoxic T lymphocytes (CTLs). Middle and right panels adapted from Yuan *et al.* (2025)^80^ with permission from Springer Nature.

The specific relationship between the TME and the immune system is a primary determinant in predicting therapy response, particularly through the presence of infiltrating T cells, which classify tumors as either cold or hot. Cold tumors (Fig. 2, central panel) are characterized by a lack of T cell infiltration, resulting in limited immune recognition and tumor attack. Immunological treatment of cold tumors is a significant challenge, as no adaptive immune response has been established or maintained, making them less responsive to standard immunotherapies.^78,79^ In contrast, a hot TME is characterized by high infiltration of active immune cells, such as cytotoxic T lymphocytes and NK cells, which not only promote an effective anti-tumor immune response,^73,80^ but also influence radiotherapy and certain chemotherapies requiring an active immune response for optimal efficacy, such as in immunogenic cell death.^81^ Therefore, the overall TME is closely connected to the growth, invasion, and spread of tumor cells.^26^

Given the complex relationship between the TME and treatment prognosis, the TME has emerged as a potential source for identifying biomarkers of therapy response. For example, in the case of immunotherapy, major efforts have been invested in identifying biomarkers associated with a positive response, such as PD-L1,^82^ microsatellite instability, and tumor mutational burden (TMB), as well as common indicators in clinics such as tumor proportion score (TPS), immune cell score (IC) and combined positive score (CPS), based on the percentage (%) of cells expressing a specific ligand. However, the predictive value of these biomarkers remains limited. For instance, PD-L1 expression is highly dynamic and can change over time in response to previous treatments, radiotherapy, or evolving immune interactions.^83,84^ In addition, the marked spatial heterogeneity of solid tumors means that a single biopsy often fails to capture the full molecular landscape of the lesion, potentially leading to inaccurate patient stratification. Consequently, clinically validated biomarkers capable of reliably predicting treatment response and guiding personalized cancer therapy remain scarce. To date, only a limited number have been incorporated into routine clinical practice, with PD-L1 expression status remaining one of the most widely used biomarkers for selecting patients eligible for anti–PD-L1 immunotherapy despite its well-recognized limitations.^55,85^

Therefore, accurate diagnosis and early assessment of treatment response are essential for guiding treatment selection and identifying cell populations that may eventually develop therapy resistance. Furthermore, the discovery of predictive biomarkers, comprehensive characterization of the tumor microenvironment, and mechanistic studies in preclinical models are critical for understanding the biomolecular basis of therapeutic response and resistance.^86^

#### 2.1.4 Gold standard methods for therapy-response monitoring in patients

The *in vivo/in situ* therapy response monitoring in clinical practice predominantly relies on computed tomography (CT), positron emission tomography-computed tomography (PET-CT) scans, and magnetic resonance imaging (MRI). These tumor imaging modalities are used to evaluate tumor burden by capturing both large-scale (macroscopic) and microscopic features of the tumor. Macroscopically, this includes measurements like tumor size and shape, which can be quantified using one-dimensional (longest diameter), two-dimensional (area), or volumetric methods.^87^ Furthermore, advanced image analysis can reveal microscopic features such as tumor texture heterogeneity. At the cell level, evaluation relies on the histology of tumor biopsies, which assesses tissue architecture and the expression of targeted markers based on immunohistochemistry (IHC) or immunofluorescence (IF) staining intensity. However, while histological imaging is the gold standard for initial diagnosis, it is rarely employed for continuous therapy response monitoring due to its invasive nature. Therefore, treatment response is commonly assessed by comparing CT images acquired before and after therapy, and tumors are typically classified into four categories: complete response, partial response, stable disease, or progressive disease. These classification systems may differ depending on tumor type and treatment strategy.^88^

CT imaging, however, cannot reliably distinguish viable tumor from necrotic or fibrotic tissue, lacks sensitivity to molecular and cellular treatment responses, and often fails to detect microscopic disease, such as minimal residual disease (MRD). These limitations may lead to inaccurate assessments of treatment effectiveness,^89^ particularly during the early stages of targeted therapies and immunotherapies, where tumor size may not be a reliable indicator due to pseudo-progression resulting from T cell activation.^90^ On the other hand, PET struggles with distinguishing active tumor tissue from treatment-related changes such as inflammation or necrosis, leading to potential false positives or negatives, and it also has lower spatial resolution compared to MRI. In addition, PET's high cost and limited availability can restrict its routine use. Considerable efforts have been made in the recent years to improve these limitations using PET-metabolic imaging.^92–94^

MRI, while offering superior anatomical detail, has limited functional assessment capabilities and can be affected by post-treatment changes like edema or scarring, making it difficult to differentiate residual tumor tissue from therapy-related effects. Furthermore, the use of contrast agents in MRI may pose risks for patients with renal impairment or allergies.^95^ To address these gaps, extensive research has focused on identifying biomarkers in body fluids, such as proteins, cytokines, metabolites,^96^ hormones, microRNAs, and circulating DNA,^91^ mainly in blood,^97^ the most commonly collected minimally invasive clinical sample.^86,98^

The current limitations in therapy-response monitoring may expose patients to the harmful side effects of ineffective therapies, while the biomolecular effects producing therapy resistance remain unknown. Therefore, due to the difficulties in understanding the underlying mechanisms of therapy response and resistance, preclinical models, including *in vitro*, *ex vivo*, and *in vivo* approaches, have been developed to simulate therapeutic effects and elucidate the molecular pathways involved in treatment response. All currently established clinical imaging modalities primarily assess morphological or metabolic consequences of therapy rather than directly measuring therapy-induced molecular alterations.

#### 2.1.5 Therapy response studies in preclinical experimental models

The most common *in vitro* evaluation of therapies involves the use of metabolic assays (such as MTT, MTS, XTT, or resazurin/Alamar Blue), usually measuring cell viability after exposure to a therapeutic intervention. However, these protocols do not provide insights into the spatial distribution of biochemical features of *in vitro* cell cultures. To obtain this information, bioimaging is primarily performed using fluorescence microscopy, which typically requires fluorescent tags or labels in fixed cells, ultimately altering their original behavior.^99^

To better reproduce tumor physiology, more advanced preclinical models have been developed. Three-dimensional cultures such as spheroids and organoids provide a more realistic architecture and cell–cell interactions compared to 2D cultures, while patient-derived organoids (PDOs) maintain patient-specific heterogeneity.^100^ Patient-derived xenografts (PDXs), obtained by implanting human tumor tissue into immunocompromised mice, and genetically engineered mouse models (GEMMs) allow the evaluation of therapy efficacy in a physiologically relevant context.^101–103^ Additionally, in recent years, there has been growing interest in evaluating standardized *ex vivo* tissue culture models, also referred to as the *ex vivo* tumor fragment platform^104^ by some authors, to investigate individual tumor responses.^105,106^

In the case of *ex vivo* evaluation of 3D models like human PDOs and xenografts, the traditional gold standard for assessing therapy effectiveness is histopathological imaging obtained through staining methods (primarily Hematoxylin and Eosin, H&E) and IHC assays on sliced organoids or sliced xenografted tumors. This protocol involves several steps: after treatment, the tumor xenograft is extracted and fixed, embedded, and sectioned. Then, tissue morphology is evaluated using H&E staining and specific immunohistochemical stains, using labeled antibodies.^4,107^ However, this methodology presents some limitations; the H&E-based approach is neither quantitative nor objective, relying on the pathologist's interpretation, and does not allow precise molecular characterization of individual tumors or the detection of molecular changes in tumor evolution under targeted therapy. In the case of IHC, the limitations are related to the poor reproducibility among pathologists, the tendency to reduce marker expression assessment to a binary result lacking a clear positive threshold and the inaccurate counting/judging of the number of cells expressing a determined biomarker (commonly using % of positive cells). Thus, there is a need for objective, label-free and spatially resolved methodologies capable of characterizing tumor heterogeneity and the TME through comprehensive biochemical fingerprinting.

#### 2.1.6 Therapy response prediction

Predicting therapy outcomes is a primary objective of treatment-monitoring studies, as these inherently rely on statistical models designed to anticipate patient response. Clinically, such predictions are mainly supported by tumor screening, which can involve invasive procedures like tissue biopsy or non-invasive imaging techniques.^86^ Tumor biopsies allow for the extraction of malignant tissue, enabling characterization of the tumor tissue, including tumor type, grade, tissue architecture, invasion patterns. Furthermore, the pathological evaluation of tumor response after neoadjuvant therapy (referred in clinics as pathological response, PR) represents a particular challenge, as pathologists must determine whether viable tumor cells remain, a distinction that is often difficult to establish.^108–110^

The TME is commonly analyzed *via* immunohistochemistry for marker expression,^111^ mutation detection *via* PCR or genomic methods (including next-generation sequencing), transcriptomic profiling (mRNA expression),^112,113^ and protein quantification using ELISA, western blot, or mass spectrometry. For instance, Thompson *et al.* (2025) recently identified a set of chromosomal instability signature biomarkers capable of predicting resistance to platinum-, taxane-, and anthracycline-based therapies through a single genomic assay.^54^ Other examples are clinically used, as previously mentioned, in immunotherapy; IHC staining is typically used to assess PD-L1 expression and guide patient selection for anti-PD-1/PD-L1 therapies.^114^ However, these protocols present important limitations: immunohistochemistry results vary across assays, antibody clones, and cell-type–specific expression patterns, and still rely on semi-quantitative, operator-dependent scoring. In the case of PD-L1, clinical interpretation is further hindered by heterogeneous and inconsistently defined positivity thresholds, reducing reproducibility across laboratories.^115^ To address these limitations, omics-based approaches have been recently proposed. These methodologies, while offering broader molecular insights, require extensive sample processing, are generally destructive, and lack spatial resolution—except for advanced single-cell technologies, which remain limited to specialized laboratories. Likewise, fluorescence microscopy is constrained by single-marker detection and the potential interference of fluorescent tags with protein function, as previously discussed.^99^ In parallel, significant advances have been made toward integrating artificial intelligence^116^ to enhance prediction accuracy by combining clinical data with molecular tumor profiles: multistain deep learning approaches for prediction of prognosis and therapy response in colorectal cancer;^117^ machine learning models predicting immunotherapy response by integrating genomic, molecular, demographic and clinical data across 16 cancer types;^118^ multi-omics machine learning predictors of chemotherapy response in breast cancer,^119^ and deep learning approaches assessing therapy-induced morphological changes in metastatic colorectal cancer.^120^

#### 2.1.7 Envisaging ideal label-free, chemically specific, and minimally perturbative technologies for cancer therapy monitoring

In an ideal scenario, tools for monitoring and predicting cancer therapy response would integrate seamlessly with biological systems, providing real-time biochemical information without disturbing the samples. These technologies should enable continuous observation of therapy-induced molecular and metabolic changes in cells, organoids, and tumors, preserving their native state and avoiding labels or stains that may alter biological processes, suffer from human error and operator bias, require additional time and resources, and may misrepresent the original biomolecular composition. Such tools should be non-destructive, ideally non-invasive or minimally invasive, label-free, and capable of extracting molecular signatures directly from intrinsic tissue properties. Photonic techniques are well positioned to meet these requirements, offering high biochemical specificity and compatibility with both microscopy platforms and portable formats such as fiber optic probes. This versatility would allow their use in intraoperative procedures, endoscopy, and other real-time clinical applications.

Adaptability is also essential. Ideally, these systems would function across research and clinical environments, enabling applications such as continuous monitoring of tumor biopsies in culture or real-time assessment of tumor margins. Their ability to detect emerging resistance mechanisms or inadequate responses would significantly improve experimental workflows and patient management. Finally, these technologies should support personalized treatment decisions. By capturing tumor-specific biochemical profiles and tracking early therapeutic responses, they could help clinicians anticipate treatment efficacy and adjust regimens promptly. Early identification of resistance would enable proactive therapeutic modifications. Overall, the future of therapy monitoring and prediction relies on tools capable of delivering precise, real-time biochemical insights without disrupting biological systems. Raman-based approaches are particularly well suited to fulfill these requirements owing to their ability to provide label-free, chemically specific, and spatially resolved information across a wide range of biological systems. A detailed overview of Raman spectroscopy and its relevance to this field follows in the next section.

## 3 Fundamentals and modalities of Raman spectroscopy

Raman spectroscopy has been increasingly applied to studies on cancer therapy response due to its label-free nature and ability to generate chemical images that have positioned this technique as a powerful tool in biomedicine.^18^ This section outlines the fundamental principles of Raman spectroscopy, from the physics of light–matter interactions to the description of different label-free modalities, such as spontaneous Raman (SR), SERS, CARS, and SRS (Section 3.1). In this review, the term spontaneous Raman spectroscopy (SR) is used, consistently with common terminology in the biomedical Raman literature, to refer to conventional Raman spectroscopy based on incoherent Raman scattering. Although Raman scattering is not a spontaneous emission process in the strict physical sense, this terminology is widely adopted to distinguish it from coherent Raman techniques, such as SRS and CARS. Furthermore, we review its current applications in biomedicine (Section 3.2), provide guidelines for selecting the most appropriate Raman approach for specific studies (Section 3.3), and discuss the usability of current state-of-the-art multimodal setups involving Raman-based approaches (Section 3.4).

### 3.1 Principles and mechanisms of Raman spectroscopy

Raman spectroscopy is a non-destructive analytical technique that uses the inelastic scattering of light to probe molecular vibrations, providing information about the chemical composition of a sample. It is particularly adequate for the study of biological samples due to its ability to detect subtle molecular changes without the need for labels or dyes.^121^ Raman spectroscopy relies on the interaction between monochromatic light and the vibrational modes of molecules. When a photon from the laser beam interacts with a molecule, most of the scattered light undergoes Rayleigh scattering, where the photon is scattered without any energy change, maintaining its original frequency. However, a small fraction (1 out of 10^6^–10^8^) of the scattered photons experiences an inelastic scattering event, known as Raman scattering, where an energy exchange between photons and the molecule occurs. This energy transfer produces a shift in the frequency of the scattered photons, known as Stokes (lower energy) and anti-Stokes (higher energy) shifts, which correspond to the vibrational modes of the molecules. This generates a characteristic spectral fingerprint that can be used to identify the chemical composition of the sample.^122^ The experimental observation of the “Raman effect” was reported in 1928 by C. V. Raman and K. S. Krishnan in India^123^ and almost simultaneously in Russia by Landsberg and Mandelstam where it was termed “combinational scattering of light”.^124,125^ These experimental observations followed earlier theoretical descriptions of inelastic light scattering by A. Smekal, H. A. Kramers, W. Heisenberg, and P. A. M. Dirac.^126–128^

To fully understand the inelastically scattered Raman photons, Raman scattering can be described using two complementary but equivalent frameworks: a classical and a semi-classical approach. In the classical one, molecular vibrations are modeled as harmonic oscillations of atoms connected by chemical bonds, which can be approximated as springs characterized by a stiffness constant and a reduced mass. The vibrational frequencies of these oscillators depend on the bond strength and the masses of the atoms involved, providing direct molecular specificity. When an oscillating electromagnetic field interacts with a molecule, it induces a dipole moment described by *p* = *αE*, where *p* is the induced dipole moment, *α* is the molecular polarizability, and *E* is the electric field. Crucially, Raman scattering occurs only if the polarizability varies with the vibrational coordinate, which defines the Raman selection rule. This interaction generates scattered light components at the incident frequency (Rayleigh scattering) and at frequencies shifted by the vibrational modes of the molecule, corresponding to Stokes and anti-Stokes Raman signals.

Within a semi-classical framework, molecular vibrations are described as discrete energy levels of a quantum harmonic oscillator, and Raman scattering is treated as a light–matter interaction governed by perturbation theory. In this approach, the incident radiation induces transitions between vibrational states *via* virtual energy levels, resulting in the emission of photons with energies shifted by one vibrational quantum. Stokes scattering occurs at a frequency *ν*_S_ = *ν*_ex_ − *ν*_m_, corresponding to transitions to higher vibrational states, whereas anti-Stokes scattering occurs at a frequency *ν*_AS_ = *ν*_ex_ + *ν*_m_, arising from transitions originating from thermally populated excited vibrational states, where *ν*_ex_ represents the incident light frequency and *ν*_m_ the molecular vibrational frequency. As dictated by Boltzmann statistics, the population of these excited states is low at ambient temperatures, explaining the typically weaker intensity of anti-Stokes signals compared to Stokes signals. Despite their different formalisms, both classical and quantum descriptions converge on the same physical interpretation: Raman spectra arise from vibrational modes that modulate molecular polarizability, yielding chemically specific spectral fingerprints that support the analytical power of Raman spectroscopy. For readers interested in a more comprehensive description of the theoretical foundations of Raman scattering, including the classical description of induced molecular polarization and Stokes/anti-Stokes processes, further details can be found in dedicated Raman spectroscopy textbooks.^129^

### 3.2 Raman spectroscopy in biomedicine

In the current research landscape, the use of Raman-based technologies has been extended to studies involving drug distribution, response and drug resistance using both Raman imaging^7^ and spectral-based approaches.^19^ Historically, one of the first Raman maps of human samples was produced in 1997, when Otto *et al.* reported the acquisition of Raman images of tissues, specifically identifying the interface between healthy and cataract-affected regions of the eye lens.^130^ A few years later, in 2002, Raman maps of cancerous tissues were presented by Bakker Schut *et al.*^131^ Over time, Raman spectroscopy has evolved from single-point *in vitro* analysis to near-real-time chemical imaging of tumors during surgery, a rapidly expanding field in oncological diagnostics thanks to the development of deep learning and AI-based models.^13,132^ Thus, the use of Raman spectroscopy for the study and assessment of cancer has been increasingly enhanced over the past 15 years.^13,133–135^

A major driver of this growth is that Raman spectroscopy is not restricted to predefined molecular targets, in contrast to antibody-based methods such as IHC, including immunofluorescence. Instead, it provides a holistic, label-free molecular profile that captures both the biochemical and morphological complexity of biological samples, particularly when spatially resolved measurements are performed. This enables the detection of relevant biochemical changes in tissues, cells, or biofluids.^121,136^ In this sense, Raman spectroscopy is conceptually related to omics approaches such as proteomics, lipidomics, or transcriptomics. However, while omics techniques can identify tens of thousands of individual biomolecules, they are intrinsically destructive and require extensive sample processing. By contrast, Raman spectroscopy detects a limited amount of information but enables direct, non-destructive, and label-free analysis by probing intrinsic molecular vibrations, while offering high spatial resolution and minimal sample preparation, making it suitable for high-quality imaging of live cells and tissue sections,^18,137^ and competitively fast when imaging is not required. A notable example of clinical translation is the recent development of *FastGlioma*, an AI-powered model, which, for the first time, enables ultrafast (<10 s), label-free detection of glioma infiltration in fresh, unprocessed surgical tissue using Raman-based imaging to monitor cancer surgery.^14^

Beyond histopathology, the integration of deep learning with Raman spectroscopy has recently demonstrated the ability to predict single-cell RNA sequencing profiles from label-free hyperspectral Raman images, as shown by the Raman2RNA model developed by Kobayashi-Kirschvink *et al.*^138^ Together, these advances highlight the transformative potential of Raman spectroscopy not only for therapy response monitoring and prediction, but also for a broad range of applications across the biomedical sciences.

#### 3.2.1 Modalities

The most common label-free Raman-based approaches used in therapy monitoring, as observed in the works reviewed in the literature, and shown in Fig. 3a, include SR, SERS, and nonlinear techniques such as SRS and CARS.

**Fig. 3:**
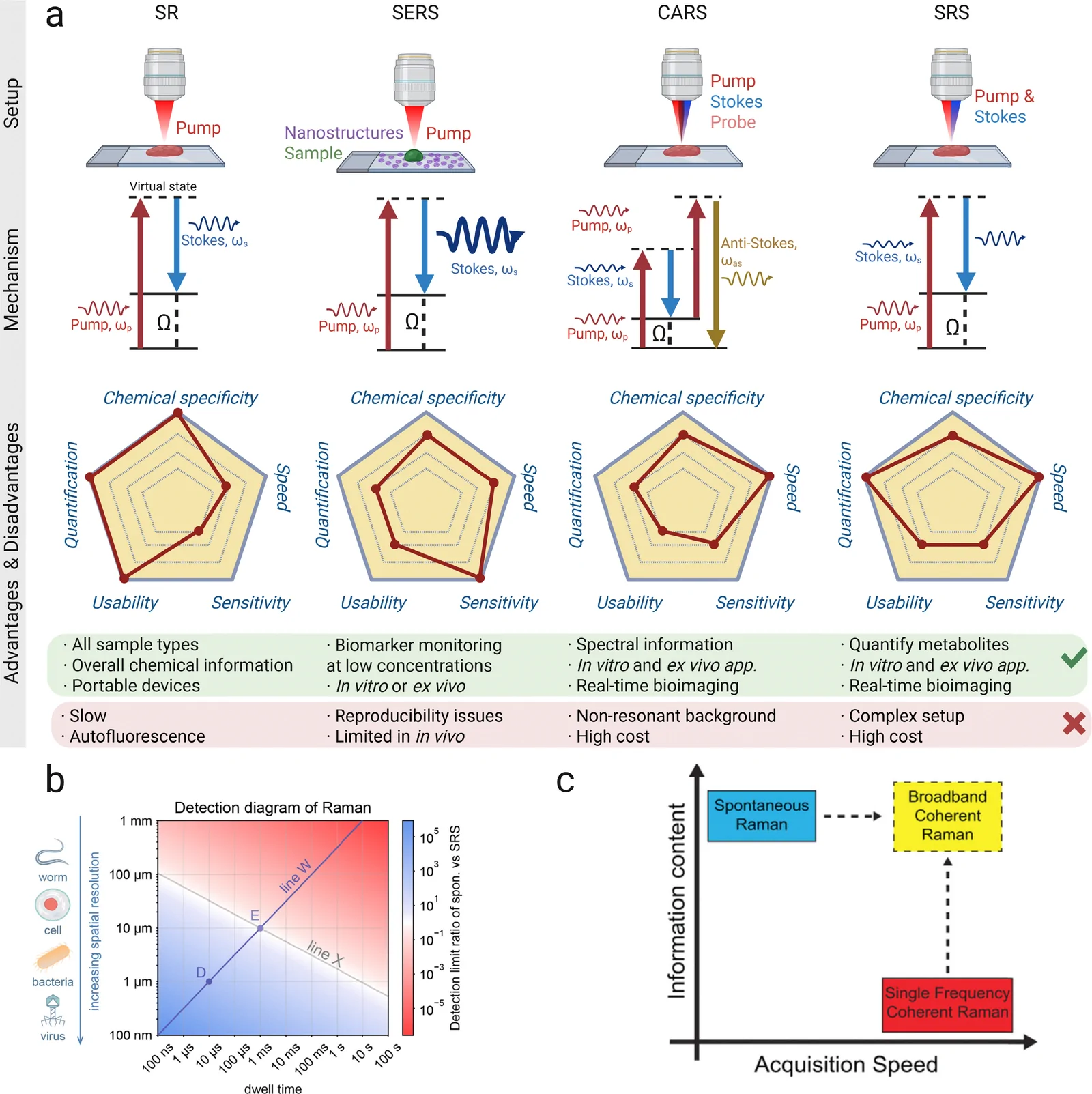
Overview of the most common Raman-based approaches: (a) setup, energy-level diagram of Raman scattering (mechanism), and advantages & disadvantages of each Raman approach; (b) detection diagram on spatiotemporal axes for SRS and SR comparison, adapted from Gao *et al.* (2025)^204^ with permission from the American Chemical Society; and (c) comparison between spontaneous and coherent Raman for imaging speed and level of chemical information, adapted from Polli *et al.* (2018)^151^ with permission from Wiley-VCH.

SR is the conventional and widely used Raman spectroscopy modality. It involves illuminating the sample with a monochromatic laser and collecting scattered light (both typically using a microscope objective), and filtering out Rayleigh scattering with optical filters. The remaining inelastically scattered light is then dispersed by a spectrometer, and Raman spectra are typically detected using a charge-coupled device (CCD).^139^ However, CCD-free^140^ or spectrometer-free^141^ setups have recently been proposed in the literature. SR, compared to other Raman modalities, provides nowadays the richest and most accurate molecular information based on vibrational energy shifts, enabling detailed analysis of a sample's biochemical composition non-destructively and using a relatively simple, accessible and commercially available instrumentation.^4^ Its main limitations include long acquisition times due to the incoherent nature of Raman scattered photons and therefore a limited number of pixels that can be captured within the field of view of interest in a reasonable amount of time, thus limiting the spatial resolution and/or the size of the scanned sample (especially problematic for large tissue areas or multiple cell populations).

To overcome these challenges, Coherent Raman Scattering (CRS) techniques emerged, with the first report of a coherent Raman microscope between 1999 and 2000,^142^ which triggered the generation of an independent research and application field that is continuing to revolutionize biomedical imaging. CRS techniques refer to a family of nonlinear optical methods that probe molecular vibrations through the coherent interaction of two (or three) synchronized ultrashort laser pulses at different frequencies, generating amplified signals and overcoming the speed limitations of SR spectroscopy while enabling 3D optical sectioning thanks to its intrinsic non-linear nature. The two main CRS modalities are coherent anti-Stokes Raman scattering (CARS) and stimulated Raman scattering (SRS), both capable of rapid imaging, volumetric mapping, and dynamic monitoring of biological processes in near-real time.

SRS requires two ultrashort-pulsed lasers, typically featuring few-picosecond pulse durations: a pump laser (at frequency *ω*_p_) and a Stokes laser (at frequency *ω*_S_), often in the near-infrared region (800–1000 nm) to ensure low phototoxicity on biological samples.^143,144^ The pump and Stokes pulses are spatio-temporally overlapped and focused onto the sample through a microscope objective, triggering a coherent vibration when the frequency detuning *Ω* = *ω*_p_ – *ω*_S_ matches a Raman-active vibrational mode, providing substantially higher signal levels than SR. One of the two pulses is typically modulated at a high frequency (*i.e.*, tens of MHz) to implement a modulation-transfer detection scheme using lock-in amplification: the SRS signal consists of a small gain or loss in intensity (in the order of 10^−4^–10^−6^) at Stokes or pump beam intensities, respectively. This setup, despite the high complexity and relatively high costs to ensure performance, enables near real-time chemical imaging of preselected Raman shifts. The majority of SRS systems reported in the literature and most commercially available devices operate in a continuously tunable single-Raman-shift acquisition mode, producing Raman intensity images at one selected Raman shift at a time. Alongside conventional stimulated Raman gain or loss configurations, photothermal SRS approaches have recently emerged as an alternative detection strategy, exploiting laser-induced refractive index changes associated with vibrational relaxation.^145^ Because pixel dwell times are reduced to a few microseconds (for a single Raman shift) instead of the hundreds of milliseconds required in SR (for collecting the entire vibrational spectrum), tissue slices of several millimeters or hundreds of cultured cells can be imaged in minutes rather than hours. However, it is important to note that this profound speed advantage relies heavily on *a priori* knowledge of the target Raman peaks. If the spectral targets are unknown and broadband hyperspectral imaging is required across large tissues, the necessity of acquiring successive image stacks at every Raman shift severely diminishes the speed advantage of SRS, bringing overall acquisition times much closer to those of SR.^13,146,147^ In this framework, label-free SRS virtual H&E staining (also called stimulated Raman histology, SRH)^148^ surged and translated to intraoperative use in recent years: the nuclei-cytoplasm color coding is quickly achieved with two sequential SRS scans (the 2850 cm^−1^ mode of CH_2_ and CH_3_ stretching in proteins and lipids, and the 2930 cm^−1^ mode of CH_3_ stretching in proteins).^13,149,150^

More recently, several solutions have been proposed to perform broadband SRS imaging, enabling the acquisition of hyperspectral Raman maps using SRS. For each pixel, a spectrum is provided, covering a relatively large spectral region, such as the entire CH region or, in some cases, also the fingerprint one.^151^ Broadband SRS can be achieved using two alternative schemes. The first is the so-called hyperspectral SRS modality, which detects a single Raman vibrational frequency at a given time and then sequentially scans the pump–Stokes detuning. Most of the practical implementations of this scheme are based either on fast tuning the central wavelength of the (narrowband) pump or Stokes beam, or on the so-called spectral-focusing SRS, which employs highly-chirped broadband pump and Stokes beams and tunes the Raman shift sequentially by varying their relative delay. While effective for measuring a broad Raman spectrum, the acquisition is sequential in hyperspectral SRS, so that it hinders fast imaging when a broad SRS spectrum is required. On the other hand, the spectrum can be measured in parallel using the so-called multiplex approach, which detects the full SRS spectrum. This is in general achieved using a multi-channel lock-in amplifier detector,^152^ which may introduce a significant degree of complexity and therefore limit plug-and-play user experience.^4,151^

An alternative modality to SRS is CARS. It employs the very same synchronized pulsed lasers as for SRS, but the signal detected consists of anti-Stokes photons emitted at the frequency *ω*_aS_ = *ω*_p_ + *Ω*. Since this emitted signal is blue-shifted relative to the incident laser beams, it can be easily isolated *via* optical filtering from the pump and Stokes and does not require modulation transfer and lock-in amplifiers (so-called background-free signal), which simplifies the optical scheme compared to SRS and improves signal specificity compared to SR.^153^ A key limitation, however, is the presence of a non-resonant background (NRB), originating from competing four-wave mixing interactions that do not involve the vibrational modes of the sample.^139^ This background is a non-specific signal that distorts the vibrational peaks of CARS spectra and reduces the signal-to-noise ratio, especially when tackling lower intensity Raman modes in the fingerprint region. While traditional removal of the NRB in CARS spectra relies on expert-driven numerical algorithms and prior knowledge of the NRB shape, recent deep-learning models proved automated, accurate, and real-time NRB subtraction possible.^154^ However, concerns remain regarding the generalizability of such models across different systems and samples, as they require large amounts of data to train and are computationally intensive to run. While broadband SRS microscopy is rather complex and no reports are present in literature demonstrating multiplex SRS capable of detecting the entire vibrational spectrum in parallel (*i.e.* simultaneously), as it is normally achieved in SR, several broadband CARS (BCARS) microscopes have been reported, delivering broadband hyperspectral Raman spectra over the entire fingerprint and CH-stretching regions in a single shot.^155–159^ Besides the larger amount of information, broadband CARS makes the NRB-removal task easier because of the complete spectral shape available. On the other hand, it typically uses a broadband pulse with ultrashort duration (*i.e.*, up to a few hundred femtoseconds), which implies additional optical elements for dispersion and chirp management, increasing the complexity of the system for end users. Despite these hurdles, broadband CARS and SRS approaches alike are promising for fast, high-content, quantitative imaging of cancer tissue.^160^

In parallel, coming back to the SR regime, surface-enhanced Raman spectroscopy (SERS) is a technique that enhances the inherently weak Raman signal of SR techniques by leveraging the plasmonic properties of metallic nanostructures.^161^ When molecules, even at extremely low concentrations, are brought near metallic nanostructures and excited by a laser of a specific wavelength, the Raman signal is significantly amplified.^162^ In the case of label-free SERS, the signal intensity is influenced by the Raman cross section of the molecule, which is typically low for most molecules.^163^ SERS typically enhances Raman signals by exploiting localized surface plasmon resonances in noble metal nanostructures, such as gold or silver, which generate strong electromagnetic fields and boost sensitivity by several orders of magnitude.^162^ In complex matrices, such as liquid biopsies and other biofluids, the SERS signal is strongly influenced by analyte–surface interactions, and molecules with higher affinity for the metal surface may contribute disproportionately to the measured spectrum.^164,165^

Finally, spatial offset Raman spectroscopy (SORS) is an alternative, also in the SR regime, that helps address penetration-depth issues of Raman approaches. In SORS, photons from deeper layers tend to migrate laterally before emerging at the surface, while those from shallow regions are more likely lost at surface interfaces.^166,167^ By collecting Raman signals away from the laser spot, SORS enhances sensitivity to subsurface regions. The distance between illumination and collection points, known as the spatial offset (Δ*s*), determines the probing depth—larger offsets favor deeper signal contribution.^167–169^ Like SR imaging, SORS entails lengthy acquisition times, impairing its widespread application in volumetric biomedical imaging. The combinations of the above-mentioned SERS and SORS approaches^170^ (surface-enhanced spatially offset Raman spectroscopy, SESORS, or surface-enhanced spatially offset resonance Raman spectroscopy, SESORRS) have recently emerged as a promising approach to detect low-concentration molecules at different penetration layers in tumors.^171,172^

#### 3.2.2 Raman signal acquisition strategies

In terms of Raman signal acquisition strategies, Krafft *et al.* (2016) conducted a thorough review covering a wide range of methods developed for capturing Raman signals in biological samples.^139^ Among the most frequently employed approaches are confocal Raman microscopy, laser-tweezers Raman spectroscopy (LTRS), point-scanning, line-scanning and wide-field illumination. Selecting the appropriate acquisition technique is essential to optimize signal strength, reduce background interference, and generate data that are both interpretable and reproducible. Confocal Raman microscopy uses a focused laser spot and a pinhole to achieve high spatial and optical resolution, enabling 3D imaging by rejecting out-of-focus signals.^173^ Laser-tweezers Raman spectroscopy (LTRS) combines optical trapping and Raman analysis to manipulate and measure single suspended cells without fixation; Moritz *et al.* (2010) successfully validated this approach for real-time monitoring of apoptosis in leukemic cells during doxorubicin treatment.^174,175^ Point-scanning Raman microscopy captures spectra one point at a time, creating detailed chemical maps but requiring longer acquisition times.^176^ This approach underlies many current bioimaging methods, where each measurement becomes a pixel in the image, enabling spatial mapping of features such as relative intensities or principal components across the sample, aiming to decipher not only the biomolecular content but also its morphology. Line-scanning Raman microscopy illuminates a line instead of a point, speeding up data collection while maintaining a reasonable balance between spatial resolution and speed.^177–180^ Wide-field illumination excites a large area simultaneously for rapid data acquisition, though with lower spatial resolution and higher background interference.^181^ This approach has also been demonstrated in CARS microscopy^182,183^ but never in SRS modality due to the lack of commercially available bidimensional arrays of detectors each coupled to an independent lock-in amplifier; although it holds great promise for accelerating image acquisition, it remains under development, with no commercial systems currently available on the market. Alternatively, Ardini *et al.* (2023) proposed a wide-field spontaneous Raman microscope that employs a time-domain Fourier-transform approach, enabling the simultaneous acquisition of Raman spectra from all pixels of a 2D detector. This system features a birefringent interferometer with high delay stability, allowing for precise and rapid Raman mapping (30 minutes for 250 000 pixels, with less than 1 µm spatial resolution and around 23 cm^−1^ spectral resolution).^184^

### 3.3 Guidelines for choosing the best Raman approach for specific case studies

The selection criteria for the Raman-based approach are case study-oriented, as each technique presents its own advantages and disadvantages depending on the application and on the nature of the investigation. Some applications may require an unbiased, comprehensive analysis of the sample's overall chemical composition, while others may focus on spatial mapping of a predefined set of molecules corresponding to specific Raman shifts. For the former task, namely uninformed spectral analysis, SR is the most used choice in the literature (Fig. 3a), as it is highly informative, widely available on the market, offers high chemical specificity, and can be calibrated by using a large amount of commercially available materials.^185^ These advantages have contributed to the emergence of a new trend called “Spectralomics”, given the large number of metabolites analyzed along the entire spectral range.^186^ Additionally, its easy configuration and relatively low cost have facilitated its integration into portable devices or endoscopic fibers, allowing for *in vivo* analysis or intra-operative use.^12^ However, it may suffer from (1) time-consuming measurements for high-resolution bioimaging^187^ and (2) fluorescence interference from metabolites or optical substrates that absorb at the incident laser wavelength.

For the detection of specific analytes at relatively low concentrations, label-free SERS can be recommended.^188–190^ This technique offers better sensitivity compared to SR, but achieving high chemical specificity is complex due to its dependence on the substrate-sample interactions. SERS typically requires the use of nanoparticles,^191^ but recent studies have developed nanostructured substrates that enable label-free therapy monitoring,^192^ which is predominantly applied in diagnostics. Circulating tumor cells (CTC) enumeration has already reached clinical implementation through FDA-cleared non-Raman platforms, whereas SERS-based CTC detection remains largely investigational and requires further validation before routine clinical use.^193,194^ However, the reproducibility of SERS measurements has historically represented a challenge, mainly due to the strong dependence of the enhancement process on the physicochemical properties of the plasmonic substrate, nanoparticle morphology, and analyte–surface interactions.^195^ Substantial progress has been achieved through the development of more controlled fabrication strategies, improved surface functionalization approaches, and standardized protocols aimed at enhancing signal stability and measurement reproducibility.^196–198^ Alternatively, SESOR(R)S can be used as Raman-based tools to identify (and image) targeted tumors at depth through tissue, by using SER(R)S-active nanoparticles as efficient Raman-active labels.^172,199^

Coherent Raman techniques, such as SRS and CARS, are primarily employed for ultrafast imaging. Recently, the group of W. Min demonstrated the possibility of quantifying metabolites by measuring the cross sections of specific metabolites.^200–204^ In terms of sensitivity, they investigated the detection limits of both the SR and SRS approaches, presenting their findings through a two-dimensional detectability diagram (Fig. 3b).^204^ This diagram, based on the intrinsic Raman cross-section of the target molecule, acquisition time per pixel, sample concentration, and number of incident photons, illustrates the trade-off between SR and SRS sensitivity regimes. While SR can provide high sensitivity for low-concentration analytes when acquisition time is not limiting, SRS offers advantages for rapid imaging due to its higher acquisition speed and signal generation efficiency. However, for chemical imaging applications where short pixel dwell times are crucial to generating high-resolution spatially resolved images, SRS can be considered a very convenient approach.^203,204^ The high-wavenumber CH-stretching region (≈2800–3050 cm^−1^) is frequently selected for fast SRS imaging due to the strong lipid and protein signals and the reduced spectral complexity, whereas the fingerprint region provides richer biochemical information at the cost of increased acquisition and data analysis complexity. On the other hand, CARS approaches are more advanced for broadband coherent Raman implementations and may offer a valuable compromise between acquisition speed and spectral information content, since they can provide access to a broad vibrational range in a single acquisition (Fig. 3c). However, broadband CARS spectra are intrinsically affected by the non-resonant background, meaning that their spectral profiles do not directly correspond to spontaneous Raman spectra unless appropriate phase retrieval or background-removal procedures are applied. Therefore, while broadband CARS is highly promising for fast, chemically rich imaging, the quantitative and biochemical interpretation of its spectra still requires careful correction and validation (Fig. 3c).^4,151^ In recent years, coherent Raman systems have become increasingly available commercially. Examples include the STELLARIS 8 CRS system (Leica Microsystems, Wetzlar, Germany), the CORAL Raman imaging system (Cambridge Raman Imaging Ltd, Cambridge, UK), the Aquila system (Refined Laser Systems GmbH, Germany), and the NIO Laser Imaging System (Invenio Imaging, Santa Clara, USA), which uses stimulated Raman histology for rapid, label-free tissue imaging. Some of these systems are already on the market, while others are in the process of being released.

In practical terms, the choice of Raman modality should be driven by the biological and clinical question rather than by instrumental availability alone. SR spectroscopy remains preferable when broad, chemically faithful spectra are required; SERS is most useful for low-concentration analytes and biofluids, if substrate reproducibility is controlled; and SRS or CARS are most appropriate when imaging speed and spatial mapping are central, although broadband implementations require careful consideration of spectral fidelity, system complexity, and data interpretation.

### 3.4 Multimodal approaches

Label-free optical imaging systems rely on the inherent biochemical contrast of the cancer cell or tissue under investigation. Several contrast mechanisms can be leveraged at once to achieve more comprehensive chemical and/or morphological characterization by combining different optical imaging techniques into one analytical platform. On top of this, no specific sample preparation is required for label-free techniques, which makes it feasible to investigate the same pristine sample with diverse strategies at once. Due to the convenience of the higher-content information delivered, and the flexibility of optical systems in accommodating different label-free modalities often sharing the same laser source, multimodal label-free optical imaging has gained substantial traction in cancer research and therapy monitoring, with a few commercial platforms available today.^205^

SR and CARS were integrated into one single platform^206^ by Slipchenko *et al.* to study lipid bodies of cultured cells and live animals. The same group proposed a comprehensive platform featuring SR, CARS, and SRS for the pharmaceutical industry.^207^ A tandem Raman microscopy (TRAM) system including SRS, CARS and spontaneous (resonance) Raman scattering was also recently reported by the group of M. Baranska, for the study of non-adherent leukocytes.^208^ Even within the realm of coherent Raman, coupling different techniques proved beneficial: the redundancy of multimodal CARS and SRS has been exploited to reveal the early onset of therapy-induced senescence in cancer cells.^5^ More recently, the multimodal combination of SR and Brillouin spectroscopy was also introduced. Brillouin scattering is a signature of the stiffness and viscosity of the biological material,^209,210^ delivering valuable information about cell and tissue biomechanics,^211,212^ while Raman scattering supplements it with co-localized molecular insights.^213–215^

Although one disadvantage of coherent Raman techniques is the need for expensive ultrashort pulsed lasers and complex optical alignment, these illumination conditions conveniently engage with other attractive contrast mechanisms, such as two-photon excitation fluorescence (TPEF), three-photon excitation fluorescence (3PEF), second-harmonic generation (SHG) and third-harmonic generation (THG), which can deliver complementary, co-registered metabolic and structural insights in the same microscopy platform.^216–218^ Endogenous TPEF is generated by mitochondrial co-enzymes (*i.e.*, nicotinamide adenine dinucleotide (NAD(P)H) and flavin adenine dinucleotide (FAD)), whose ratio, often referred to as the redox ratio, indicates the metabolic activity of cells. This is extremely valuable to determine the metabolism of cancer throughout therapy and evaluate therapeutic outcomes.^219^ 3PEF enables imaging at greater depths,^220^ valuable for intravital deep-tumor imaging, and can excite other endogenous fluorophores such as tryptophan, serotonin, and lipofuscin, involved in key biochemical pathways and structural components.^221^ SHG, generated by collagen fibers that are acknowledged to have a significant impact on tumor progression and the TME, provides a picture of collagen density and spatial arrangement. THG is generated by refractive index heterogeneities in cells and tissues, giving insight about boundaries between different subcellular organelles or tissue areas. The group of S. Boppart was one of the pioneers of highly multimodal microscopy of tumors, merging CARS, SRS, TPEF, 3PEF, SHG and THG into a single optical microscope.^222^ They successfully identified carcinogenesis biomarkers thanks to the co-registered quantitative, chemical, functional, and structural *in situ* longitudinal analysis of mammary cancer tumor microenvironment. Similarly, the value of co-localized molecular and metabolic information provided by a multimodal CARS, SRS and TPEF microscope has been demonstrated to determine therapy effectiveness in human hepatic cancer cells within days after treatment.^223^

The exploitation of refractive index as a contrast mechanism is also at the basis of quantitative phase imaging (QPI), which provides refractive-index maps of cells and tissues with exquisite spatial resolution (*i.e.*, ∼150 nm lateral resolution) and can be beneficially complemented with Raman imaging chemical insights.^224,225^ J. W. Kang *et al.* introduced the first confocal SR and two-dimensional QPI system in 2011, for the co-registered morpho-molecular study of healthy and diseased blood samples, followed by other similar works targeting the phenotyping of breast cancer cells^226^ and acute lymphoblastic leukemia subtypes.^227^ Being much faster, QPI has been suggested as a prescreening tool to be followed by Raman spectroscopy on designated targets to conclude cell and tissue characterization.

Optical coherence tomography (OCT) has also been coupled with SR.^228,229^ In fact, OCT is ideal for thick tissue and depth-sectioning requirements, often associated with *ex vivo* or *in vivo* translational and (pre)clinical applications, and similarly to the multimodal scenario of QPI and Raman, the latter is fundamental to add chemical information. In 2017, the group of B. Roth presented a combined OCT-Raman probe validated for the *in vivo* morpho-molecular analysis of human skin.^230^ More recently, Kukk *et al.* combined OCT with both Raman spectroscopy and ultrasound tomography for non-invasive skin cancer diagnostics and lesion depth measurement.^229^

## 4 Raman spectral features associated with cancer therapy response

### 4.1 Raman shifts for monitoring the tumor microenvironment during treatment

The correlation of Raman bands with their vibrational assignments is essential for identifying the biomolecules defining the biological material, from real-time tumor assessment during surgery to studies using controlled experimental models under treatment conditions. In this section, we detail the most important spectral assignments reported in literature for the key biomolecules present in the tumor and the TME, and related to therapy response monitoring and prediction studies (Fig. 4). In addition, peak assignments enabling identification of cancer cells from the surrounding healthy tissue are also reported, considering their potential use to track tumoral progression during therapy. However, while this section summarizes specific spectral assignments frequently reported in the literature, individual Raman bands in complex biological mixtures are rarely specific to a single molecular species. For example, C=C stretching or CH_2_ deformation bands can arise from multiple lipids or proteins rather than from a single metabolite. These spectral features should therefore be regarded as indicators of broader biochemical changes rather than unique molecular identifiers. For quantitative analysis, single-band assignments are generally insufficient, and complete spectral fingerprints combined with multivariate analysis or spectral unmixing are preferable.

**Fig. 4:**
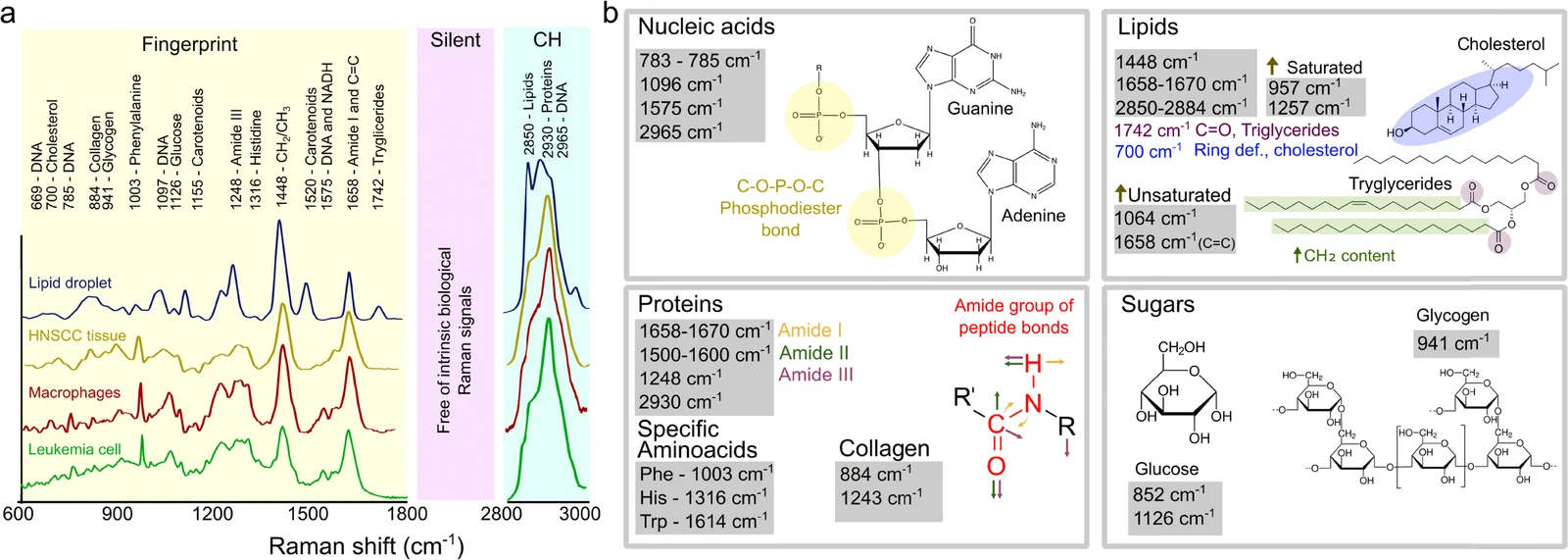
Main Raman shifts associated with the tumor and its microenvironment to monitor therapy response. (a) Representative Raman spectra of the tumor microenvironment: lipid droplet (blue), tumor tissue from head and neck cancer (yellow), isolated immune cells (macrophage, red), and isolated leukemia cells (green). The spectra highlight key peak assignments, with Raman shifts corresponding to main biomolecules. (b) Key biomolecular structures and their associated Raman bands, consistent with the assignments summarized in Table 2.

In clinical spectroscopy, Raman spectra are typically divided into three main regions: the fingerprint region (400–1800 cm^−1^), the silent region (1800–2800 cm^−1^), and the CH region (2800–3200 cm^−1^), as shown in Fig. 4a. The silent region lacks endogenous signals from biomolecules. In Table 2, we summarize peak assignments through the fingerprint and CH regions that constitute the relevant Raman vibrational range for biological and biomedical studies. From a general perspective, the group of M. Baranska published two comprehensive reviews in 2013 and 2014 detailing the application of Raman spectroscopy for the analysis of lipids and proteins, providing an extensive overview of spectral assignments, signal interpretation, and the potential of Raman techniques for studying biomolecular composition in complex biological samples.^231,232^ More recently, in 2025, Nowakowska *et al.* described the Raman map of the human cell, assigning the Raman bands of subcellular structures.^233^ Additional Raman spectral libraries of key biomolecules and metabolites have been developed to aid in the interpretation of complex biological spectra.^234–236^

**Table 2:** Raman spectral features of the tumor microenvironment and their relevance to cancer therapy response studies. Nomenclature: ↑ indicates an increase or a high signal intensity, while ↓ denotes a decrease or a low signal intensity

Spectral region	Wavenumber (cm^−1^)	Biomolecule	Vibrational mode	Relevance to tumor and tumor microenvironment dynamics	Ref.
Fingerprint	505	Proteins	Disulphide bonds	↑ in colorectal cancer patients measured in dried plasma	237
	622	Phenylalanine	C–C twisting mode	↑ in prostate cancer cells compared to lymphocytes from healthy donors	279
	669	Thymine and guanine	Ring breathing	↓ in lung cancer cells exposed to actinomycin D	240
	700	Cholesterol	Ring deformation	Lipid droplets detection in X-ray treated prostate cancer cells	241
	722	Phosphatidylcholine	C–C–N+ symmetric stretching	↓ in colorectal cancer cells treated with oxaliplatin	244
	750	Cytochrome *C*	Pyrrole breathing mode ν15	↓ in HeLa cells exposed to actinomycin D	242
	758–760	Tryptophan	Symmetric indole ring-breathing	↑ in colorectal cancer measured in dried plasma compared to healthy donors	237
	783–785	DNA	C′–O–P–O–C′3 phosphodiester	↓ in 4T1 cells after cisplatin treatment//↓ due to DNA–platinum adducts in HCT116 after oxaliplatin treatment	242 and 244
	811	RNA	C′–O–P–O–C′3 phosphodiester	↓ due to DNA–platinum adducts in HCT116 after oxaliplatin treatment	244
	826	Nucleic acids	O–P–O backbone	↑ in colorectal cancer measured in dried plasma compared to healthy donors	237
	829 & 845	Tyrosine	Ring breathing	↓ in tyrosine phosphorylation in aggressive cancers (measured *via* 1586/829 ratio)	245
	851–855	Keratin/tyrosine	Ring breathing/C–C stretch	Keratin monitoring in skin cancer//↓ in invasive SCC compared to healthy tongue tissue	246 and 269
	852	Sugars (glucose, glycerol)	C–O–C skeletal stretching	↑ in serum of locally advanced rectal cancer with poor prognosis to chemoradiation treatment	248
	878	Glutamic acid, mannitol or arginine	COO^−^ deformation	Biomarker enabling tumor discrimination	247
	884	Collagen/proline	C–C stretching vibrations of proline	↑ in radiation-resistant UM-SCC-47 tumors at 24 h following radiation compared to non-radiated samples	12
	933–937	Collagen	*v*(C–C) skeletal of collagen backbone	↑ in radiation-resistant UM-SCC-47 tumors at 24 h following radiation compared to non-radiated samples	12
	941	Glycogen	C–C stretch backbone	↓ in high-grade cancerous urothelial cells//Present in glioma biopsy	280
	957	Saturated fatty acids	C–H bending	↓ in serum of locally advanced rectal cancer after chemotherapy//↓ in serum of pancreatic ductal adenocarcinoma patients compared to healthy patients	248
	1003–1006	Phenylalanine	Phenyl ring bending	↑ in colon cancer cells	249
	1031–1032	Phenylalanine	C–H in-plane bending	↑ in DU145 cells (prostate cancer cell line)	259 and 281
	1064–1066	Lipids	C–C stretching	↑ in unsaturated fatty acids	231 and 234
	1097	Nucleic acid	PO_2_^−^ backbone stretch	↓ in DAPT-treated osteosarcoma cells, linked to impaired DNA replication	250 and 251
	1126	Glucose	C–C stretching, C–O stretching, C–O–H in-plane bending	↑ in serum of pancreatic ductal adenocarcinoma patients compared to healthy patients	248
	1155	Carotenoids	C–C stretching vibrations in the polyene chain	↓ in liquid biopsy of colorectal cancer compared to healthy	234 and 237
	1157	Proteins	C–C/C–N stretching	↑ in breast malignant tissue compared to normal and fat tissue	257
	1208	Phenylalanine, tryptophan	NH_3_^+^ asymmetric rocking	↑ in doxorubicin-treated lung cells showing DNA repair	258
	1248	β-sheet protein, Amide III	C–N stretching and N–H bending	↑ in lung cancer tissue compared to healthy controls, further increase observed after microwave ablation treatment	260
	1257–1268	Long-chain saturated lipids	CH_3_/CH_2_ twisting and wagging	↑ in chemoradiation therapy in locally advanced rectal cancer (LARC) patient sera	248
	1300	Lipids	CH_2_ scissoring and CH_3_ twisting	Signal associated to long-chain fatty acids (phospholipids)	231 and 262
	1316	Histidine	Asymmetric rocking and C–H stretching	↓ due to DNA–platinum adducts in HCT116 after oxaliplatin treatment	244
	1420	Plasma lipids	Scissoring and twisting vibrations of the CH_2_ and CH_3_ groups	↑ in liquid biopsy of colorectal cancer compared to healthy	231 and 237
	1448	Lipids and proteins	CH_2_ and CH_3_ scissoring and deformation	↑ in STS-treated tumor spheroids//↑ malignant breast tissue	266 and 267
	1520–1526	Carotenoids	Conjugated C=C stretch	↓ in liquid biopsy of colorectal cancer compared to healthy//↑ in acute myeloid leukemia (AML) M5a cells	237 and 254
	1575	DNA and NADH	Ring breathing modes	↓ with oxaliplatin treatment in HCT116 cells	244
	1586	Phosphorylated tyrosine	—	↑ in U-87 MG aggressive glioblastoma cell lines	245
	1614	Tryptophan	—	↑ in invasive SCC compared to healthy tongue tissue	269
	1658	Lipids and proteins (α-helix)	Amide I and C=C stretching	Differentiate breast cancer cells: ↑ in MCF-10A and MDA-MB-436 compared to MCF-7 cells	270
	1670	β-sheet proteins	Amide I	Differentiate breast cancer cells: ↑ in MCF-10A and MDA-MB-436 compared to MCF-7 cells	270
	1742	Triglycerides	C=O stretching (ester group)	↑ in lipid droplets found in dysregulated lipid metabolism of aggressive prostate cancer	272

CH region	2850	Lipids	Symmetric stretching vibration of C–H (CH_2_ groups)	↑ in lipid droplets found in senescent cells	5
	2884	Lipids	Asymmetric stretching vibration of C–H (CH_2_ groups)	↑ in lipid droplets found in senescent cells	5
	2930	Proteins	C–H stretching (CH_3_ and CH_2_)	Allow rapid imaging of tumor slices to guide surgical margin assessment	13
	2965–2975	DNA	Asymmetric C–H stretch of the CH_3_ group of thymine	Allow label-free DNA imaging *in vivo* with SRS	276

Raman spectral signals in the lower fingerprint range contain the band at 505 cm^−1^, related to disulfide bridges of proteins, a recently proposed biomarker of colorectal cancer measured on dried plasma – a promising approach for liquid biopsies.^237^ The band at 622 cm^−1^ is attributed to the C–C twisting mode of phenylalanine, a key amino acid in proteins.^238,239^ The peak at 669 cm^−1^ corresponds to thymine and guanine (DNA, nucleic acid bases), used to monitor the DNA response of lung cancer cells to actinomycin D treatment.^240^ Cholesterol, an essential lipid component, exhibits a distinctive peak at 700 cm^−1^, associated with ring deformation and used to detect lipid droplets in prostate cancer.^241^ The signal at 722 cm^−1^ is assigned to the C–N symmetric stretching of phosphatidylcholine, marking lipid membranes; this Raman shift features a decrease in colorectal cancer cells treated with oxaliplatin.^242–244^ Tryptophan, a significant aromatic amino acid monitored in the identification of glioblastoma (GBM) microinfiltration,^243^ shows a peak at 758 cm^−1^ due to symmetric indole ring breathing, while cytosine and uracil, both nucleobases of DNA and RNA, produce a signal at 783–785 cm^−1^ corresponding to pyrimidine ring breathing.^225^ For example, Cutshaw *et al.* (2023) reported a decrease of the 783 cm^−1^ band after oxaliplatin treatment in colorectal cancer,^244^ while Kothadiya *et al.* (2024) linked DNA spectral signals to cisplatin prognosis in 4T1 cells.^242^

The band at 829 cm^−1^, linked to out-of-plane ring breathing of tyrosine, has emerged as a Raman biomarker to determine phosphorylation status in aggressive cancers.^245^ The peak at 855 cm^−1^ corresponds to the ring breathing mode of tyrosine and C–C stretching mode of proline ring and was used to monitor skin cancer status *via* keratin measurements in epidermal extracellular matrix (ECM).^246^ Notably, the COO^−^ deformation mode of glutamic acid is represented at 878 cm^−1^, a feature that Woods *et al.* (2022) identified as a potential biomarker for early patient screening: in their work, they proposed that cancer-related alterations could be detected by establishing lower and upper threshold values, which vary depending on the cancer type.^247^ Bands at 884 and 933 cm^−1^ are associated to collagen that is overexpressed in tumor radiation resistance models.^12^ Citric and succinic acids exhibit a Raman peak at 938 cm^−1^, attributed to O–H⋯O out-of-plane vibrations of hydrogen bonds. This band decreases in pancreatic ductal adenocarcinoma (PDAC) due to citrate consumption through the Krebs cycle, stimulating *de novo* fatty acid synthesis. Recently, Cutshaw *et al.* (2025) exploited this spectral feature in patient sera to monitor chemotherapy response, showing that alterations in citrate/succinate, glutamate, and lipid profiles reveal ongoing metabolic reprogramming in tumors with ineffective neoadjuvant chemoradiation therapy.^248^

The spectral region between 1000 and 1800 cm^−1^ contains some of the most informative Raman bands. The sharp band appearing at 1003 cm^−1^ is linked to C–C symmetric stretching and symmetric ring in-plane bending of phenylalanine. Similarly, the band at 1031 cm^−1^ is assigned to ring in-plane bending and C–H in-plane bending of the phenylalanine residue, providing further structural information. These two bands, especially the one at 1003 cm^−1^, are widely used in Raman studies, since this aromatic amino acid may act as a precursor of mutagenic, genotoxic, and carcinogenic compounds, and, for example, its overexpression in colon tissue could indicate early colon cancer.^249^ The peak at 1064 cm^−1^ corresponds to C–C stretching in lipids, *e.g.* (mainly unsaturated) fatty acids, showing very strong signals in molecules like palmitic acid.^231,234^ The DNA peak at 1097 cm^−1^, attributed to the PO_2_^−^ backbone stretch, has been reported by Li *et al.* (2020) to decrease in osteosarcoma cells treated with DAPT (a γ-secretase inhibitor), consistent with impaired DNA replication due to base-pair disruption,^250^ and in line with Wen *et al.* (2020), who observed a similar decrease in breast cancer cells treated with Trametinib, associated with cytotoxicity, apoptosis, and reduced DNA replication.^251^ This DNA peak may be particularly relevant in studies on fixed tissue or cells on quartz slides, where background signals around 800 cm^−1^ could obscure detection of the sharper, more intense DNA band at 783 cm^−1^.

Carotenoids, natural dietary antioxidants, are a particularly appealing target for Raman studies as they exhibit prominent Raman spectral features, including intense bands around 1155 cm^−1^ and 1526 cm^−1^, which are frequently used for carotenoid detection.^252^ These bands are widely used as biomarkers since carotenoids are typically more abundant in healthy tissues than in malignant ones, enabling discrimination between normal and cancerous regions, as demonstrated, for instance, in dried blood plasma from colorectal cancer patients, in breast tissue biopsies and leukemia cells.^237,245,253,254^ The monitoring of the –C=C– bond is particularly relevant for β-carotene treatment and its impact on brain tumor metabolism, as recently demonstrated by Jarczewska *et al.* (2025).^255^ Carotenoids detection becomes particularly straightforward in Raman spectroscopy when the incident laser wavelength falls in the visible range (*e.g.*, 532 nm, 633 nm, or 660 nm), where these molecules strongly absorb light, leading to pre-resonance or resonance Raman enhancement. This exemplifies how Raman spectroscopy can uniquely detect specific molecular species that are otherwise difficult to identify using standard omics approaches; indeed, carotenoids lack dedicated staining or immunolabeling methods and can be revealed only through complex isotope-based labeling or destructive analytical techniques such as HPLC, therefore remaining inaccessible to routine diagnostic assays.^256^

Close to carotenoid signals, proteins exhibit a band at 1157 cm^−1^ due to C–C/C–N stretching, used to discriminate in breast cancer *ex vivo* samples,^257^ while amino acids like phenylalanine, tryptophan and tyrosine exhibit NH_3_^+^ asymmetric rocking at 1208 cm^−1^, whose increase indicates an influx of anti-apoptotic proteins attempting to repair DNA and prevent apoptosis in lung cells after long-term doxorubicin treatment.^258^ For amide III, Stone *et al.* (2004) differentiated features according to protein secondary structure. Specifically, bands at 1220–1221 cm^−1^ were assigned to β-sheet conformations; a feature at 1243 cm^−1^ was attributed to collagen (CH_2_ wagging and C–N stretching) with possible contributions from pyrimidine bases (C, T); the 1240–1265 cm^−1^ region was assigned to the amide III mode (C–N stretching of proteins), indicating predominantly α-helix conformation; and a band at 1279 cm^−1^ was also associated with α-helical structures.^259^ In lung cancer tissue, the band at 1248 cm^−1^ shows increased intensity compared to healthy controls, with a further robust increase observed after microwave ablation treatment.^260^ Other works, such as Wang *et al.* (2022), also associated this Raman shift with guanine and cytosine, observing that it decreased in inverse proportion to the cisplatin dose in K7M2 mouse osteosarcoma cells.^261^

Lipids present a peak at 1300–1304 cm^−1^, arising from CH_2_ scissoring and CH_3_ twisting vibrations, commonly associated with long-chain fatty acids and, in some cases, phospholipids.^231,262^ This band can be widely used to monitor the tumor response, since lipids are essential for tumor growth and dissemination.^263^ Altered lipid content in the TME can further influence its aggressiveness, while contributing to resistance against chemotherapy and immunotherapy.^264,265^ In the case of proteins and amino acids, Histidine exhibits NH_3_^+^ asymmetric rocking and C–H stretching vibrations at 1316 cm^−1^, whose intensity was reported to decrease by Cutshaw *et al.* (2023) in association with cytotoxic oxaliplatin-induced DNA–platinum adduct formation.^244^ This observation aligns with the findings of Kothadiya *et al.* (2024), who attributed similar changes to cisplatin-induced metabolic responses in cancer cells.^242^ The signal at 1448 cm^−1^, one of the most significant bands in Raman spectra, corresponds to CH_2_ and CH_3_ scissoring and deformation, typically found in both lipids and proteins (Fig. 4b). This signal is overexpressed in malignant breast tissue, and has been used for rapid evaluation of surgical margins.^266^ Additionally, in monitoring the response of multicellular tumor spheroids (MTS) to Staurosporine (STS), a protein kinase inhibitor and potent pro-apoptotic agent, the Raman band at 1448 cm^−1^ showed increased intensity in STS-treated samples.^267^

In contrast, the band at 1575 cm^−1^, associated with DNA and NADH ring-breathing modes, shows a marked decrease in HCT116 cells following oxaliplatin treatment, consistent with the drug's cytotoxic mechanism of action, which involves cell-cycle arrest and inhibition of DNA replication.^244^ This Raman band lies close to the characteristic signal of phosphorylated tyrosine at ∼1586 cm^−1^, where the 1586/829 intensity ratio has been reported to correlate with the aggressiveness of U-87 MG glioblastoma cell lines.^245^ Such a spectral ratio could potentially serve as a biomarker to track the protein tyrosine kinase inhibitor resistance in malignant tumors,^268^ by enabling label-free monitoring of phosphorylation dynamics. Another relevant metabolite, namely l-tryptophan, exhibits three principal Raman bands at 1562, 1584, and 1614 cm^−1^, whose intensities markedly increase during the *in situ* transformation of healthy tissue into squamous cell carcinoma in head and neck cancer.^269^

The upper part of the fingerprint region (1650–1800 cm^−1^) comprises characteristic signals of proteins and lipids often superimposed. In this range, the amide I band of proteins with α-helix conformation and the C=C stretching mode of lipids typically appear around 1658 cm^−1^, while the amide I band associated with β-sheet protein structures is observed near 1670 cm^−1^.^232^ Talari *et al.* (2015) used these spectral features to distinguish breast cancer cell lines *via* principal component analysis (PCA), showing that protein-related bands (amide I, β-sheet structures, and aromatic residues such as phenylalanine, tyrosine, and tryptophan) in the range of 1548–1687 cm^−1^ were more intense in MCF-10A and MDA-MB-436 cells, whereas nucleic acid signals at 1576 cm^−1^ predominated in MCF-7 cells.^270^ Lastly, the presence of triacylglycerols (triglycerides) or fatty acids can be identified by the C=O stretching vibration of the ester functional group, typically detected around 1742–1750 cm^−1^.^231^ The relevance of this band stems from the growing interest in dysregulated lipid metabolism in cancer, which is associated with rapid cell proliferation and tumor progression.^271^ In particular, triglycerides and lipid-rich molecules, such as cholesteryl ester (CE)-rich lipid droplets (LDs), have been imaged using SRS, revealing their accumulation in aggressive prostate cancers.^272^ Additionally, El-Mashtoly *et al.* (2015) proposed that this spectral region can be used to monitor the effects of ligand or drug binding in epidermal growth factor receptor (EGFR)-expressing cell lines, specifically to determine lipid droplets.^273^

Considering the CH region, the band at 2840–2850 cm^−1^ corresponds to the symmetric stretching vibration of CH_2_ groups, which is commonly found in lipids. This feature is widely used for lipid droplet imaging; for example, Bresci *et al.* (2023) visualized lipid droplet accumulation in senescent cells using SRS.^5^ Additionally, the peak at 2930 cm^−1^ corresponds to CH stretching vibrations in CH_2_ and CH_3_ groups, predominantly arising from proteins and enabling clear discrimination between lipid-rich and protein-rich regions within a sample. This is why the CH region is extensively used in Raman-based digital pathology: both lipids and proteins contain a high density of CH_2_ or CH_3_ groups, exhibit strong Raman cross-sections with excellent signal-to-noise ratios, and present well-defined and distinct Raman shifts that facilitate rapid, label-free biochemical imaging.^13,134,274^ These conditions enable both imaging and quantitative analysis, owing to the intrinsic linear correlation between molecular concentration and Raman signal intensity in SRS.^275^ Finally, DNA produces a significant peak at 2967–2973 cm^−1^ due to the asymmetric C–H stretch of the CH_3_ group of thymine.^276,277^ A representative example of the use of the CH region to evaluate cancer therapy response was reported by Tipping *et al.* (2022), who employed SRS imaging to assess breast cancer cell responses to statin treatment. By analyzing CH_2_ and CH_3_ vibrational bands (2851–2930 cm^−1^) and their ratiometric contrast, they revealed lipid accumulation patterns associated with differential drug sensitivity and potential resistance mechanisms (see Section 7.1 for more detailed information about this work).^278^ In summary, the CH region provides strong Raman signals that allow the creation of three-channel images distinguishing DNA, proteins, and lipids, making it highly useful for general cellular mapping. However, it lacks the chemical specificity required to probe individual metabolites, and therefore, more detailed biochemical information must be obtained from the fingerprint region.

After monitoring the tumor microenvironment during a specific treatment, a natural question arises: what about the localization, uptake, evolution, and molecular structure of therapeutic agents during therapy? While not all treatment modalities can be directly tracked (*e.g.*, radiotherapy), the growing development of nanoparticle-based systems, drug-delivery platforms, targeted therapies, and chemotherapeutic agents enables such investigations. In the following section, we provide, to the best of our knowledge, one of the first comprehensive overviews of Raman spectral shifts used to monitor anticancer drugs, either in their native form or *via* Raman tags, offering a framework for label-free tracking of therapeutic compounds during treatment response monitoring.

### 4.2 Raman shifts to label-free tracking of anticancer drugs

Several anticancer drugs and their delivery systems can be directly monitored by Raman spectroscopy, as many pharmacologically active compounds possess rigid aromatic scaffolds and functional groups that are uncommon in endogenous biomolecules.^7,20^ As a result, these molecules often exhibit intense and highly distinctive Raman spectra.^282^ These spectral signatures enable label-free tracking of the drug's localization, uptake, and structural evolution during treatment (Fig. 5). Here, we review the main studies where chemotherapeutic or targeted agents have been detected and followed through their characteristic Raman bands (Table 3) to provide insight into drug biodistribution, metabolism, and mechanism of action at the tissue level. It should be noted that the detectability of a drug-specific Raman signature depends not only on the administered dose, but also on factors such as the intracellular accumulation, molecular environment, Raman cross-section, and the limit of detection achievable under the experimental conditions. Therefore, pharmacological concentrations used to induce a biological response should not be directly assumed to correspond to detectable Raman signals from the drug itself.

**Fig. 5:**
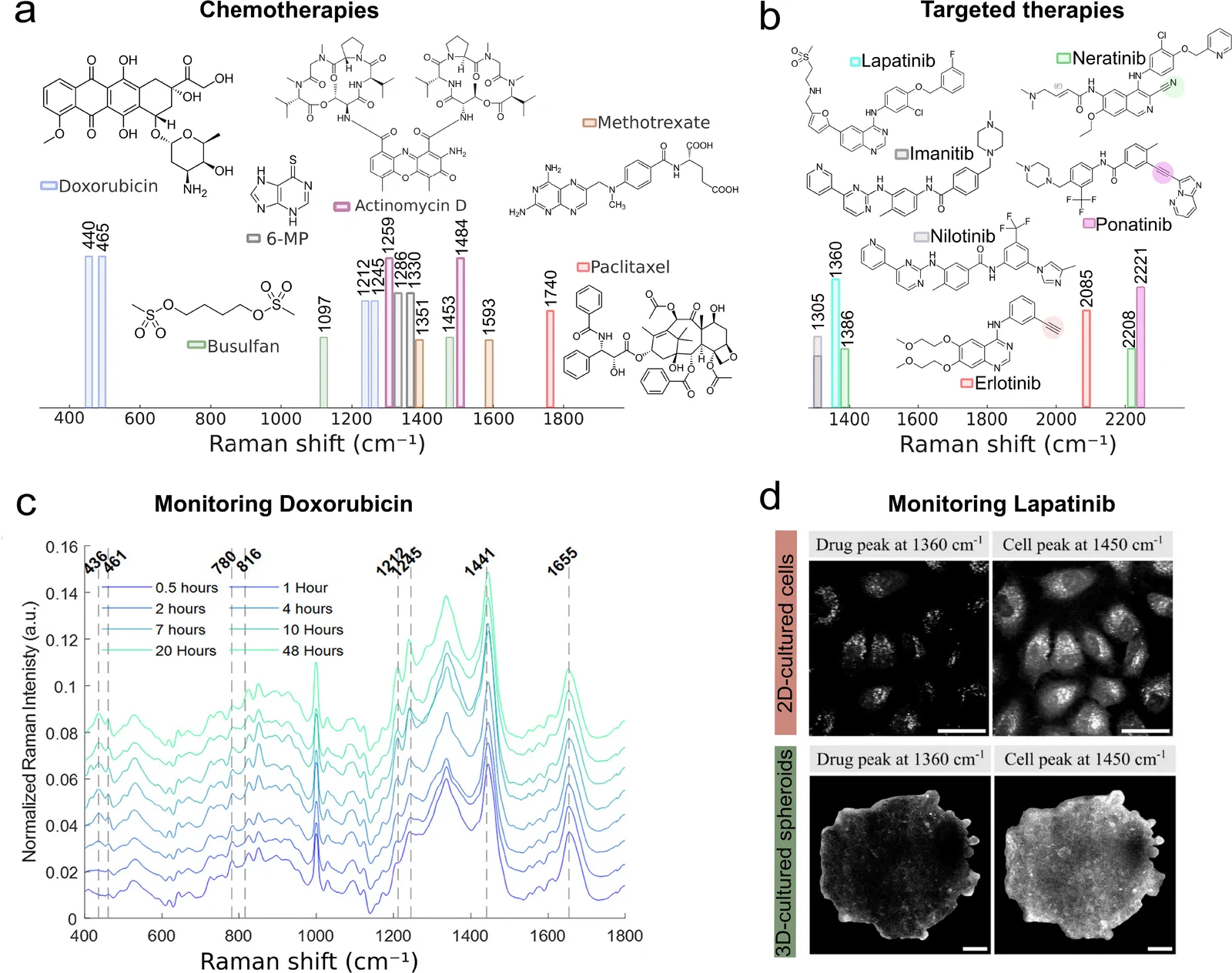
Application of Raman spectroscopy for the characterization and intracellular monitoring of anticancer drugs. (a) Chemical structures and corresponding characteristic Raman marker bands (wavenumbers in cm^−1^ for a selection of standard chemotherapeutic agents, including doxorubicin, cisplatin, and paclitaxel). The bar chart illustrates distinct spectral fingerprints used for drug identification. (b) Chemical structures and specific Raman marker bands for targeted therapies, such as Lapatinib, Imatinib, and Erlotinib. (c) Temporal monitoring of drug pharmacokinetics in A549 cells. The plot displays normalized Raman spectra tracking the intracellular accumulation and spectral evolution of Doxorubicin over a 48-hour incubation period. Characteristic drug peaks (*e.g.*, 436, 461, 1212, 1245 cm^−1^) increase in intensity relative to cellular background signals. Adapted from Perez-Guaita *et al.*^286^ with permission from Wiley. (d) Label-free spatial imaging of drug distribution. Raman microscopic images compare the localization of Lapatinib (drug peak at 1360 cm^−1^) against the total cellular biomass (cell peak at 1450 cm^−1^) in both 2D monolayer cultures and 3D tumor spheroids. Adapted from Xu *et al.*^287^ with permission from American Chemical Society.

**Table 3:** Raman spectral features of anticancer drugs and Raman tags to track their localization, uptake, evolution, and molecular structure during treatment

Drug name	Raman shift (cm^−1^)	Label-free Raman approach	Drug class/mechanism	Treatment type	Matrix	Ref.
Alkyne-functionalized triphenylphosphonium (TPP)	2216	SRS	Mitochondria-targeting agent	Targeted therapy/drug delivery	HeLa cells	300
Erlotinib	2085–2140 and 1006 (demethylated derivate)	Spontaneous	Inhibitor of the epidermal growth factor receptor (EGFR)	Targeted therapy	SW480 colon cancer cells	283
Imatinib	1592–1609, 1292–1307, 1040–1032, and 983–989	SERS	Tyrosine kinase inhibitor	Targeted therapy	Human plasma	284
Neratinib	1386 and 2208	Spontaneous	Binds to EGFR and HER2 receptors	Targeted therapy	HER2-positive breast cancer cells (SK-BR-3) NCI-H1975 and Calu-3	285
Ponatinib	2221	SRS	Tyrosine kinase inhibitor	Targeted therapy	KCL-22 (live human chronic myeloid leukemia) KCL	288
Imatinib	1305	SRS	Tyrosine kinase inhibitor	Targeted therapy	BaF3/BCR-ABL1 and Ba/F3/wt cells	289
Nilotinib	1305	SRS	Tyrosine kinase inhibitor	Targeted therapy	BaF3/BCR-ABL1 and Ba/F3/wt cells	289
Chloroquine	1370	SRS	Synergistic effect with imatinib	—	BaF3/BCR-ABL1 and Ba/F3/wt cells	289
Lapatinib	1360	SRS	Tyrosine kinase inhibitor	Targeted therapy	A549 cells and spheroids	287
6-mercaptopurine	1286 and 1330	SERS	Purine synthesis inhibition	Chemotherapy	A549 cells	294
Actinomycin D	1259 and 1484	Spontaneous (line illumination)	DNA intercalating agent	Chemotherapy	HeLa cells	295
Paclitaxel	1740	Spontaneous	Blocks mitosis	Chemotherapy	MCF-7 cells	296
Busulfan	1097 and 1453	Spontaneous	Alkylating agent	Chemotherapy	Human serum	297
Methotrexate	1351 and 1593	Spontaneous	Antimetabolite and folate antagonist	Chemotherapy	Human serum	297
Doxorubicin	431–440, 465, 1215 and 1245	Spontaneous	Anthracycline antibiotic (intercalates DNA)	Chemotherapy	A549 and Calu-1 lung cancer cells	258, 286 and 293

Erlotinib and the tyrosine kinase inhibitors Imatinib, Neratinib, Ponatinib and Lapatinib are examples of targeted therapies evaluated with Raman spectroscopy (Fig. 5b). Erlotinib, a clinically approved inhibitor of the epidermal growth factor receptor (EGFR) for the treatment of locally advanced or metastatic non-small cell lung cancer and pancreatic cancer, exhibits a strong signal at 2085 and 2140 cm^−1^ due to the C≡C stretching. El-Mashtoly *et al.* (2014) used this band to image the uptake in SW480 colon cancer cells, while identifying its metabolic conversion to a demethylated derivative with the Raman band at 1006 cm^−1^ corresponding to the trigonal ring deformation of the phenyl group, typical of mono-, 1,3-, and 1,3,5-substituted benzene derivatives.^283^ Imatinib, an oral tyrosine kinase inhibitor that targets pathways involved in angiogenesis and tumor proliferation, was evaluated by Fornasaro *et al.* (2018). Distinct bands at 1592–1609, 1292–1307, 1040–1032, and 983–989 cm^−1^, showing slight shifts between the powdered form and plasma-dissolved samples, were used for label-free drug quantification in human plasma during treatment.^284^ Aljakouch *et al.* (2018) investigated the metabolism of Neratinib, whose nitrile group (–C≡N) produces a strong Raman signal in the silent region (2208–2217 cm^−1^) and can be used to track drug uptake, intracellular distribution, and metabolism in HER2-positive breast cancer cells (SK-BR-3) and in non-small-cell lung cancer (NSCLC) cells with (NCI-H1975) and without (Calu-3) the T790M EGFR mutation. They found that most metabolites accumulated in lysosomes, both EGFR and HER2 underwent Neratinib-induced endocytosis, and novel metabolites were identified in breast cancer and NSCLC cells.^285^ Sepp *et al.* (2019) quantified drug uptake and lysosomal sequestration down to nanomolar concentrations of Ponatinib, already approved for the treatment of chronic myeloid leukemia (CML), in live human CML cell lines using stimulated Raman scattering (SRS) microscopy and exploiting the intrinsic alkyne vibration at 2221 cm^−1^ present in the drug's molecular structure.

Fu *et al.* (2014) quantitatively analyzed the intracellular distribution of imatinib and nilotinib, evaluating chloroquine as a potential synergistic agent, and compared them with the allosteric inhibitors GNF-2 and GNF-5.^289^ SR spectra of the five compounds were acquired to identify major peaks for SRS imaging: 1305 cm^−1^ (imatinib and nilotinib), 1370 cm^−1^ (chloroquine), and 1600 cm^−1^ (GNF-2 and GNF-5). Following treatment in BaF3/BCR-ABL1 cells, SRS microscopy revealed >1000-fold lysosomal enrichment of imatinib and nilotinib, with nilotinib's low aqueous solubility further enhancing accumulation. Co-treatment with chloroquine reduced imatinib lysosomal sequestration by over tenfold, likely due to chloroquine-induced lysosomal pH elevation, suggesting that chloroquine may potentiate tyrosine kinase inhibitor efficacy *via* both autophagy inhibition and lysosome-mediated drug–drug interactions. In contrast, SRS imaging of GNF-2 and GNF-5 showed no lysosomal accumulation, with spectra indicating moderate cytoplasmic enrichment without nuclear penetration. Lastly, Fu *et al.* (2023–2024) investigated the uptake of lapatinib in 2D and 3D A549 cells using SRS imaging, exploiting the 1360 cm^−1^ peak associated with bending and stretching of the furan ring. To quantify single-cell growth rates, 2D A549 cells were labeled with d-LIV (deuterated l-leucine-d10, l-isoleucine-d10, and l-valine-d8) for 6 h, yielding a strong C–D signal-to-noise ratio for reliable analysis. In spheroids, stronger drug signals appeared at the outer layers and weaker ones in the inner core. At 50–100 µM, the intracellular drug uptake suggested saturated lapatinib accumulation, while uptake levels were comparable between 2D monolayers and 3D spheroids, with no significant differences within each treatment concentration (Fig. 5d).^287,290^

Chemotherapies like platinum-based drugs, doxorubicin (DOX), 6-mercaptopurine, actinomycin D, paclitaxel, busulfan or methotrexate are examples of drugs that have been tracked during therapy simulation, both in biological samples like human plasma and experimental models (Fig. 5a). Torres *et al.* (2018) reported the Raman spectra of five clinically used platinum-based chemotherapeutics (cisplatin, carboplatin, nedaplatin, oxaliplatin, and heptaplatin) and showed that their Pt-N stretching modes provide distinct vibrational signatures for drug identification. The main Raman markers include bands at 1294 and 1312 cm^−1^ for cisplatin, 945 cm^−1^ for carboplatin, 1377 and 1692 cm^−1^ for oxaliplatin, 929 cm^−1^ for nedaplatin, and 610, 1402, and 1602 cm^−1^ for heptaplatin.^291^ Anthracycline tracking *in vitro* was extensively investigated by Szafraniec *et al.* (2016), who characterized the Raman spectra of DOX, daunorubicin (DNR), their epimers (EDOX, EDNR), and ten related analogs. Using both 488 nm and 785 nm incident laser wavelengths, they demonstrated that DOX predominantly localizes in the nuclear region of A549 cells after 24 h of exposure.^292^ In the specific case of doxorubicin, Farhane *et al.* monitored its intracellular uptake in Calu-1 and A549 cells by using the Raman bands at 440, 465, 1085, 1215, and 1245 cm^−1^ as distinctive spectral markers of the drug (Fig. 5c).^258,293^

Han *et al.* (2014) employed a label-free surface-enhanced Raman spectroscopy (SERS) approach using Au@Ag core–shell nanoparticles to monitor and visualize the intracellular metabolism of the antitumor drug 6-mercaptopurine (6-MP), which is widely used in the chemotherapy of human acute lymphoblastic leukemia. In A549 cells, the metabolic changes of 6-MP were tracked through the strong enhancement of its 1276–1286 cm^−1^ and 1330–1334 cm^−1^ peaks, corresponding to vibrations of the purine ring and adjacent C–N and C–C bonds.^294^ Li *et al.* (2022) simultaneously studied drug uptake and cellular response of HeLa cells to actinomycin D (ActD), an antibiotic with anticancer activity.^295^ They evaluated the ActD-DNA interactions in HeLa cells and, after a gradient from 0 to 10 µM treatment, detected characteristic Raman peaks at 1259 and 1484 cm^−1^, corresponding to the C–C=C and C–C=N stretching vibrations in the phenoxazone chromophore of ActD, which allowed mapping its distribution in the nucleus. Surprisingly, at lower ActD concentrations, autofluorescence signals in the 1950–2250 cm^−1^ range were used by the authors to visualize the intracellular distribution of ActD, likely reflecting DNA-bound drug. Salehi *et al.* (2013) monitored paclitaxel, a pro-apoptotic anticancer drug, in MCF-7 cells after uptake using the Raman band at 1740 cm^−1^ (C=O stretching), as the 1002 cm^−1^ band, corresponding to sp^3^ C–C vibrations in paclitaxel, overlapped with the cellular phenylalanine signal.^296^ Parachalil *et al.* (2019) evaluated the limit of detection (LOD) and quantification (LOQ) of two chemotherapeutic agents in human serum during treatment. Using partial least squares regression (PLSR), they determined an LOD for busulfan of 0.0002 ± 0.0001 mg mL^−1^, 30–40 times below toxic levels, and LOD/LOQ for methotrexate of 7.8 ± 5 µM and 26 ± 5 µM, respectively, supporting potential dose monitoring. Key Raman peaks were identified for each drug: Busulfan at 1097 cm^−1^ (CH_2_ scissoring) and 1453 cm^−1^ (C–C stretching), and methotrexate at 1593 cm^−1^ (NH_2_ scissoring) and 1351 cm^−1^ (CH_2_ scissoring).^297^

The use of Raman spectroscopy has been extended to drug discovery, including the identification of new roles for known drugs with unexpected antitumoral activity. For example, Terbinafine is an antifungal agent with emerging interest in its antitumor activity in colorectal cancer.^298^ This drug was used by Wei *et al.* (2014) to monitor its *in vivo* biodistribution in mouse ear tissue following topical application of Lamisil cream.^299^ Using SRS, different subcutaneous layers were imaged *via* the 2230 cm^−1^ signal of the drug's intrinsic alkyne bond, allowing assessment of penetration and tissue distribution. Beyond drug tracking, Dunnington *et al.* (2024) reviewed how Raman spectroscopy can be applied to quantify drug distribution using Raman imaging approaches.^7^ Finally, Braddick *et al.* (2025) provided an extensive review of SRS in drug discovery, emphasizing its potential to monitor drug behavior during treatment.^20^

### 4.3 Functionalized Raman tags for targeted drug tracing within the silent region

The spectral cleanliness of the silent region makes it ideal for the use of bio-orthogonal chemical labels that operate in this window, allowing clear detection of exogenous compounds (from drugs to biomolecules) with minimal background interference. To the best of our knowledge, Yamakoshi *et al.* (2012) introduced for the first time the use of alkyne-tags for Raman imaging (ATRI),^301^ a strategy that established a new paradigm for tracking specific biomolecules in a label-free Raman context by chemically tagging targets such as fatty acids^302^ or drug-cell interactions.^288^ Bakthavatsalam *et al.*, (2021); Benson *et al.* (2022); and Vardaki *et al.* (2024), summarized the most significant advances in alkyne-tag Raman imaging within biological systems,^303–305^ while Wei *et al.* developed a comprehensive library of alkyne-tagged small biomolecules for SRS imaging in the spectral window of 2000–2200 cm^−1^.^299^ While alkyne tags are the most established probes for Raman imaging and sensing thanks to the sharp and intense alkyne peak from –C≡C– bonds,^306^ other chemical groups are capable of producing signals in the silent region, such as nitriles, isonitriles, or molecules containing isotopes of H and C. Among these groups, nitriles generate stronger Raman scattering than azides, while C–D bonds yield weaker, multiplet signals. Bae *et al.* (2019) targeted triphenylphosphonium (TPP), a mitochondria-targeting agent, using an aryl-diyne Raman tag (buta-1,3-diyne-1,4-diylbis(4,1-phenylene)dimethanol, BDDBPDM) due to its minimal background interference, enabling near-real-time quantification *via* SRS measurements at 2216 cm^−1^ (C≡C stretching) in HeLa cells.^300^

## 5 Strategies for experimental design and data analysis

Evaluating therapy responses in human-related models, *via* fluids analysis, or in patients, requires a robust protocol, encompassing careful experimental design, data collection, and analysis to produce meaningful contributions to current knowledge. Fig. 6 presents a summarized workflow outlining the key variables that should be considered in next-generation studies within this field. In this context, the analysis of Raman data is crucial for extracting clinically relevant information. However, this analysis is preceded by key steps: the process starts with experimental design, followed by data preprocessing, feature extraction, and model development – all of which are essential for transforming complex Raman spectral data into interpretable results.

**Fig. 6:**
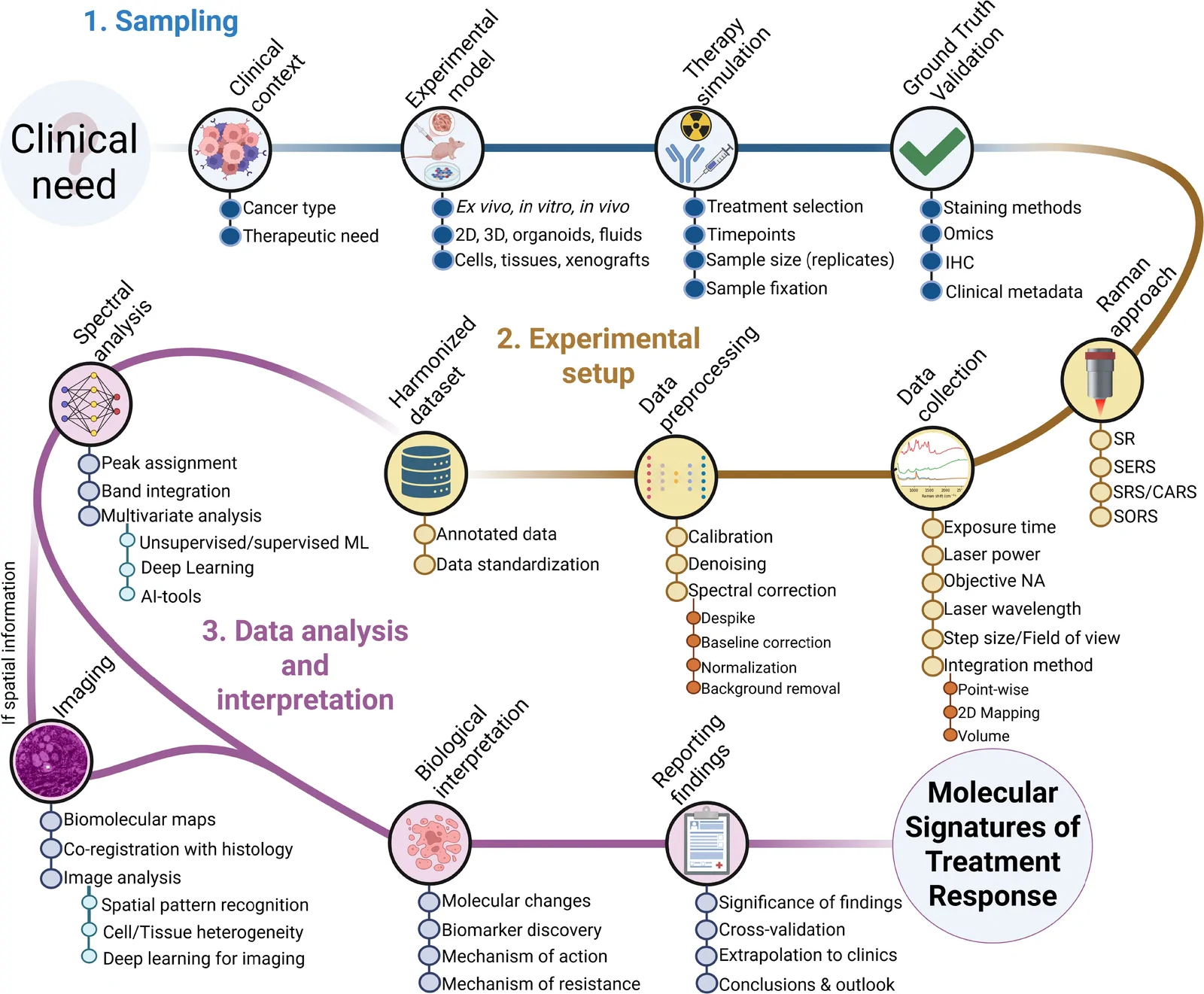
Schematic representation of an experimental workflow utilizing Raman-based methodologies for monitoring the therapeutic response in cancer treatments. Inspired by Guo *et al.*, (2021).^308^ The workflow is structured into three primary phases: sampling, experimental setup, and data analysis and interpretation. Each phase is further subdivided into key components, depicted as large circles, with associated variables or experimental considerations outlined in smaller circles. The workflow emphasizes the integration of spectral analysis, data preprocessing, and advanced imaging techniques to elucidate molecular signatures of treatment response.

### 5.1 Experimental design and sample size planning

The experimental design is especially important for Raman-based biological applications, where both the complexity of Raman spectroscopy and the heterogeneity of biological samples introduce significant challenges. To effectively differentiate the relevant spectral features of the simulated therapy response, it is critical to sample enough replicates, meaningful controls, and time points. Guo *et al.* (2021) highlight the design of experiments as essential for obtaining reliable measurements and validating studies.^307^ Systematic experimental design consists of two key steps: (i) the definition of the measurement protocol, which ensures data consistency and reproducibility while minimizing errors, noise and bias due to instrumental conditions, and (ii) sample size planning (SSP), which ensures enough samples are included to achieve meaningful conclusions. Statistical tools are exploited for a sound SSP, such as the analysis of variance (ANOVA)^308^ across the populations, the analysis of the learning curve of metrics varying the sample size,^309^ the power analysis of statistical hypothesis testing,^310^ where power refers to the magnitude of the probability of rejecting the null hypothesis, and the analysis of sensitivity and specificity in predictions on validation data.^311^ Such approaches for SSP pave the way for the generation of robust inference and prediction models.^307^

An optimal SSP is the one that minimizes the number of biochemical variations extracted through data analysis that are explained by technical and biological variability or noise rather than by the anticancer therapy response under investigation. To this aim, it is vital to plan to include both technical and biological replicates in the SSP: while this is a well-established course of action in biology and biotechnology and in univariate measurements, more awareness still needs to be raised among the Raman community and, more in general, in those biophysical branches dealing with multivariate data.^312^ Technical variability refers to the variations introduced by measurement platforms, personnel, and reagents, while biological variability refers to the variations introduced by the fact that biological systems, such as cells, tissues and human or animal subjects, can only be similar but not identical. While a strong limitation of technical variability is ideal, the inevitable presence of biological variability is beneficial to ensure the generalizability of results. SSP is essential for ensuring rigorous statistical analysis and should be carefully planned, particularly in bio-spectroscopic studies where data generation is both time-consuming and costly. M. Krzywinski and N. Altman pinpointed effective strategies to combine in order to achieve replicable and meaningful results, which should be taken into account in SSP: experimental control, randomization, blocking and replication.^308^ Sampling only a part of the population increases precision, but generalizing these results would produce bias and misrepresentation. A better representation of the therapy response by the biological model can be achieved by randomly sampling across the population, but this can result in excessively variable measurements. Hence, blocking helps: identifying blocks of similar samples within the population increases the precision within each block while capturing population variability between blocks.^313^

A general SSP algorithm based on learning curves was proposed by Nairveen Ali *et al.*, which uses inverse power-law fitting to estimate the necessary sample size. The method was applied to Raman spectroscopy data for differentiating six bacterial species, determining that 142 spectra per species and seven biological replicates were needed to train a statistically valid classification model using PCA and LDA.^309^ Notably, multivariate datasets, such as the ones obtained *via* Raman spectroscopy, intrinsically carry a higher amount of information characterizing the analyte compared to univariate ones. This may lead to a reduced sample size to achieve statistically significant findings compared to univariate datasets tackling the study of the same therapy response. Recently, Bresci *et al.* showed that acquiring Raman maps of only five replicates of living human colon cancer cells in standard culture conditions across different tumoral stages was enough to extract label-free biochemical hallmarks that distinguish between cancer phenotypes.^224^ Ensuring the presence of both technical and biological replicates in the SSP, while acquiring Raman spectral images with high spatial resolution and high signal-to-noise ratio (*i.e.*, a pixel pitch of 800 nm and a pixel dwell time of 1.5 seconds), was key to achieving statistically valid phenotype inference, validated through standard ANOVA, statistical hypothesis testing for mean and median differences (*e.g.*, Student's *T*-test and Mann Whitney *U*-test), cross-validation (*e.g.*, leave-one-out cross-validation (LOOCV)), and regression modeling accompanied by the assessment of inference accuracy (*e.g.*, likelihood ratio testing and use of Akaike's information criterion).

Recent efforts in Raman spectroscopy highlight how thoughtful SSP must be coupled to robust data management. Community initiatives emphasize standardized data formats, metadata, and shared digital workflows for data acquisition and analysis, which facilitate data exchange across collaborating groups and improve reproducibility of multivariate models.^314^ These practical aspects directly influence the balance between technical and biological variability and therefore need to be considered during SSP, alongside the traditional statistical criteria used to determine the number of replicates and samples.^185^

Beyond statistical robustness and experimental reproducibility, Raman-based cancer monitoring must evolve beyond a focus solely on technical reproducibility and align more closely with established clinical research methodologies. Clinical oncology relies on data generated through retrospective and prospective studies, stratified patient cohorts with clear inclusion and exclusion criteria, and well-defined endpoints, such as disease-free or overall survival. This is necessary to ensure that the results of clinical Raman studies are representative of real-world clinical scenarios. Raman investigations should therefore be framed within the standard biomarker development pipeline, progressing systematically from exploratory discovery to analytical and clinical validation. Currently, this appears far from obvious. While hundreds of Raman-related articles are published annually on PubMed, only approximately 160 studies are registered on ClinicalTrials.gov across all fields of medicine. Encouraging study preregistration is therefore a critical step toward integrating Raman spectroscopy into evidence-based oncology and establishing it as a clinically useful tool for cancer management.

### 5.2 Data preprocessing

Preprocessing in Raman spectroscopy applied to biological samples consists of a set of standardized operations applied to raw spectra to remove non-biological contributions and to place all measurements on a common, comparable scale. Typical steps (described in the next paragraphs) reduce technical variability while preserving chemically meaningful spectral features. By doing so, preprocessing improves reproducibility and enables robust comparison across samples, sessions, and instruments, which is also essential for building models that can transfer reliably to clinical workflows.^315,316^

Preprocessing pipelines are inherently experiment- and instrument-dependent, as each specimen type requires a tailored sequence of preprocessing operations. Typically, one of the first preprocessing steps is the cosmic-ray removal. Cosmic rays produce high-intensity spikes in Raman spectra, which can be considered artifacts. Several methods exist to remove these spikes: for single spectra, common approaches involve peak width, prominence, and intensity thresholds to identify and interpolate outliers;^317,318^ for multi-spectral data, cosmic rays can be filtered by comparing peak intensities across spectra and replacing outliers with averages; wavelet transforms and median filters are also employed to preserve real spectral features while removing narrow, high-intensity spikes.^319,320^

In parallel, another fundamental step is calibration. Intensity calibration (also called *y*-axis calibration) is performed to correct the system's wavelength-dependent response (detector quantum efficiency, grating efficiency, and overall optical throughput) by using, for example, calibrated white light sources under the same conditions as Raman spectral acquisition. More details can be found in Ntziouni *et al.* (2022).^321^ For Raman shift calibration (also called *x*-axis calibration), neon, argon, or mercury lamps provide discrete emission lines that enable initial alignment of the wavelength axis, used to establish the absolute spectral positions of the spectrometer. Subsequently, well-characterized reference materials (*e.g.* polystyrene^321^) are employed to refine the calibration and cover the full spectral range.

Calibrated and cosmic-ray-corrected Raman spectra are typically smoothed to enhance the visibility of Raman bands while minimizing noise, preventing it from being misinterpreted as genuine spectral features. Common smoothing approaches include Savitzky–Golay filtering,^322^ which fits successive subsets of data points with a polynomial; singular value decomposition (SVD), which separates signal from noise using matrix decomposition;^323^ and Whittaker smoothing, which penalizes large deviations to produce a smooth baseline.

Background removal is another crucial step in Raman spectroscopy – especially when applying SR approaches – to eliminate unwanted signals from fluorescence, optical substrates, or instrument response, ensuring that only true spectral features from the sample are analyzed. This is especially important when measuring cancer cells or tissues, often fixed or seeded on substrates like quartz, CaF_2_, or glass, which are commonly used in clinical settings. Common standard approaches for background removal include polynomial fitting, which corrects for baseline drift by adjusting the data with a fitted polynomial function, and alternating least squares (ALS), which separates the spectral signals from background interference by optimizing a least-squares solution. These methods, along with their derivatives, are widely used to enhance the accuracy of Raman data. In recent years, new preprocessing tools have emerged to address these challenges. One such method is extended multiplicative signal correction (EMSC), which is commonly applied for background subtraction in cytology samples and does not require additional measurements or secondary datasets, making it a versatile method for cross-instrument model transfer.^324^ Combined with regression or classification methods, EMSC has been shown to improve predictive performance, as discussed by Guo *et al.* (2018),^325^ and as reported by Féré *et al.* (2019), who applied EMSC to harmonize lymphocyte Raman spectra from healthy donors and B-CLL patients, enhancing classification with partial least squares–discriminant analysis (PLS–DA).^315^

Among standard preprocessing steps, global, wavenumber-independent variations in Raman spectra can be addressed using a variety of normalization techniques. Vector normalization scales the spectrum so that its total vector length equals one, area normalization adjusts intensities relative to the integrated spectral intensity, standard normal variate (SNV) centers and scales each spectrum to unit variance, and min–max scaling rescales the spectral intensities to a fixed range, typically between zero and one.^326^ After preprocessing, spectra must be interpolated prior to modelling in order to ensure a common set of Raman shift points along the *x*-axis.

Considering the high variability and possible subjectivity introduced during data preprocessing, Wahl *et al.* (2022) systematically evaluated six preprocessing pipelines on over 900 Raman spectra from 34 fresh brain tumor samples, including gliomas, meningiomas, and metastases. Data exploration using PCA, *t*-distributed stochastic neighbor (*t*-SNE), and *k*-means clustering revealed that while the underlying biochemical features – such as hemoglobin, neuroglobin, carotenoids, water, protoporphyrin, proteins, and lipids – remain largely consistent, their spectral appearance, clustering, and apparent prominence varied significantly depending on the preprocessing strategy. Notably, one pipeline adopting a neural network approach for simultaneous noise reduction and baseline correction enabled rapid and adaptive preprocessing suitable for potential *in vivo* applications in neurosurgery, highlighting the importance of automated pipelines that balance noise removal, baseline correction, and preservation of spectral information.^327^

Overall, preprocessing choices critically influence spectral interpretation, cluster formation, and classification, emphasizing the need for methodological transparency and standardization when comparing Raman studies across different laboratories and clinical settings. The harmonization of Raman spectroscopy data is essential to ensure the comparability, reproducibility, and reliability of results across different instruments, laboratories, and analysis workflows.^326^ Proper data harmonization not only improves the performance (see Fig. 7) of multivariate and machine learning analyses but also enables meaningful *meta*-analyses and collaborative studies. Recently, Coca-López *et al.* emphasized the importance of following the FAIR (findable, accessible, interoperable, and reusable) principles in Raman spectroscopy, highlighting how standardized data formats and preprocessing routines can empower artificial intelligence approaches by facilitating data sharing and integration among the Raman community.^185^ Projects such as MicrobioRaman,^328^ an open-access web repository for microbiological Raman spectroscopy data, are one example of the growing effort in this direction.

**Fig. 7:**
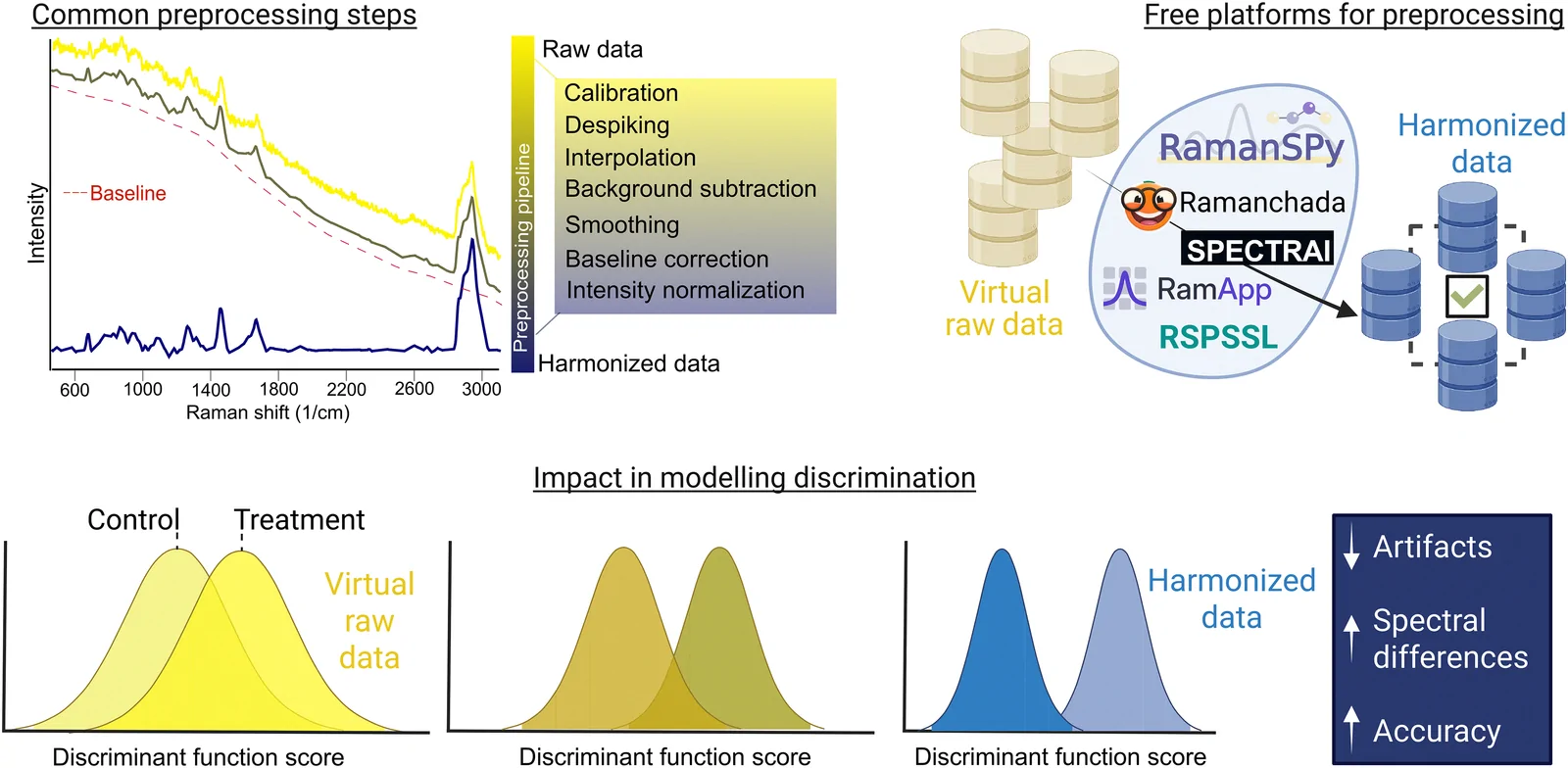
Impact of Raman spectral preprocessing for data harmonization. Raw Raman spectra exhibit baseline variations and artifacts (top left). Preprocessing steps, such as calibration, interpolation, despiking, background subtraction, smoothing, baseline correction, and intensity normalization, reduce variability and enhance spectral comparability. Free toolboxes enable standardized preprocessing (top right). Preprocessed data improves discriminant function score separation between groups, enhancing accuracy (bottom).

Regarding available software and tools that might be used for data preprocessing – apart from those included in most commercial Raman systems – a surge in free and open-source alternatives appeared in the past few years (Fig. 7, upper-right panel). Notable examples include RamanSpy,^329^ a comprehensive Python library; RamApp (https://ramapp.io/),^330^ a user-friendly web platform for exploring hyperspectral Raman maps with processing and analysis tools; SpectrAI (https://www.spectrai.org/),^331^ an open-source PyTorch-based framework that integrates tailored spectral data preprocessing, augmentation, and specialized deep-learning architectures to accelerate AI-driven analysis across spectroscopy and spectral imaging applications; and Ramanchada2,^314^ an MIT-licensed Python package offering state-of-the-art algorithms for generating, reading, visualizing, and processing Raman spectra, with particular emphasis on instrument calibration. Additionally, Hu *et al.* (2024) introduced the self-supervised Raman spectral preprocessing framework called RSPSSL, a high-fidelity, robust, and universal scheme designed to enhance noise reduction and baseline correction while preserving spectral features across instruments, sample types, and measurement conditions. The method combines a generative adversarial network (RSGAN) to generate high-quality reference spectra for training with a multi-scale convolutional neural network (RSBPCNN) capable of processing new spectra without retraining. When applied to biomedical datasets, RSPSSL substantially improved signal clarity, peak definition, and quantitative reproducibility compared to conventional preprocessing, enabling more accurate biochemical interpretations and diagnostic analyses.^332^

### 5.3 Feature analysis and machine learning

In Raman spectroscopy, datasets consist of single-point spectra or hyperspectral images, namely, multivariate datasets containing a significant amount of correlated information. Dimensionality reduction (transforming data into a lower-dimensional space) and feature selection (extracting key vibrational bands) are crucial first steps to achieve effective data visualization and remove redundant information, ultimately extracting the biochemical patterns that govern therapy response. The most common techniques to achieve this include unsupervised methods where no explicit information about the sample state, such as the therapy response stage, is provided,^333–336^ and supervised methods, where labels are fed into the algorithm. Hence, those Raman features identified as most meaningful can be used downstream to mathematically model the biological process of therapy response. The choice of the optimal model can be guided by different standpoints, including the desired level of prior knowledge the model should incorporate, the hypothesis on which the model is based, and its mathematical complexity. Finally, doing all the necessary due diligence for model validation is critical to ensure trustworthy results that reflect the underlying biological phenomena. This is generally achieved through cross-validation sessions onto data partitions, aiming at avoiding overfitting and ensuring generalizability on still unseen Raman data. Such a systematic, end-to-end data processing strategy enables the breakdown of Raman hallmarks that can be used as a proxy for further research studies and for prospective diagnostics. In the following, we cover this three-step rationale in greater detail to guide Raman data users toward effective information retrieval and meaningful biological conclusions.

#### 5.3.1 Dimensionality reduction and feature selection

Unsupervised approaches for dimensionality reduction and feature extraction mainly include PCA (see Fig. 8a), singular value decomposition (SVD), multivariate curve resolution (MCR),^335,336^ and uniform manifold approximation and projection (UMAP, Fig. 8c and d),^333,338^ with PCA arguably being the most widely used approach in the Raman spectroscopy community. Frequently, these dimensionality-reduction techniques are paired with clustering algorithms (*e.g.*, Leiden clustering) to further group observations and extract representative centroid spectra (Fig. 8c and e).

**Fig. 8:**
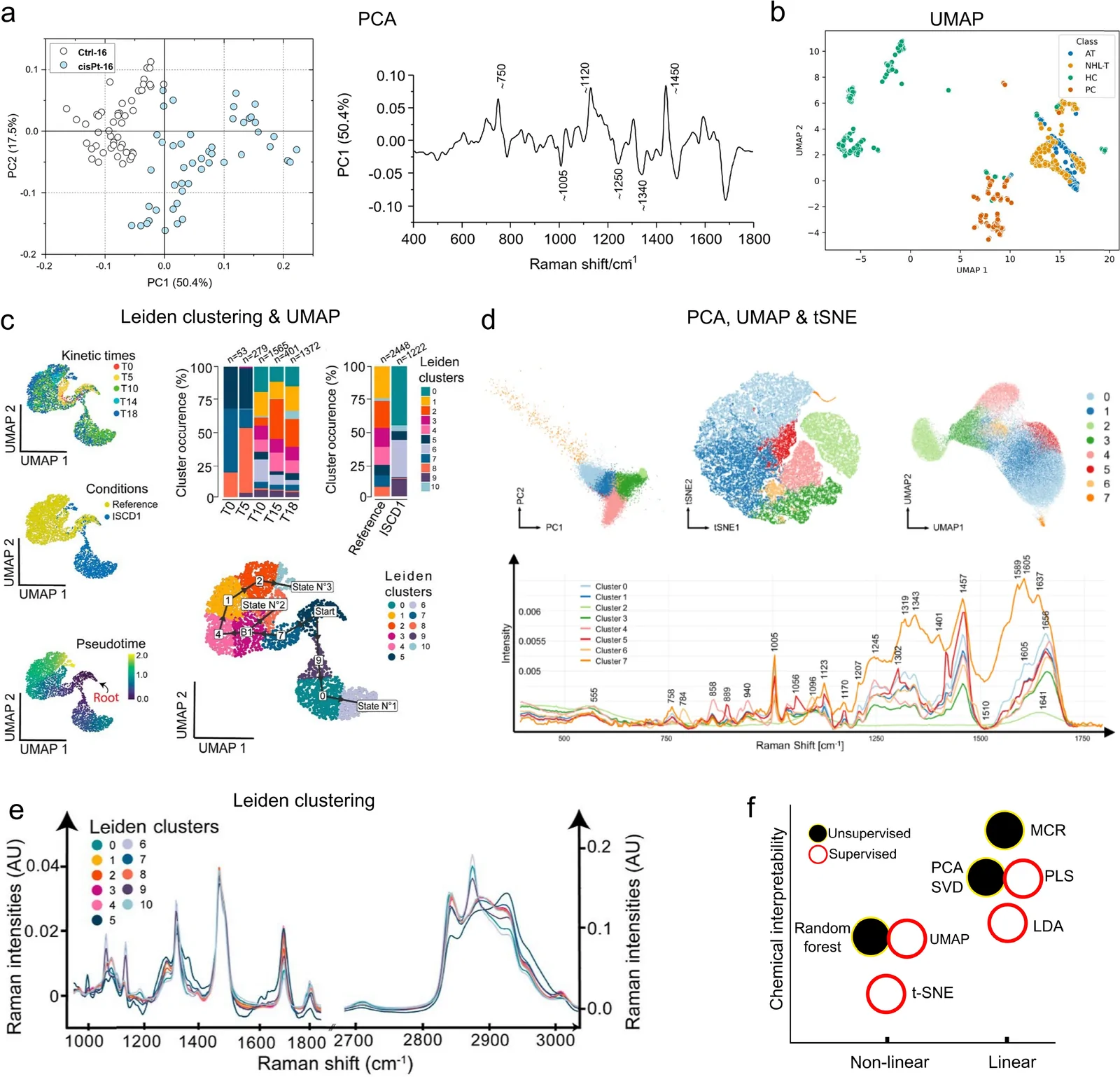
Representative dimensionality-reduction and clustering approaches for cellular and tissue characterization using Raman spectroscopy. (a) PCA score and loading plots illustrate cisplatin-induced spectral changes in primary oral squamous carcinoma cells after 16 h treatment (adapted from Notarstefano *et al.*, 2023^336^ with permission from Royal Society of Chemistry) (b) nonlinear learning is exemplified by UMAP separation of leukemia-related cell models with different radiosensitivities, including healthy controls, prostate cancer patients, AT-deficient and NHL-T lymphoblastoid cell lines (reused from Meade *et al.*, 2025^344^ under a Creative Commons Attribution 4.0 International License). (c) UMAP projections and trajectory inference applied to the differentiation of 3T3-L1 cells into adipocyte-like cells with SCD1 inhibition, displaying data distribution by kinetic times, culture conditions, pseudotime, and relative Leiden cluster occurrences (adapted from Goffin *et al.*, 2023^355^ with permission from American Chemical Society). (d) High-dimensional tissue characterization comparing PCA, *t*-SNE, and UMAP projections, alongside cluster-specific average spectra with characteristic peak annotations (adapted from Sigle *et al.*, 2023^343^ under a Creative Commons Attribution 4.0 International License). (e) Centroid Raman spectra corresponding to the Leiden clusters identified in panel c (adapted from Goffin *et al.*, 2023^355^ with permission from American Chemical Society). (f) Summary of the main dimensionality-reduction and projection methods discussed in Section 5.3, categorized by supervision, linearity, and degree of chemical interpretability.

Being unsupervised, PCA does not retain any *a priori* knowledge regarding the outcome classes of the study, making it a powerful approach to assess the presence of spectral patterns that distinguish the data cloud into subgroups while maintaining straightforward chemical and biological interpretability.^339^ Through PCA, the original spectral data are transformed into a new space composed of a set of orthogonal bases, the principal components (PCs), which describe the directions of variance in the original data in descending order (*i.e.*, the first PC captures the principal direction of variance, and progressively lower variance is explained by later PCs). This is achieved through the calculation of the covariance matrix of the Raman dataset, whose eigenvectors correspond to the PCs. Using only the first PCs, generally up to a cumulative explained variance of 95%, is a quantitatively solid solution for dimensionality reduction. On the other hand, PCs do not necessarily drive the separation between the targeted classes in the dataset, for instance, they may not capture the biochemical patterns describing the different phenotypes,^225^ therapy responses,^224^ or malignancy levels^340^ under investigation. Hence, further regression analysis is needed to quantitatively break down the role of each basis in acting as an independent variable for the target dependent variables. Similarly to PCA, SVD operates in an unsupervised fashion without knowledge of the class labels, but it bypasses the covariance matrix and directly factorizes the Raman spectral dataset into three orthogonal matrices.^155,341^

Unlike PCA-based approaches, which identify orthogonal components describing the main sources of variance in the dataset and require interpretation of the associated loadings, MCR aims to decompose the Raman dataset into two matrices: one containing the pure component spectra, and the other their relative abundance.^335^ This bilinear factorization allows the retrieval of pure spectral signatures of biochemical constituents and the estimation of the relative concentration of each biochemical component per pixel or across the dataset. For example, MCR can resolve spectral profiles for viable, early apoptotic, and late apoptotic/dead oral squamous cell carcinoma cells under cisplatin exposure, and quantify their progression rates over 72 hours.^336^ Refer to the recent Raman-imaging-based study by Schmidt *et al.* for further details.^342^ Finally, UMAP is a non-linear dimensionality reduction technique designed to preserve both local and global relationships within Raman datasets. By constructing a topological representation of the data manifold, UMAP captures subtle spectral similarities that may be obscured in linear embeddings and is widely employed for visualizing spectral clusters associated with phenotypic or treatment-induced variations, making it a powerful exploratory tool for data visualization.^343,344^ However, UMAP faces limitations in terms of quantitative biochemical modeling. Unlike PCA components or MCR-resolved spectral profiles, UMAP dimensions lack direct physical or chemical interpretability; in addition, its performance is highly sensitive to parameter choices and dataset size.

To boost class separation, especially when dealing with very similar biological models where the spectral differences among groups are too subtle to produce clear results with unsupervised algorithms, one can opt for informing the data processing pipeline about class labels. Supervised data processing algorithms, such as PLS^337,345^ and LDA,^229,317^ are highly exploited as downstream analyses on the output of unsupervised algorithms^347,348^ to maximize group separation. PLS is a regression algorithm maximizing the covariance between the spectral data and the known class labels, effectively capturing patterns directly linked to the biochemical differences of interest.^349^ PLS operates by identifying latent variables that not only capture variance in the spectral data but also correlate strongly with the target variable (*e.g.*, the progression of the sample state as a therapy response over time,^350^ or the classification of different sample states induced by different cancer treatments^351^). This dual focus makes PLS especially powerful for cases where subtle spectral differences correlate with meaningful biological responses, but they are spectrally overwhelmed by other biomolecular rearrangements or technical/biological variability. On the other hand, LDA is particularly suited for classification tasks where the objective is to separate predefined classes based on the spectral features. Unlike PLS, LDA is a classification algorithm, along with other well-validated models like support vector machine (SVM), as it works by projecting the data onto a lower-dimensional space that maximizes the separation between classes, which is achieved by optimizing the ratio of between-class variance to within-class variance.^352^ This approach enhances class distinction by drawing on knowledge of class labels, making LDA effective for discriminating between highly similar biological models that might not be easily distinguished with unsupervised methods.^353^ In the most complex scenarios, when minimal spectral rearrangements serve as therapy response hallmarks, the class-separation power of LDA can be increased by exploiting non-linear kernel functions (*i.e.*, kernel discriminant analysis (KDA))^354^ or by applying LDA on a subset of spectral features screened *via* PLS regression (*i.e.*, PLS-DA),^346^ in order to be able to capture more complex patterns in Raman spectra that ultimately determine the therapy outcomes. Furthermore, to account for the continuous and unsynchronized nature of many biological responses, trajectory inference (TI) methods can be applied to Raman spectral data to reconstruct evolving cellular pathways. By ordering individual spectra along a calculated pseudo-time axis, TI algorithms capture the dynamic heterogeneity of cell populations and artificially synchronize complex biological transitions.^355^Fig. 8f provides a schematic classification of the discussed methods based on their chemical interpretability, linearity, and whether they are supervised or unsupervised.

#### 5.3.2 Mathematical modeling

Once dimensionality reduction and/or feature extraction is performed, the relationship between the most meaningful Raman signatures and the desired outcome variables can be modeled mathematically to find quantitative Raman patterns that describe the biological phenomena under study.^307^ Ultimately, statistically validated mathematical models can be exploited to infer the sample state and further predict that state on new samples, in a broader and more generalized fashion. In this phase, statistical evaluation of the model performance on seen and unseen examples is fundamental to allow a trustworthy inference and prediction, respectively.^225^ A high inference accuracy ensures a good fit of the model for the biological phenomena at hand, and a high prediction accuracy of the sample label on data points that were not used during modeling is key to ensure generalizability of the results and circumvent overfitting.^356^ To avoid detrimental biases and mistakes in the conclusions drawn from modelled Raman data, it is fundamental to fully understand the structure, scope, and usage of mathematical models. The most common models used in Raman spectral analysis can be divided into multiple categories, according to which characteristics are considered.

As for the model knowledge of class labels (*e.g.*, the different sample states under investigation), similar supervised and unsupervised algorithms previously discussed for feature analysis and dimensionality reduction can be further exploited for data modeling and prediction. Supervised models, such as PLS, LDA, and support vector machine (SVM), require labeled training data.^254,357^ SVM is a supervised learning algorithm widely used for classification tasks in Raman spectroscopy, particularly in cancer therapy monitoring. SVM works by identifying a hyperplane in the multidimensional feature space that best separates the data into predefined classes, such as different therapy responses or malignancy levels in cancerous biosamples.^358,359^ Conversely, unsupervised models, like PCA and clustering algorithms (*e.g.*, *K*-means clustering and hierarchical clustering^360^), operate without knowledge of class labels, which is valuable for exploratory analyses (Fig. 8d and e). *K*-means clustering, a widely used algorithm in Raman spectroscopy, partitions data into a predefined number of clusters by minimizing the within-cluster variance. It iteratively assigns each data point, *e.g.*, a Raman spectrum, to the cluster with the closest mean value, recalculating cluster centroids until convergence. In such unsupervised clustering approaches, the choice of initialization parameters strongly influences the outcome, requiring extended parameter tuning. However, these mathematical tools are particularly suited for identifying spectral patterns that correspond to different biochemical states or therapy responses, even without prior knowledge of these categories, and are particularly suitable for the analysis of hyperspectral data (maps).^361^

Another critical distinction is between parametric and nonparametric models.^307^ Parametric models, such as linear regression and linear discriminant analysis (LDA), assume a specific mathematical form for the relationship between spectral features and outcome variables. This assumption simplifies the modeling process and allows for easier interpretation but may limit generalizability. Nonparametric models, such as decision trees or kernel-based methods like KDA, do not rely on predefined functional forms, providing greater adaptability to capture intricate spectral patterns in cases where parametric assumptions are invalid, such as a normal distribution of data points. Decision trees are nonparametric models that partition data into subsets based on feature values, forming a tree-like structure of decision rules. Each internal node in the tree represents a decision criterion (*e.g.*, a threshold on a Raman spectral band), and branches correspond to the possible outcomes of that decision, leading to leaf nodes that denote the final predicted classes or outcomes. In Raman spectroscopy, the straightforward structure of decision trees allows for easy interpretation, as the pathway from root to leaf provides a clear set of rules linking spectral variations to biological states.^10^ However, decision trees are prone to overfitting when applied to complex or noisy datasets, such as those encountered in biological systems. This limitation can be mitigated by using ensemble methods, such as random forests or gradient-boosted trees,^362,363^ which combine multiple decision trees to improve predictive accuracy and generalizability.^364^ This flexibility often comes at the cost of higher computational complexity and the risk of overfitting, particularly in datasets with high dimensionality and limited sample sizes, which is often the case for Raman datasets of biological systems.

Ultimately, one can focus on the mathematical architecture of such models, opting for a linear or nonlinear model. While the former is readily interpretable and entails a simpler causal link between the spectral features and the outcome variables, nonlinear models become useful when linear ones fail to identify, monitor, and separate different sample states, which is often the case when dealing with intricate biological phenomena such as therapy response. Nonlinear models have paved the way for artificial neural networks and deep learning, particularly over the last decade.^11,362,365,366^ Neural network architectures consist of layers of interconnected nodes (*i.e.*, the neurons) that iteratively transform input data through weighted connections, ultimately approximating a nonlinear transfer function of the input Raman data into the desired outcome, for example, into the corresponding cancer state.^367^ Beyond their application as predictive models, neural networks are also valuable for data augmentation, a critical advantage in Raman spectroscopy for biomedical applications where having a large sample size is challenging due to the difficulty and cost of experiments, as well as scarcity of biological materials. By leveraging techniques such as generative adversarial networks (GANs) or variational autoencoders (VAEs), synthetic Raman spectra can be generated to simulate additional data points that reflect the variability of real-world samples.^368^ This helps to improve model training, enhance robustness, and mitigate the risk of overfitting, ultimately contributing to more reliable analyses. Another interesting use of neural networks is the denoising of hyperspectral Raman images. In fact, network architectures can be designed to synergistically focus on both the spectral and spatial domain of hyperspectral Raman maps, typically by including *ad hoc* convolutional layers, enabling a holistic extraction of information.^369^ Alternatively, hyperspectral Raman maps often consist of multiple stitched tiles that suffer from physical artifacts like vignetting and mosaic effects. These can be effectively mitigated using linear approaches such as linear discriminant analysis (LDA), as demonstrated by the EVEN (evaluation and enhancement) framework,^370^ that can automatically rank and select the optimal flat-field correction for each spectral channel, ensuring that the spatial and chemical information remains consistent before being fed into more complex, nonlinear diagnostic pipelines.

#### 5.3.3 Model evaluation

The final, critical step to ensure that the insights derived from Raman spectral data are statistically robust, therefore biologically meaningful and clinically actionable, is model evaluation. Model evaluation logic and metrics can be divided into two major modeling categories: inference modeling and prediction modeling.

In inference modeling, the main goals are quantitatively evaluating the overall goodness of fit of a chosen set of models and quantifying the contribution of specific spectral features to biological outcomes. In fact, inference modeling aims at identifying a mathematical model that significantly explains which Raman spectral features (biochemical constituents) explain which biological state (*e.g.*, response to therapy).^371^ In comparing models, the Akaike information criterion (AIC) and the Bayesian information criterion (BIC) are frequently employed to assess model explanatory power while balancing its complexity by including penalization by the dimension of the model's space of parameters.^225,371^ The likelihood ratio test is used to compare nested models, determining whether the inclusion of additional Raman bands improves the explanatory power. Within a specific model, quantification of parameter importance is achieved by performing statistical testing, such as Wald test, verifying whether one or more parameters jointly are significant explanatory variables of the model outcome.^372^ All these tests can be used in a complementary fashion to search for a simpler yet explanatory model, ensuring trustworthy biological conclusions.

In prediction modeling, the focus is shifted to evaluating how well a selected, trained model generalizes to new, unseen Raman spectral data. Optimal prediction models are not overfitted on training data, achieving comparable prediction accuracy and reproducibility on new samples. This is particularly relevant in the context of cancer therapy monitoring, where the ultimate translational goal is to accurately classify biological responses based on spectral data not previously used through modeling.

A large variety of metrics for model performance on validation sets are available, tailored to the nature of the task (*i.e.*, regression or classification). Leave-one-out cross-validation (LOOCV) and *K*-fold cross-validation are among the most used approaches to validate prediction models in Raman spectroscopy. In LOOCV, the model is trained on all available samples except one (*e.g.*, an individual spectrum or a hyperspectral image), which is used for testing. This process is repeated for each sample and the model prediction accuracy is obtained as an average of these repeated tests.^225^ In *K*-fold cross-validation, the dataset is partitioned into *K* subsets, and the model is trained and tested *K* times using a different fold for testing and *K*-1 folds for training. While LOOCV is computationally feasible on small datasets but prone to outliers and strong bias effects by single data points, *K*-fold cross-validation is a powerful asset for larger datasets to achieve robust performance estimation in a computationally efficient way. It is important to note that, when multiple measurements are obtained per sample or patient, data splitting and cross-validation procedures should account for technical and biological replicates to avoid data leakage and overestimated performance. In clinical applications, patient-level validation strategies, such as leave-one-patient-out cross-validation (LOPOCV), are often recommended, where all spectra from one patient are held out during each validation step to assess generalization to unseen subjects.^373^

For classification tasks, (*e.g.*, identifying different rearrangements of tumor cells) model performance metrics include accuracy, sensitivity, specificity, precision, recall, and the *F*1-score.^364,374,375^ The receiver operating characteristic (ROC) curve is also widely used to assess discriminative power of the classifier across different thresholds, with the area under the curve (AUC) providing a threshold-independent measure of performance: a value closer to 1 indicates strong predictive ability, while values near 0.5 suggest random guessing.^376^ For regression tasks, such as the regression of Raman spectra onto the concentration of a reaction product in tumor cells upon treatment,^349,357^ standard metrics such as the mean squared error (MSE) or *R*^2^ score are commonly employed.

Future Raman studies on cancer therapy response should report, at minimum, the biological question and response endpoint, sample type and cohort structure, biological and technical replicates, acquisition parameters, preprocessing workflow, feature-selection strategy, validation design, and the statistical or clinical endpoint used to define response. Without these elements, spectral differences may remain difficult to interpret, reproduce, compare across laboratories, or translate into clinically meaningful decision-making.

## 6 Current Raman-based approaches to predict therapy response

Although Raman spectroscopy has been extensively explored for cancer diagnosis and, more recently, for monitoring treatment-induced biochemical alterations (see Section 7), its application to the prediction of therapy response remains comparatively limited. To date, only a small number of studies have investigated whether Raman-derived spectral features acquired before treatment, or during its early phases, can anticipate subsequent therapeutic outcomes. This is particularly relevant for the early identification of resistance to pharmacological treatments, but requires longitudinal datasets from *in vitro*, *ex vivo*, or *in vivo* models, where spectral data are collected at defined time points and linked to validated biological or clinical endpoints. Typically, the study baseline is set before the initiation of treatment, and the response is evaluated by tracking biochemical and structural changes over time. This leads to the key question: which spectral or biochemical features present at baseline can predict subsequent resistance or sensitivity? Alternatively, which spectroscopic tumor profiles are associated with the most effective therapeutic response?

Overall, Raman-based therapy-response prediction remains less developed than therapy monitoring, with current evidence concentrated in small cohorts, selected cancer types, and heterogeneous analytical pipelines. This makes prediction one of the most promising but also least mature directions for translational Raman oncology.

Nawaz *et al.* worked in this field in their studies in 2010 and 2011. First, they applied SR spectroscopy combined with partial least squares (PLS) regression to develop a predictive tool for A549 lung adenocarcinoma cell response to cisplatin, focusing on measurements of cell-extracted DNA.^357^ Then, in 2011, they extended their analysis to cell-extracted proteins.^377^ The first study revealed that Raman bands associated with DNA bases, including thymine (737–742 cm^−1^, 1589–1595 cm^−1^), guanine (1548–1553 cm^−1^), and adenine (1578 cm^−1^, 1604–1611 cm^−1^), showed significant intensity changes proportional to cisplatin concentration, reflecting direct Pt–DNA adduct formation and intra-strand crosslinking. Structural perturbations of the DNA backbone were evident from shifts in the A-form (798–813 cm^−1^) and B-form (832–833 cm^−1^) bands, indicating conformational transition from the hydrated B-DNA (physiological form) toward the more compact A-DNA (typical of drug-damaged DNA). In addition, variations in amide III protein bands (1225–1259 cm^−1^) and amide I (1660–1679 cm^−1^) correlated with decreased cell viability, suggesting denaturation during cellular apoptosis and nucleic acid repair responses.^357^ The second study, measuring protein extracts from A549 cells, showed an increase in the intensities of bands associated with C–C skeletal stretching (904 cm^−1^, 935 cm^−1^), phenylalanine (1001 cm^−1^), C–N stretching (1061 cm^−1^, 1131 cm^−1^), indicating direct cisplatin binding to proteins, causing structural changes like unfolding and exposure of aromatic side chains (phenylalanine, tyrosine, tryptophan). Additionally, shifts in the amide I (1660 cm^−1^) and amide III (1252 cm^−1^) bands suggest cisplatin-induced conformational changes in proteins. Intensity changes in lipid-related bands (1420–1460 cm^−1^) further indicate cisplatin interaction with cell membrane lipids, affecting membrane structure. Spectral features linked to these changes were used to predict cisplatin concentration (root mean square error of prediction, RMSEP = 9.48%) and cell viability (RMSEP = 12%), demonstrating how Raman spectroscopy can predict drug interaction and cellular response.^377^

In 2021, Nguyen *et al.* investigated the impact of neoadjuvant chemoradiotherapy (neoCRT) on esophageal cancer (OC) by employing Raman and Fourier transform infrared (FTIR) imaging on tissue samples from 31 OC patients prior to treatment. Their goal was to develop a computational model using spectral data of tumor samples to predict the response to neoadjuvant chemoradiotherapy. Initially, the researchers classified the tissues into four tissue regression grades (TRG), ranging from 1 (full pathological response) to 5 (no response). For the Raman spectra of stromal tissues, the *K*-nearest neighbors (KNN) algorithm produced optimal models to differentiate between TRG 1–2 and TRG 3–4, with AUCs of 0.72 and 0.80, respectively. The highest AUC of 0.85 was achieved with a PCA-quadratic discriminant analysis (QDA) model distinguishing TRG 1 from TRG 2–4. The least accurate models were those classifying TRG 1–2 *versus* TRG 3–4(–5), using both Raman and FTIR spectra. Complementary *in vitro* studies with radio-sensitive (OE33 P) and radio-resistant (OE33 R) cell lines under ionizing radiation treatment found an intensity decrease in the band at 977 cm^−1^, commonly associated with the stretching of the sulphate linkage (–C–O–S–) in glycoproteins, emerging as a potential biomarker that effectively discriminated between the two phenotypes; however, its specific molecular significance remained uncertain in the study. Overall, these results suggest spectral distinctions between patients with full (TRG 1), partial (TRG 2–3) and absent (TRG 4–5) responses.^378^

More recently, Cutshaw *et al.* (2025) used SR of pre-treatment serum samples from locally advanced rectal cancer (LARC) patients to identify key metabolic biomarkers that predict response to neoadjuvant chemoradiation therapy. Raman peaks at 852 cm^−1^ (glucose and glycerol) and 957 cm^−1^ (saturated fatty acids) showed increased intensities in poor responders, indicating a reliance on glycolysis and lipid biosynthesis. The 852 cm^−1^ peak was notably higher in non-responders, suggesting continued metabolic activity in glycolytic pathways, linking glucose metabolism to lipid synthesis. Conversely, 1207 cm^−1^ (amino acids) and 1257 cm^−1^ (long-chain lipids) exhibited decreased intensities in responders. These findings show that 852 cm^−1^ and 957 cm^−1^ are potential metabolic biomarkers of poor prognosis, with elevated intensities correlating with treatment resistance and metabolic reprogramming in tumors. Integrating these pre-treatment Raman markers with serum cytokines (IL-1β, IL-8, IL-2) and DNA-damage-response proteins (p53, MDM2, ATR, CHK1/2) significantly improved the differentiation between responders and non-responders, underscoring the potential of Raman-based liquid biopsies for early therapy prediction.^247^ Galant *et al.* (2026) used Raman spectroscopy on plasma from 40 advanced NSCLC patients, collected before treatment (*t*_0_) and three months after starting first-line pembrolizumab (chemo-immunotherapy, *n* = 29; monotherapy, *n* = 11). Shifts at 1343 and 2934 cm^−1^, corresponding to CH_2_ vibrations from lipids and proteins, predicted patients unlikely to benefit from therapy (short-responders).^379^

Finally, Jia *et al.* (2025) used SR to predict the efficacy of neoadjuvant therapy (NAT) for HER2-positive breast cancer by analyzing serum-derived exosomes collected before and after two cycles of TCb + HP (T: paclitaxel, Cb: carboplatin, H: trastuzumab, P: pertuzumab). In a cohort of 56 patients undergoing standard surgery and Miller–Payne (MP) pathological grading, the authors applied PCA, LDA and SVM (70/30 train–test split), achieving an AUC > 0.89 (Fig. 9). Therapy-driven biochemical alterations were dominated by changes in nucleic-acid-associated modes, particularly the symmetric phosphate stretching peaks at 831 and 880 cm^−1^, which progressively weakened as NAT inhibited RNA transcription and DNA replication. Notably, protein-related amide I (1664 cm^−1^) band intensified with increasing treatment response, reflecting greater ordering of protein secondary structure. To enhance early prediction of NAT efficacy, only differential spectral bands (pre- *vs.* post-treatment) were used for modelling, yielding AUC values >0.9 across all response groups.^380^

**Fig. 9:**
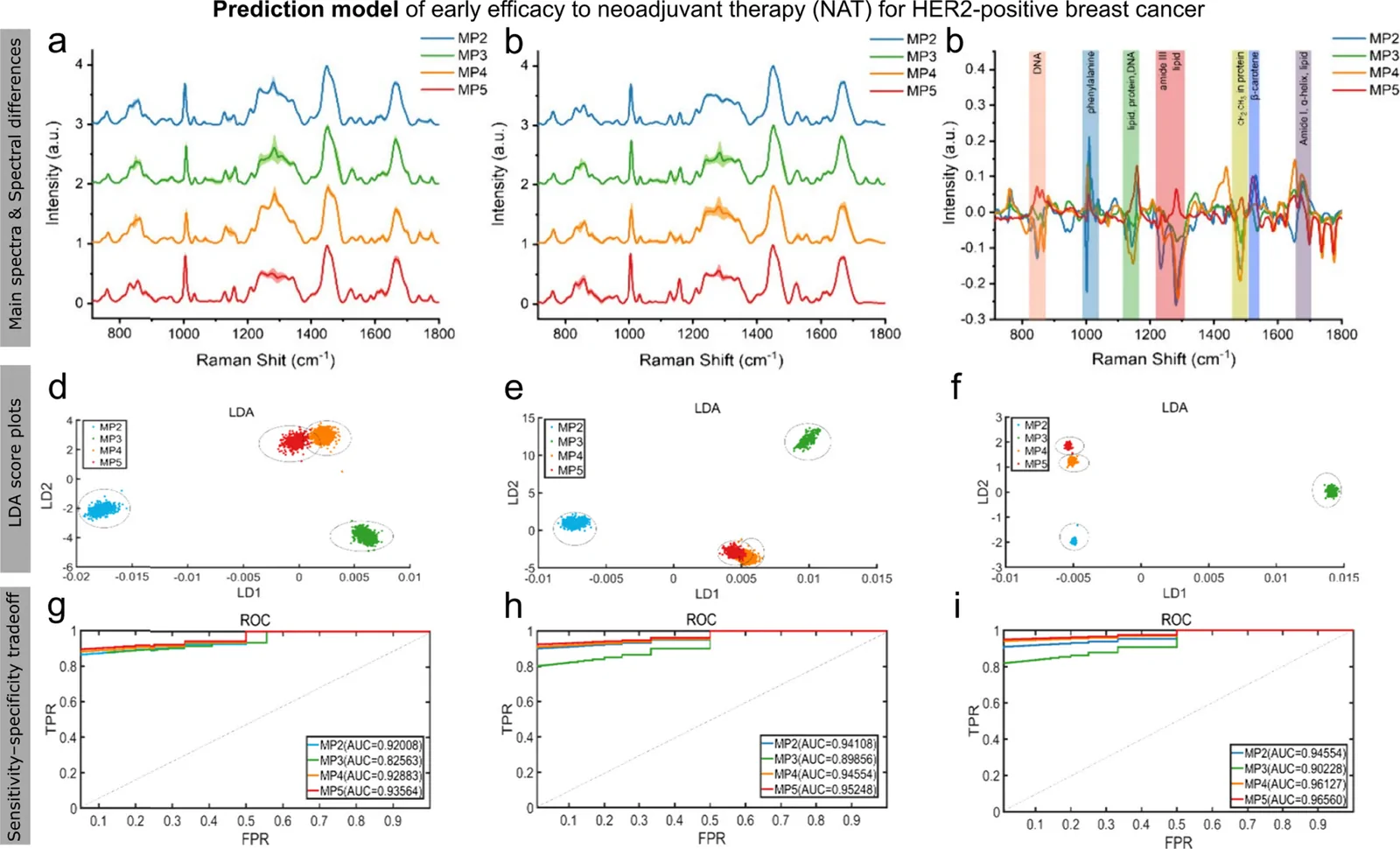
Early prediction of neoadjuvant therapy (NAT) response in HER2-positive breast cancer using label-free Raman spectroscopy of exosomes. (a) Mean pre-treatment Raman spectra across NAT efficacy groups (Miller–Payne, MP2–MP5). (b) Mean post-treatment Raman spectra for the same groups. (c) Differential spectra (post-treatment minus pre-treatment) highlighting therapy-induced spectral changes; shaded bands indicate key Raman regions. (d)–(f) Linear discriminant analysis (LDA) score plots showing group separation for the datasets in (a), (b), and (c), respectively. (g)–(i) Receiver operating characteristic (ROC) curves for the corresponding test sets, with AUC values reported in each panel. MP: pathological Miller–Payne assessment. Adapted from Yining Jia *et al.*, 2025^380^ with permission from American Chemical Society.

## 7 Current Raman-based approaches to monitor therapy response

Raman-based approaches provide detailed molecular information at the subcellular level, enabling the study of biochemical changes, treatment responses, spectral metabolic biomarkers, and cellular heterogeneity without the need for extensive sample preparation, ultimately promoting personalized medicine.^381^ In this section of the literature review, the studies about therapy response monitoring are categorized based on the treatment applied in the specific experimental model. Across monitoring studies, Raman spectroscopy is most powerful when it does not merely classify treated and untreated samples, but links spectral changes to interpretable biological processes such as nucleic-acid damage, lipid remodeling, metabolic adaptation, extracellular-matrix reorganization, drug accumulation, or immune-mediated cytotoxicity.

### 7.1 Monitoring therapy response across preclinical models

#### 7.1.1 Radiotherapy

Preclinical models remain the main testing ground for Raman-based therapy monitoring, as they allow controlled perturbation of treatment type, dose, timing, and biological context. However, the interpretation of Raman signatures strongly depends on the model used, since 2D cultures, spheroids, organoids, xenografts, and patient-derived tissues capture different levels of tumor heterogeneity and microenvironmental complexity.

The biomolecular effects of radiotherapy have been extensively evaluated across various cell lines, with Jirasek *et al.* leading significant contributions by SR. Initially, in 2011, Matthews *et al.* established a protocol to determine the biochemical signatures of *in vitro* radiation response in human lung, breast, and prostate tumor cells. In this way, PC3, DU145, LNCaP (prostate), MDA-MB-231, MCF7 (breast), and H460 (lung) were cultured and irradiated with doses of 15, 30, or 50 Gy, followed by analysis at 0-, 24-, 48-, and 72-hours post-irradiation. Raman spectra were collected using a 785 nm laser, with 200 spectra analyzed per cell line. PCA was employed to identify spectral changes in the fingerprint region. Radioresistant cells exhibited an increased concentration of aromatic amino acids, such as phenylalanine, tyrosine, and tryptophan (1003, 1175, 1208 and 1618 cm^−1^), followed by a reduction in β-sheet protein structure. This suggests that cellular survival mechanisms were activated in response to radiation-induced oxidative stress, breaking down β-sheet proteins into free amino acids, which might help neutralize the damaging effects of reactive oxygen species caused by radiation. Additionally, a rise in α-helix protein suggests the cell production of anti-apoptosis factors such as survivin, a radioresistance factor involved in other cancer types.^382^ Four years later, in 2015, a complementary work was performed by Harder *et al.*, who explored the radiation-induced biochemical responses of four human epithelial tumor cell lines: H460, MCF7, MDA-MB-231 and LNCaP. H460 and MCF-7 were used as radioresistant p53 wt cell lines. Cells were irradiated with clinically relevant doses (2–10 Gy), and analyzed at 18-, 42-, and 66-hours post-irradiation. Raman spectra were collected using a 785 nm laser, with 20 spectra acquired per population, totaling 480 spectra per experiment. PCA identified dose- and time-dependent trends in biochemical changes in the fingerprint region. Glycogen-related spectral features (480, 939, 1388 cm^−1^) were notable in H460 and MCF7, while the phospholipid peak (717 cm^−1^) was significant in LNCaP. Protein-related peaks at 1445–1660 cm^−1^ increased in MDA-MB-231 at higher doses (≥6 Gy). The results linked glycogen accumulation to radioresistance in H460 and MCF7, while phospholipid changes in LNCaP were associated with radiosensitivity.^383^

In 2016, by using single-cell Raman spectroscopy, Matthews *et al.* examined the radiation-induced glycogen accumulation in H460, MCF7, and LNCaP cancer cells. A total of 54 samples (three replicates per dose; six doses ranging from 0 to 10 Gy) were analyzed. The data were processed using PCA, identifying key glycogen bands at 484 cm^−1^, 941 cm^−1^, and 1378 cm^−1^ as markers of resistance to radiation therapy. Treatment with metformin reduced glycogen levels in MCF7 cells, enhancing their radiosensitivity.^280^ New statistical approaches were introduced in 2021, when Milligan *et al.* expanded the analysis of the same cancer cell lines using group and basis-restricted non-negative matrix factorization (GBR-NMF). GBR-NMF scores were validated using Pearson correlations with PCA-derived scores. Key bands associated with glycogen (484 cm^−1^, 1047 cm^−1^), phosphatidylcholine (717 cm^−1^), and glucose (1126 cm^−1^) were identified as metabolic biomarkers associated with radioresistance.^384^ Xenografts were incorporated into their studies in 2018 and 2022. In the first work, Van Nest *et al.* (2018) investigated radiation response in triple-negative human breast adenocarcinoma (MDA-MB-231) xenografts irradiated with 0, 5, or 15 Gy and harvested at days 1 and 3 post-treatment. Using *ex vivo* Raman mapping (420 to 1800 cm^−1^) followed by PCA, the authors identified two dominant components: PC1 captured increased contributions from nucleic acids and aromatic amino acids, showing a significant rise at day 1 after 15 Gy followed by a strong shift toward negative values at day 3, consistent with transient S-phase enrichment and later redistribution into G1. PC2 reflected contrasts between nucleic acid, α-helix protein and lipid features and opposing protein-associated bands (*e.g.*, proline, tryptophan, β-sheet signatures), decreasing consistently at 15 Gy on both days.^385^ Then, Xinchen Deng *et al.* employed the GBR–NMF framework, which included spectra from 40 biological components (amino acids, metabolites, nucleic acids, and lipids), to identify a broad spectrum of radiation-response biomarkers and hypoxia-related indicators in H460 human lung cancer cells and H460 xenografts. Raman maps, acquired using a Renishaw Raman system with a 20 µm step size over a 500 × 600 µm^2^ region with a 100× Leica dry objective, were co-registered with carbonic anhydrase IX (CAIX) immunofluorescence images to correlate Raman signals with hypoxia. Using tissue irradiated at 15 Gy and harvested after 3 days, the authors observed higher average glycogen scores inside the hypoxia mask compared with non-hypoxic regions. Alanine also showed higher average scores within hypoxic regions, and radiotherapy-induced effects were further evidenced by statistically significant increases (*p* < 0.05) in alanine, asparagine, and glycogen chemical scores following radiation treatment.^10^

In 2024, Fuentes *et al.* developed a CNN model to classify Raman spectra from three human tumor cell lines as radiosensitive (LNCaP) or radioresistant (MCF7, H460). Using gradient-weighted class activation mapping (Grad-CAM), they identified high contributions of Raman bands linked to glycogen (484, 1047 cm^−1^), amino acids (1340 cm^−1^), and lipids (1743 cm^−1^) as dominant in radioresistant H460 and MCF7 cells (see Fig. 10b). These bands reflect metabolic reprogramming in resistant phenotypes, including enhanced amino acid and lipid metabolism for energy production. In contrast, radiosensitive LNCaP cells showed increased lipid and phospholipid signals.^386^

**Fig. 10:**
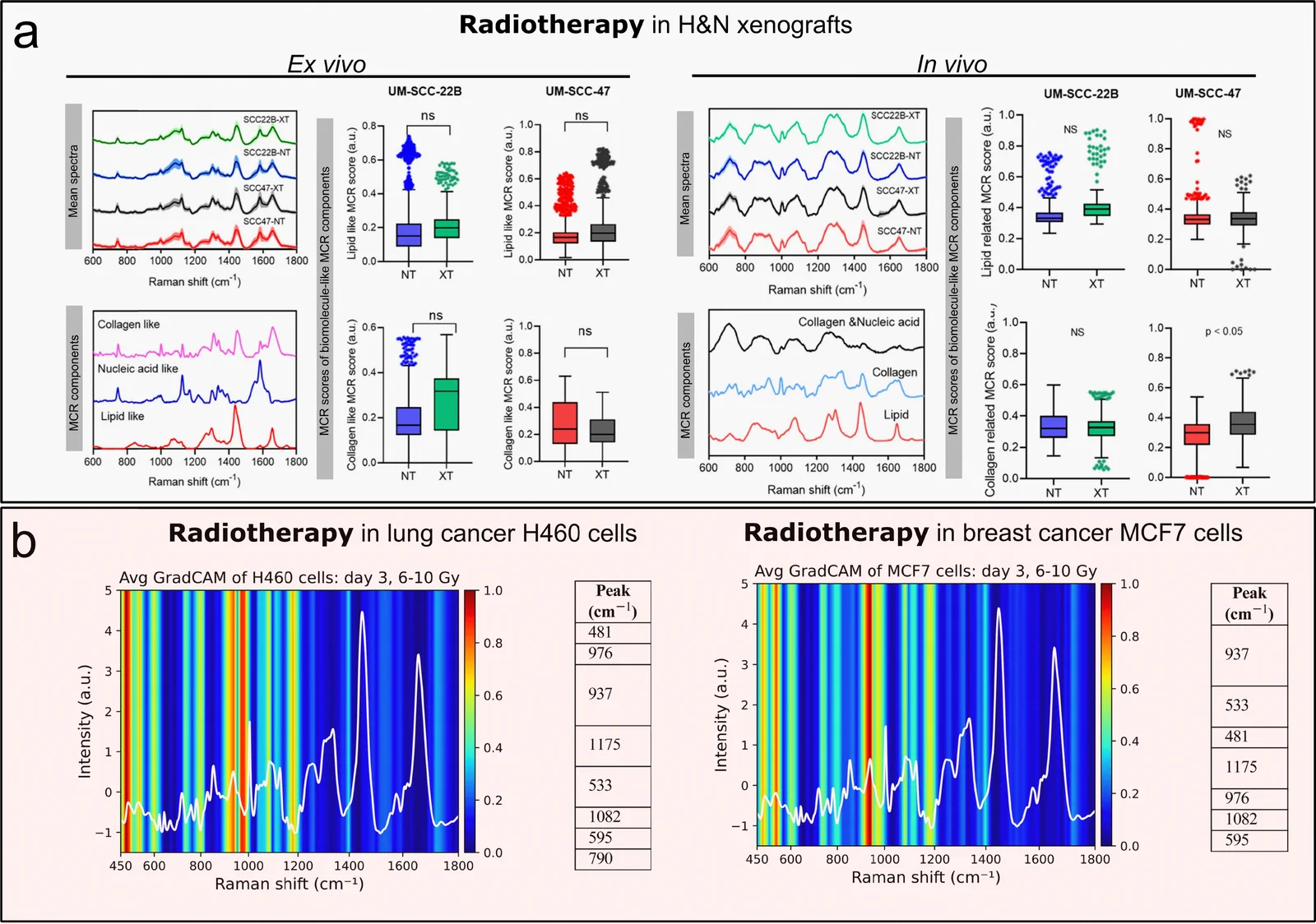
Representative examples of radiotherapy monitoring using label-free Raman-based approaches. (a) MCR-ALS spectral decomposition of *ex vivo* (left panel) and *in vivo* (right panel) head and neck tumor xenografts (UM-SCC-22B and UM-SCC-47) reveals lipid, collagen, and nucleic acid components. Boxplots of MCR scores highlight significant differences in lipid-rich and collagen-rich coefficients after radiation exposure (adapted from Karunakaran *et al.*, 2024^12^ under a Creative Commons Attribution 4.0 International License). (b) Grad-CAM maps of lung cancer H460 cells (left panel) and MCF7 breast cancer cells (right panel) treated with radiotherapy (6–10 Gy), showing key Raman peaks at 481, 976, and 937 cm^−1^, associated with glycogen and lipids (adapted from Fuentes *et al.*, 2024^386^ with permission from American Chemical Society).

The radiotherapy effects in human glioma cells (UVW) were analyzed in 2023 by Hill *et al.*, mainly focusing on the impact of radiation on the cell cycle in brain tumors. A total of 60 samples were analyzed across synchronized (G1/S boundary) and unsynchronized conditions, post-irradiation with fractionated doses (1.5–3 Gy). Raman bands associated with proteins (amide I at 1660 cm^−1^, amide III at 1245 cm^−1^) and lipids (1445 cm^−1^) were identified as markers of radiation response, using PCA for the statistical analysis. Unsynchronized cells exhibited more pronounced spectral changes, highlighting the role of cell cycle phase in modulating radiation sensitivity.^387^

Paidi *et al.* (2019) used SR to monitor the xenograft response to radiotherapy, focusing on tumor microenvironment changes.^388^ In this work, they used an 830 nm fiber-optic Raman system to map formalin-fixed tumor xenografts generated from radiation-sensitive A549 lung carcinoma and its radiation-resistant clone rA549, and from head and neck cell lines UM-SCC-22B (radiation-sensitive) and UM-SCC-47 (radiation-resistant). Tumors were analyzed either untreated (NT) or after fractionated irradiation (4 × 2 Gy over two weeks; for a total of 8 Gy). Using MCR-ALS, the authors recovered components corresponding to lipids, collagen, and, prominently in the lung model, glycogen. Relative to NT controls, the radiation-sensitive cohorts (A549, UM-SCC-22B) showed markedly larger increases in lipid- and collagen-related scores after treatment, whereas the resistant cohorts (rA549, UM-SCC-47) exhibited smaller shifts. In untreated tumors, rA549 xenografts also showed higher lipid and collagen and lower glycogen scores than parental A549, while glycogen was not a significant feature in the head and neck squamous cell carcinoma (HNSCC) set. These results indicate enhanced lipid and matrix remodeling in radiosensitive tumors and a collagen-rich microenvironment associated with intrinsic resistance, rather than a decrease of the 1445 cm^−1^ band or lipid degradation.

Karunakaran *et al.* (2024), to the best of our knowledge, conducted the first *in vivo* evaluation of early tumor biomolecular responses to radiation therapy in murine xenografts. The study employed two HNSCC cell lines: UM-SCC-22B (radiation-sensitive) and UM-SCC-47 (radiation-resistant). Tumors were established in 47 athymic nude mice, and Raman spectroscopy was used to monitor biochemical changes at baseline, 24 hours, and 48 hours after a single 2 Gy dose. The analysis revealed a significant increase in lipid-like species in radiation-sensitive tumors at 48 h post-treatment (*p* < 0.05), suggesting that lipid accumulation may serve as an early biomarker of therapy responsiveness. In contrast, radiation-resistant tumors exhibited a significant increase in collagen-related signals at 24 h (*p* < 0.05), consistent with extracellular matrix remodeling processes associated with resistance (Fig. 10a).^12^

Taken together, radiotherapy studies indicate that Raman signatures of response are dominated by nucleic-acid damage, glycogen and lipid metabolism, and extracellular-matrix remodeling. However, these markers are not universal in a strict sense: their direction and biological meaning depend on tumor type, radiosensitivity phenotype, time after irradiation, and whether the model is *in vitro*, *ex vivo*, or *in vivo*.

#### 7.1.2 Immunotherapy

Immunotherapy remains one of the least explored treatment modalities using label-free Raman spectroscopy, with most studies relying on SERS-based approaches. Nevertheless, recent work highlights the emerging potential of SR in this field. Altangerel *et al.* (2023) introduced thermostable Raman interaction profiling (TRIP), a label-free method enabling low-dose, low-concentration measurements of protein–ligand binding under physiologically relevant conditions.^389^ The approach supports high-throughput drug screening and real-time characterization of interactions spanning small molecules to therapeutic antibodies in solution. Beyond molecular binding, Raman spectroscopy has also been applied to probe the immune landscape of the TME: from assessing macrophage polarization,^390^ to identifying T-cell populations,^391^ to quantifying PD-L1 expression in glioblastoma,^392^ or to monitoring immune infiltration in prostate cancer treated with brachytherapy.^393^ These advances point toward a future in which AI-assisted Raman analysis could support the prediction of immunotherapy response, as recently proposed by Chadokiya *et al.* (2025).^6^

A representative example of Raman-based analysis of immunotherapy response is provided by Paidi *et al.* (2021), who examined CT26 murine colorectal tumor xenografts treated with ICIs targeting CTLA4 and PD-L1. Mice were treated with three doses of either anti-CTLA4 or anti-PD-L1, and tumor slices were analyzed on day 10 using Raman spectroscopy with an 830 nm diode laser. Complementary analyses, including proteomics, identified 136 differentially expressed proteins, many related to lipid metabolism and extracellular matrix (ECM) composition. Histopathology showed no significant differences in tumor necrosis or fibrosis between the treated and control groups. Raman spectra revealed that anti-CTLA4 treatment significantly increased lipid content, with prominent Raman shifts at 1301 cm^−1^ and 1445 cm^−1^, while anti-PD-L1 treatment resulted in a decrease in lipid content. Both therapies reduced nucleic acid levels, as indicated by shifts at 785 cm^−1^ and 1096 cm^−1^, with anti-CTLA4 further reducing collagen content (928 cm^−1^, 1452 cm^−1^). Support vector machine (SVM)-based classification models accurately distinguished between the effects of anti-CTLA4 and anti-PD-L1, reflecting the distinct metabolic signatures induced by each therapy.^394^

Recently, the group of Jennifer A. Dionne introduced a work based on single-cell Raman spectroscopy and machine learning (ML) platform to predict melanoma resistance to targeted therapies and immunotherapies. Using Raman spectra acquired in the fingerprint region with a 532 nm excitation wavelength, the authors first demonstrated the ability of their approach to characterize TME, discriminating major cell populations and environmentally induced macrophage polarization states (M0, M1-like, and M2-like) with >96% accuracy. Feature importance analysis identified the tryptophan-associated band at 750 cm^−1^ as a key spectral marker distinguishing tumor-suppressive M1-like macrophages. To capture early metabolic adaptations preceding stable resistance phenotypes, the authors exposed persistent murine (YUMM1.7, YUMMER1.7) and human (A375, SK-MEL) melanoma cells to IC_50_ concentrations of targeted inhibitors (bemcentinib, cabozantinib, dabrafenib) and the immune checkpoint inhibitor nivolumab for 24 h. Raman differential intensity analysis revealed treatment-specific biochemical alterations; notably, AXL-positive melanoma cells treated with bemcentinib exhibited reduced contributions from protein-associated bands (1000, 1250, 1336, 1438, and 1660 cm^−1^) and lipid-related vibrations (1081, 1438, and 1660 cm^−1^), reflecting metabolic remodeling associated with AXL pathway inhibition. Finally, to evaluate clinical applicability, the authors analyzed nine patient-derived metastatic melanoma samples with distinct therapeutic resistance profiles. To account for intratumoral heterogeneity, single-cell Raman spectra were integrated into patient-like spectral subsets termed “mini patients” and classified using a two-stage random forest workflow. This strategy accurately predicted clinical resistance outcomes for 30 of 33 patient–drug combinations (91% accuracy), including responses to immune checkpoint combinations such as nivolumab plus relatlimab.^395^

These studies suggest that Raman spectroscopy could provide a label-free readout of immunotherapy-induced remodeling of the tumor microenvironment, particularly through lipid, nucleic-acid, and extracellular-matrix signatures. Nevertheless, this area remains at an early stage, and future studies will need to integrate Raman readouts with immune profiling, spatial histology, and clinically relevant response endpoints.

#### 7.1.3 Chemotherapy

Doxorubicin (DOX), the most widely used anthracycline chemotherapeutic for solid and hematologic cancers, has been extensively studied using Raman spectroscopy. Between 2015 and 2017, the group of H. Byrne carried out a series of investigations to characterize the cellular response to DOX with subcellular resolution.^258,293,396^ Their work primarily aimed at tracking the intracellular distribution of DOX and quantifying its impact on major biomolecular components including DNA, proteins, and lipids in cancerous cells. In their first study, they focused on A549 cells exposed to DOX at its IC_50_ concentration (0.55 µM) for 24 hours.^396^ They observed significant decreases in DNA-related Raman peaks (669, 728, 782, 830, 1095, 1340, and 1425 cm^−1^), which indicated DOX intercalation into DNA, causing conformational changes (such as a B-to-A-form transition) and the induction of apoptosis. Minor reductions in protein (1005, 1320, 1578, 1665 cm^−1^) and lipid (1303, 1440 cm^−1^) peaks reflected early apoptotic effects while preserving membrane integrity. Principal component analysis (PCA) confirmed clear spectral differences between control and DOX-treated cells, highlighting the spectral contributions of DOX and apoptotic markers related to nucleic acids and proteins. Then, the researchers extended their study to include Calu-1 cells, comparing Raman data with flow cytometry and reactive oxygen species (ROS) expression.^293^ Similarly to A549 cells, DOX was found to predominantly localize in the nucleolar and nuclear regions. Notably, they detected a decrease in peaks at 847 cm^−1^ and 960 cm^−1^, indicating modifications to the ribose phosphate backbone of DNA due to DOX intercalation, as shown in Fig. 11c. The DNA-related peaks (669 cm^−1^, 728 cm^−1^, 782 cm^−1^, and 830 cm^−1^) decreased, consistent with the inhibition of DNA synthesis and the induction of early apoptosis. Furthermore, the Raman spectra revealed a shift in the 1095 cm^−1^ peak, indicating that DOX was binding to DNA externally, in addition to intercalating within the helix. PCA analysis again confirmed clear spectral differences between the control and DOX-treated cells. Finally, in 2017, the team expanded the analysis by collecting data from additional time points (2, 6, 12, 24, 48, and 72 hours) and incorporating over 210 cells per cell line, producing a comprehensive dataset of 1260 spectra.^258^ This allowed them to monitor the evolution of DOX localization over time. Raman peaks at 440, 465, 1085, 1215, and 1245 cm^−1^ (markers for DOX location) were observed in both the nucleolar and nuclear regions of Calu-1 cells as early as 2 hours after exposure, confirming rapid DOX uptake. They also observed the presence of DOX-induced guanine and cytosine peaks (1430–1450 cm^−1^), indicating DNA intercalation. As time progressed, they recorded a decrease in DNA-related peaks (785 cm^−1^, 813 cm^−1^), reflecting DNA and RNA synthesis inhibition, consistent with the known mechanism of action of DOX. By using PCA, the researchers were able to track the intensity of the DOX band at 465 cm^−1^ over time, showing its concentration in the nuclear and nucleolar regions.

**Fig. 11:**
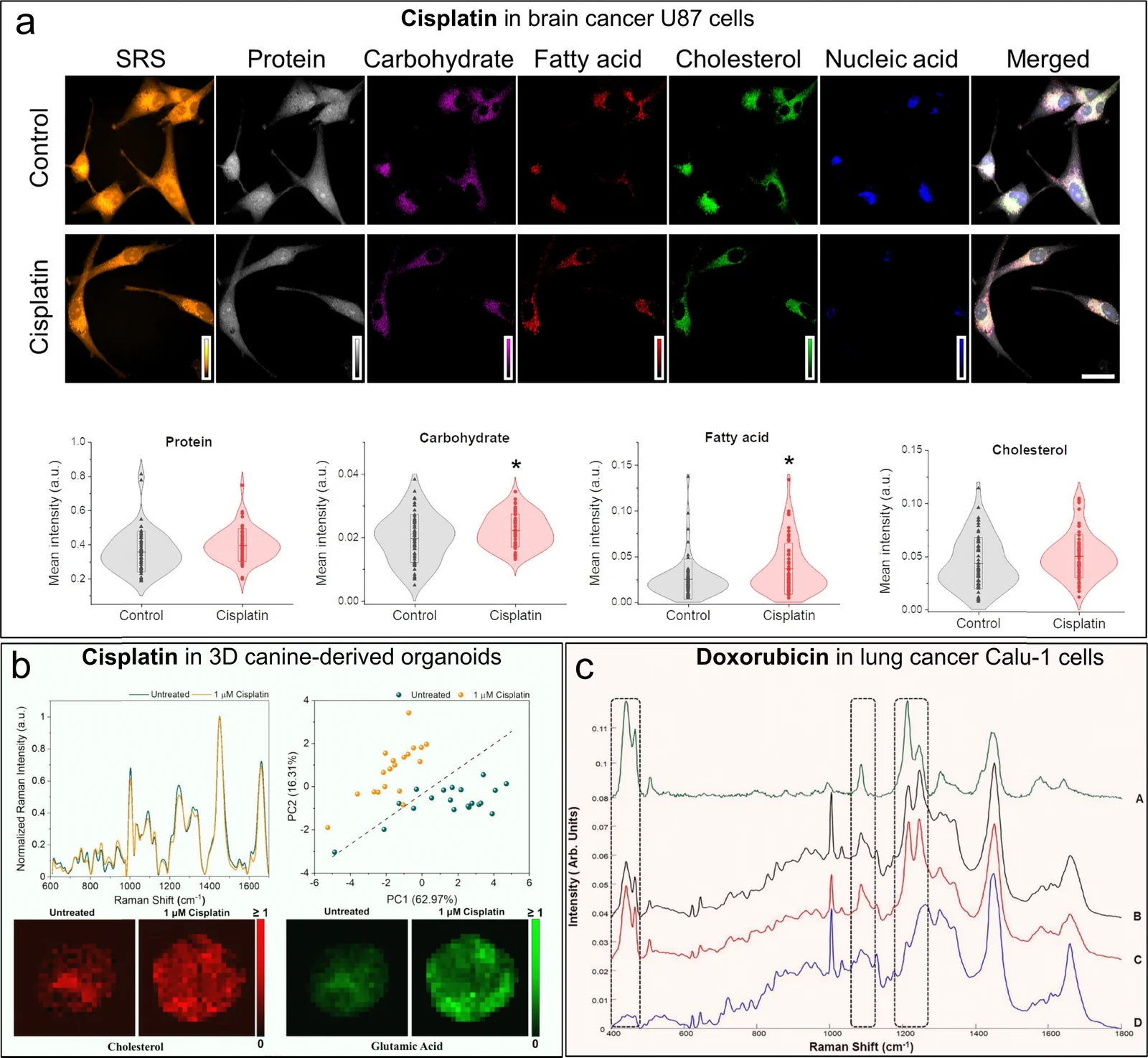
Representative examples of chemotherapy monitoring using label-free Raman-based approaches. (a) High-content hyperspectral SRS (h^2^SRS) imaging revealing cisplatin-induced metabolic reprogramming in U-87 brain cancer cells. The panels display individual subcellular maps of proteins, carbohydrates, fatty acids, cholesterol, and nucleic acids, alongside quantitative violin plots highlighting significant lipid and carbohydrate accumulation in cisplatin-treated cells (adapted from Tan *et al.*, 2023^397^ with permission from AAAS). (b) Metabolic responses to cisplatin in 3D canine-derived organoids, featuring mean Raman spectra, a PCA scatter plot with a linear SVM decision boundary differentiating treated from untreated groups, and spatial Raman maps tracking changes in cholesterol and glutamic acid relative intensities (adapted from Kothadiya *et al.*, 2024^242^ with permission from the American Chemical Society). (c) Calu-1 lung cancer cells exposed to doxorubicin: drug spectrum and compartment-resolved cellular spectra (nucleolus, nucleus, cytoplasm) with discriminant doxorubicin bands highlighted (adapted from Farhane *et al.*, 2017^293^ with permission from Wiley).

To capture the metabolic reprogramming associated with chemoresistance, Tan *et al.* (2023) developed a high-content hyperspectral stimulated Raman scattering (h^2^SRS) imaging platform. By exploiting the information-rich CH, the authors combined hyperspectral imaging with a pixel-wise LASSO unmixing algorithm to separate highly overlapping Raman signals. This approach enabled the simultaneous, label-free subcellular mapping of proteins, carbohydrates, fatty acids, cholesterol, and nucleic acids, and was validated by accurately resolving the closely overlapping C=C bands of triglycerides (1655 cm^−1^) and cholesterol (1672 cm^−1^) in the fingerprint region. Applied to cisplatin-treated U-87 glioblastoma cells and gemcitabine-treated Mia Paca2 pancreatic cancer cells, h^2^SRS revealed a marked intracellular accumulation of fatty acids and carbohydrates (U-87 glioblastoma cells under cisplatin treatment shown in Fig. 11a), a metabolic phenotype that was also observed in gemcitabine-resistant G3K cells. These findings were further confirmed by isotopic SRS imaging in the silent C–D region (2100–2300 cm^−1^), which demonstrated increased uptake of deuterated glucose-D_7_ and oleic acid-D_34_ following chemotherapy. Importantly, the resulting single-cell metabolic profiles identified therapeutically relevant metabolic dependencies. Inhibiting Raman-identified targets, including the CD36 fatty acid transporter with sulfosuccinimidyl oleate (SSO) and GLUT1 with BAY-876, significantly enhanced chemotherapy sensitivity, reducing the IC_50_ of gemcitabine in resistant G3K cells by ∼41% and that of cisplatin in U-87 cells by 51%.^397^

Expanding the application of 3D cancer models to investigate spatial tumor heterogeneity, Kothadiya *et al.* (2024) employed SR to characterize cisplatin-induced metabolic responses in 4T1 cancer cells, 3D spheroids, and canine-derived tumor organoids. Spatially resolved Raman mapping combined with PCA-SVM classification revealed early metabolic alterations following treatment, including reduced nucleic acid (783 cm^−1^), amino acid (1338 cm^−1^), and protein (1248 cm^−1^) signals, together with increased glutamic acid (878 cm^−1^) and cholesterol (700 cm^−1^) levels, shown in Fig. 11b. Importantly, depth-resolved Raman profiling from the organoid surface to the hypoxic core (−50 µm) demonstrated pronounced spatial metabolic heterogeneity. KEGG pathway enrichment analysis, derived directly from the intensities of localized Raman marker bands, including 938 cm^−1^ (citric and succinic acids) and 878 cm^−1^ (glutamic acid), revealed depth-dependent alterations in the tricarboxylic acid (TCA) cycle and d-glutamate metabolism, highlighting distinct metabolic adaptations within the organoid core.^242^

Tanwar *et al.* (2025) recently reported a DNA-origami–based hybrid nanodevice (ND) that coupled DOX with label-free vibrational imaging to monitor intracellular drug activation and response. The flat DNA sheet (∼80 × 60 nm) was coated with a short peptide that could be cut by the tumor-associated enzyme legumain; the cancer drug doxorubicin was attached right next to this cut site, and cell-penetrating peptides were added to help entry into cells. This coating made the DNA device more stable in biological conditions. The intrinsic Raman band of doxorubicin at 1206 cm^−1^ (tracked against the cellular CH_2_ band at 1440 cm^−1^) was leveraged with confocal Raman microscopy (785 nm) to map ND behavior after internalization: in legumain-high DU145 prostate cancer cells, peptide cleavage liberated doxorubicin, yielding strong nuclear and cytoplasmic 1206 cm^−1^ signal, whereas in legumain-low LNCaP cells the signal remained cytoplasmic, enabling phenotypic discrimination by Raman imaging. MALDI of cell lysates corroborated enzyme-triggered activation by detecting the leucine–doxorubicin fragment only in DU145, and viability assays showed selective ND cytotoxicity toward DU145 (IC_50_ ∼ 6.4 µM) with minimal effect on LNCaP, in contrast to indiscriminate toxicity from free doxorubicin (IC_50_ ∼ 0.35–0.42 µM). The ND also penetrated 3D DU145 spheroids more deeply than free drug, highlighting delivery advantages of the origami scaffold. Collectively, the study established a programmable, enzyme-responsive DNA-origami platform in which the therapeutic (doxorubicin) doubled as a vibrational tag at 1206 cm^−1^, providing a unified theragnostic tool to monitor doxorubicin response in 2D and 3D models.^398^

Overall, chemotherapy-monitoring studies show that Raman spectroscopy can capture both direct drug-associated signals and downstream cellular responses, including DNA damage, apoptotic remodeling, oxidative stress, and changes in protein and lipid organization. The strongest applications are those that combine drug localization with independent biological validation, thereby distinguishing pharmacokinetics from true therapy-induced response.

#### 7.1.4 Targeted therapies and new approaches

##### Targeted therapies

Targeted and pathway-oriented therapies aim to interfere with specific molecular alterations, signaling cascades, or metabolic dependencies that sustain tumor growth, survival, and treatment resistance. Cutshaw *et al.* (2024) employed SR to monitor drug-induced metabolic reprogramming in colorectal cancer cell models with different KRAS status, including KRAS-mutant HCT116 and SW837 cells and KRAS wild-type CC cells. Raman spectroscopy combined with multivariate analysis enabled discrimination between non-responsive, partially responsive, and responsive phenotypes upon treatment with cetuximab (CTX, an EGFR-targeting monoclonal antibody), sotorasib (a clinically approved KRAS inhibitor), and the MAPK pathway inhibitor trametinib (TMB). Cells were treated for 72 h and single-cell Raman maps were acquired with 2 µm spatial resolution. For CTX treatment, in the case of CTX-responsive CC cells significant decreases were observed in DNA base bands at 654, 668, and 783 cm^−1^ and in the phosphodiester backbone at 1096 cm^−1^, whereas these features remained unchanged in HCT116 cells, consistent with reduced CTX efficacy. CC cells also exhibited marked reductions in amino-acid-associated peaks, including histidine (1314 cm^−1^), phenylalanine (622, 1031, and 1583 cm^−1^), and proline (643 cm^−1^). Lipid-associated features were comparatively less affected, except for a decrease at 1173 cm^−1^, while an increase in cholesterol-related signals suggested cellular stress and apoptosis. In HCT116 cells, only a reduction in the RNA-associated band at 810 cm^−1^ was observed, reflecting a partial rather than complete resistance, in line with the distinct molecular behavior of the KRAS G13D mutation. Trametinib treatment in HCT116 cells also displayed significant changes in DNA-associated bands at 668, 783, and 1096 cm^−1^ and in a membrane lipid band at 723 cm^−1^, indicating impaired DNA synthesis and membrane integrity. At a higher dose (25 nM), these effects were substantially amplified (Fig. 12D). In contrast, subtoxic TMB treatment had minimal impact on amino-acid- (828, 858, 991 cm^−1^), fatty-acid- (958, 1173 cm^−1^), and protein- (amide III at 1248 cm^−1^) signatures, highlighting the limited efficacy of low-dose monotherapy. Notably, increases in peaks associated with tricarboxylic acid cycle intermediates and glucose metabolism (935 and 1126 cm^−1^) suggested preserved or compensatory energy metabolism under subtoxic inhibition, underscoring the potential need for combination or higher-dose strategies.^399^

**Fig. 12:**
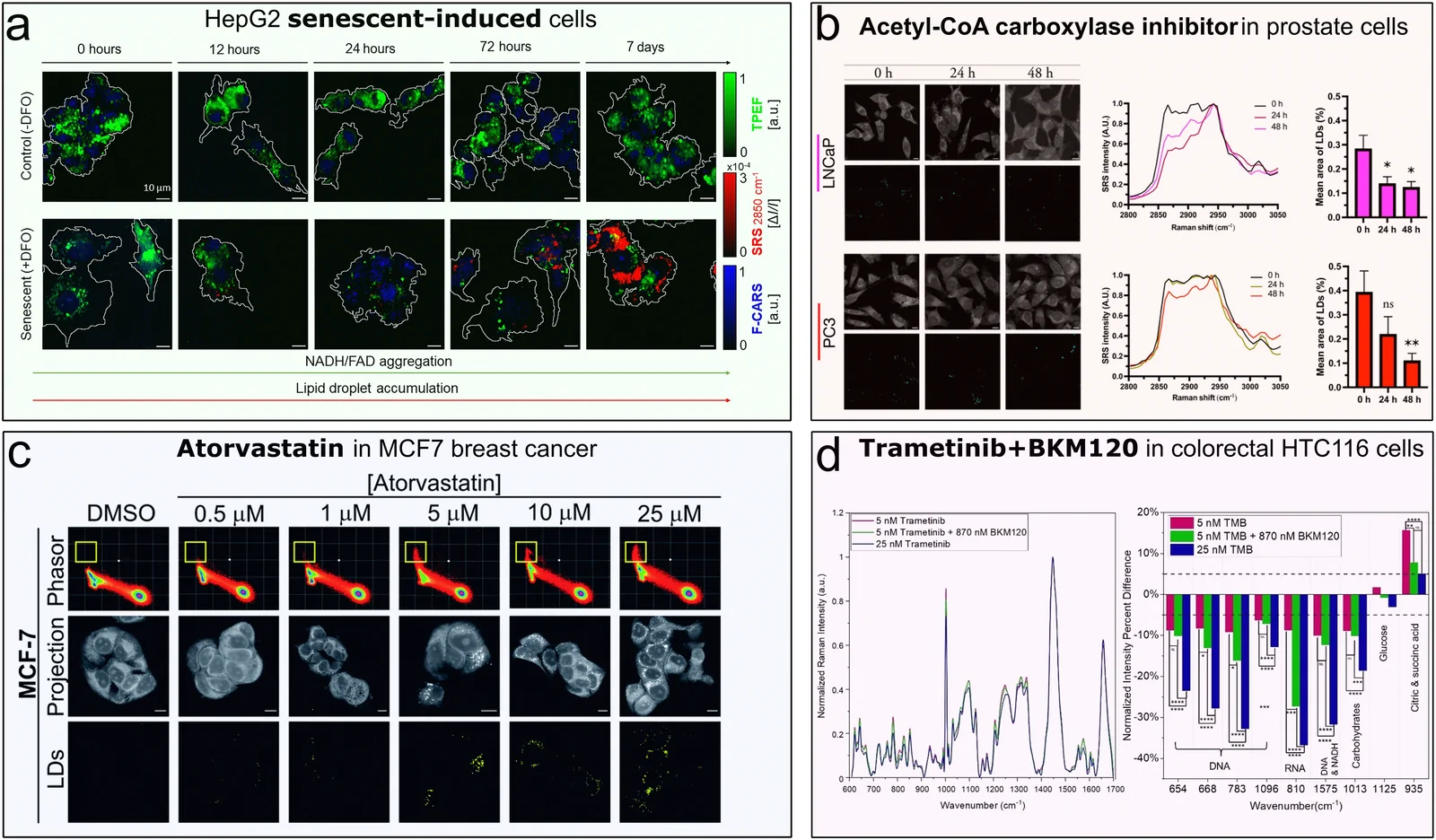
Representative Raman-based examples of targeted therapies and new monitoring approaches. (a) DFO-induced senescence in HepG2: multimodal NLO readout combining NAD(P)H/FAD TPEF, lipid SRS (2850 cm^−1^) and nuclear deoxyribose F-CARS (2970 cm^−1^), showing increased lipid signal in senescent cells (adapted from Bresci *et al.*, 2023^5^ under a Creative Commons Attribution 4.0 International License). (b) Acetyl-CoA carboxylase inhibitor in androgen-dependent (LNCaP) and castration-resistant prostate cancer cell models (PC3), including Raman spectra in the CH region from segmented LDs (adapted from Hislop *et al.*, 2022^400^ under a Creative Commons Attribution 4.0 International License). (c) Atorvastatin treatment in MCF-7 breast cancer cells. Spectral phasor plots (top row), average-intensity projections of the SRS spectral sweep (middle row), and segmented lipid-droplet (LD) images (bottom row) are shown for cells treated with increasing concentrations of atorvastatin (0.5–25 µM) for 48 h, alongside the DMSO control. Lipid droplets were identified using the yellow region of interest in the spectral phasor plots, revealing increased lipid-droplet accumulation at higher atorvastatin concentrations. Adapted from Tipping *et al*. (2022),^278^ under a Creative Commons Attribution 4.0 International License. (d) Trametinib and BKM120 in HCT116: mean spectra (*n* = 60 cells/condition) and fold-changes of selected nucleic-acid and lipid bands *versus* untreated cells (adapted from Cutshaw *et al.*, 2024^399^ with permission from Wiley).

Hislop *et al.* (2022) investigated lipid metabolic pathways in metastatic prostate cancer cells treated with 5-(tetradecyloxy)-2-furoic acid (TOFA), an inhibitor of acetyl-CoA carboxylase that blocks *de novo* lipid synthesis (DNL). Using SRS microscopy combined with k-means cluster analysis, androgen-dependent (LNCaP) and castration-resistant (PC3) cells were imaged at 2930 cm^−1^ (CH_3_, proteins), 2851 cm^−1^ (CH_2_, lipids), and through CH_2_/CH_3_ ratio maps to quantify lipid droplet (LD) content at the subcellular level. TOFA treatment (5 µM, 24–48 h) caused a pronounced reduction in LD number and area in both cancer cell lines, reflected by decreased SRS intensity at 2851 cm^−1^ and reduced CH_2_/CH_3_ ratios, while non-malignant prostate cells were unaffected. Spectral analysis of LD clusters showed reduced contributions from unsaturated lipids at 3015 cm^−1^ (=CH, triacylglycerols) and 2965 cm^−1^ (cholesteryl esters), indicating suppression of lipid biosynthesis and remodeling of intracellular lipid droplets (Fig. 12B). These findings demonstrate a strong dependence of prostate cancer cells on DNL to sustain membrane biogenesis and energy storage, with LNCaP cells showing near-complete LD depletion, whereas PC3 cells retained partial lipid stores, consistent with activation of compensatory metabolic pathways such as lipid uptake or fatty-acid recycling.^400^

Ali *et al.* (2019) combined Raman spectroscopy with live single-cell mass spectrometry to correlate tamoxifen uptake, metabolism, and biochemical response at the single-cell level.^401^ Fixed HepG2 cells were first screened by SR (fingerprint region, 600–1700 cm^−1^), and individual cells were subsequently analyzed by mass spectrometry to quantify intracellular tamoxifen (*m/z* 372.23) and 4-hydroxytamoxifen (*m/z* 388.23). Raman spectra of treated cells showed decreased intensity of the cytochrome band at 752 cm^−1^ and protein-related bands at 1545 cm^−1^ (amide II) and 1651 cm^−1^ (amide I), alongside increased aromatic and nucleic-acid-associated features at 1001 cm^−1^ (phenylalanine), 1236 cm^−1^ (nucleic acids/amide III), and 1601 cm^−1^, indicating metabolic and stress-related responses. Lipid–protein contributions at 1445 cm^−1^ were also altered, consistent with membrane remodeling. Supervised PLS-DA reliably classified treated *versus* untreated cells. Correlation with single-cell MS showed that increased intracellular 4-OHT levels were associated with higher intensities of nucleic-acid–related Raman bands and reduced amide intensity, indicating enhanced metabolic activity coupled to a stronger molecular response to tamoxifen.

Du *et al.* (2020) applied Raman-guided single-cell metabolomics to identify metabolic vulnerabilities in patient-derived BRAF-mutant melanoma cells and to monitor their response to pharmacological inhibition of stearoyl-CoA desaturase-1 (SCD1). Single-cell SR was first used to capture global biochemical differences, revealing that cellular de-differentiation was associated with a progressive decrease in the lipid-to-protein balance, reflected by reduced intensity of the lipid CH_2_ band at 2845 cm^−1^ relative to the protein CH_3_ band at 2940 cm^−1^. This spectral metric enabled stratification of melanoma phenotypes with different metabolic dependencies. To validate and spatially resolve these findings, the authors employed SRS imaging at the two aforementioned bands (2845 cm^−1^ and 2940 cm^−1^), generating CH_2_/CH_3_ ratio maps that confirmed a lower lipid content in de-differentiated cells. While bulk lipid levels decreased with de-differentiation, hyperspectral SRS revealed that mesenchymal cells accumulated lipid droplets enriched in unsaturated lipids, characterized by an increased =C–H stretching band at 3022 cm^−1^ and enhanced cholesteryl ester features at 2957–2997 cm^−1^. Based on this Raman-defined lipid phenotype, the authors targeted stearoyl-CoA desaturase-1 (SCD1), a key enzyme controlling lipid unsaturation. Pharmacological SCD1 inhibition induced a selective decrease of the 3022 cm^−1^ unsaturation signal in lipid droplets, followed by loss of cell viability and apoptosis in mesenchymal melanoma cells, while differentiated cells were less affected.^402^

Tipping *et al.* (2022) explored the effects of atorvastatin and rosuvastatin on breast cancer cells, using spectral phasor analysis with SRS microscopy to assess lipid accumulation. Statins, which inhibit HMG-CoA reductase (3-hydroxy-3-methylglutaryl-coenzyme A reductase) in the mevalonate pathway, were studied in three breast cancer cell lines with distinct estrogen receptor-α (ERα) profiles: MCF-7 (ERα+), SK-BR-3 (ERα−), and MDA-MB-231 (ERα−). SRS imaging revealed lipid droplet distributions and lipid/protein signatures at specific Raman shifts (2930 cm^−1^ for proteins and 2851, 2880, and 3010 cm^−1^ for lipids). Atorvastatin treatment led to increased lipid content in MCF-7 (see Fig. 12C) and SK-BR-3 cells, particularly at concentrations above 5 µM, while MDA-MB-231 cells showed no significant lipid changes. Interestingly, despite statins' inhibition of cholesterol biosynthesis, no notable changes were found in the lipid droplet composition ratio (3015 cm^−1^/2965 cm^−1^), which remained consistent across all treatments, indicating low cholesterol ester content in lipid droplets. The study also examined whether lipid accumulation resulted from *de novo* synthesis or exogenous lipid uptake, showing that atorvastatin treatment in serum-free media led to significant lipid accumulation in MCF-7 cells (Fig. 12C), whereas rosuvastatin had a weaker effect. Cell viability assays indicated that atorvastatin produced a more pronounced effect on lipid metabolism and cell viability than rosuvastatin. Additionally, statin sensitivity appeared independent of ERα status, with MCF-7 cells showing the greatest lipid accumulation and resilience to statin treatment, while MDA-MB-231 cells demonstrated higher toxicity but minimal lipid accumulation. These findings suggest that lipid accumulation might be linked to statin resistance in breast cancer cells.^278^

##### Senescence

Recently, Bresci *et al.* exploited multimodal Raman, CARS, SRS and TPEF microscopy, combined with three-dimensional QPI, to detect the early molecular, metabolic and morphological rearrangements of therapy-induced senescence (TIS) in human cancer cells, upon both chemical therapy and radiation therapy.^5^ The longitudinal study delivered a chronological picture of how mitochondrial co-enzymes, NAD(P)H and FAD, lipid droplets, and cell dimensions rearranged spatially in TIS cancer cells through the first 7 days after chemical treatment, with unique patterns setting this phenotype apart from normally proliferating cell states (Fig. 12A). The group developed their TIS screening strategy on HepG2 cells undergoing senescence induction *via* a validated biological model using deferoxamine,^223^ using a large sample size of ∼100 cells for each of the 5 time points, for each condition (*i.e.*, TIS and control). Then, they validated their results on standard radiotherapy (10 Gy dose of γ radiation on HepG2s) with a follow-up period of 20 days. Results were consistent across other human cell lines, such as TPC-1 thyroid carcinoma cells, as well as other common chemotherapeutic agents, like doxorubicin. Their TIS optical screening strategy used single-color SRS and CARS images at the Raman modes of proteins and lipids, coupled with TPEF images showing the varying distribution of mitochondrial cofactors and 3D QPI maps of evolving cell morphology. Hence, dimensionality reduction steps typical of hyperspectral image analysis were not considered. Instead, metrics for downstream statistics were derived as signal distribution and signal intensity patterns, exploiting the quantitative nature of CRS and TPEF-based chemical images and QPI morphological images. Due to the still enigmatic nature of senescence, with currently no clear standard set of hallmarks to identify this state of cell cycle arrest across therapy types and cell phenotypes, and because of the central role TIS cells and their secretome play in cancer recurrence, this work paved the way for expedited cancer therapy screening in the fight against senescence-driven resistance.

##### Energy-based and locoregional therapies

Beyond pharmacological treatments, Raman spectroscopy has also been explored to monitor therapies based on localized physical or photoinduced tumor damage, such as photodynamic therapy and thermal ablation. In these settings, Raman readouts can capture biochemical signatures of cell death, protein denaturation, nucleic-acid damage, and tissue remodeling. In 2018, Bhattacharjee *et al.* evaluated biochemical effects of photodynamic therapy (PDT) simulation *in vivo* in live rat breast tumors using RS. Spectra were recorded using a 785 nm incident laser wavelength, with three accumulations of 10 seconds and 10 spectra collected per sample in pretherapy, 24 hours after photosensitizer injection, and 24 hours post-photodynamic therapy (post-PDT) in a total of six young, virgin Sprague–Dawley female rats. The photosensitizer chlorin photodithazine (8 mg kg^−1^) from *Spirulina platensis* was administered intraperitoneally to each animal. After 24 hours, animals were anesthetized and treated with 100 J cm^−2^ light at 670 nm using a diode laser and optical fiber diffuser at a fluence rate of 100 mW cm^−2^. Multivariate principal component-linear discriminant analysis revealed that peaks corresponding to amino acids (1002, 1209, 1604, 1618 cm^−1^), nucleic acids (747, 1421 cm^−1^), and proteins (1600 cm^−1^), indicated significant biochemical changes post-PDT. To classify groups, PC-LDA was applied using PCs 1–10, achieving 82% classification efficiency for untreated tumors (T), 70% for post-photosensitizer injection (P), and 87% for post-PDT. Misclassifications primarily occurred between P and PDT (13–20%), suggesting biochemical similarities, possibly due to uneven photosensitizer distribution or minor dark toxicity effects. Importantly, complete separation was observed between pretherapy (T) and post-PDT. Specifically, there was a noticeable decrease in amino acid bands (1546, 1562, 1582, 1604, and 1620 cm^−1^), corresponding to tryptophan and phenylalanine, reflecting protein breakdown and cellular damage. Additionally, nucleic acid bands (752 and 788 cm^−1^) showed a reduction, likely due to decreased DNA integrity and replication caused by PDT-induced cell death. In contrast, an increase in collagen-associated protein bands (874, 1452, and 1668 cm^−1^) was observed, suggesting collagen remodeling as part of the tumor destruction and repair processes.

Song *et al.* (2020) combined Raman spectroscopy with PCA-LDA to evaluate the biochemical mechanisms of lung cancer microwave ablation (MWA). The study successfully differentiated between normal, malignant, and ablated tissues by tracking specific spectral markers linked to thermal protein denaturation and lipid degradation. Notably, ablated tissues exhibited a relative increase in peak intensities at 1665 cm^−1^ (amide I), 1454 cm^−1^ (CH_2_ deformation), and 1248 cm^−1^ (amide III) compared to untreated tumors. These spectral shifts, attributed to thermal coagulation and tissue dehydration, enabled the precise delineation of ablation margins and therapeutic efficacy with high classification accuracy.^353^

Across targeted, metabolism-oriented, senescence-inducing, and locoregional therapies, Raman-based monitoring highlights a common theme: treatment response is often reflected less by a single diagnostic peak than by coordinated remodeling of metabolic, structural, and spatial features. In this context, Raman imaging is particularly valuable because it can connect pathway-specific perturbations with phenotypic consequences such as lipid-droplet accumulation, metabolic rewiring, senescence, thermal damage, or residual viable tumor.

### 7.2 In-clinic tracking of anticancer therapy response

Clinical applications shift the emphasis from mechanistic exploration to actionable decision-making. In this setting, Raman spectroscopy must provide information rapidly, reproducibly, and in a form that can be integrated into surgical, pathological, or longitudinal monitoring workflows.

#### 7.2.1 Intraoperative assessment of tumor resection margins

Two approaches are being pursued for intraoperative real-time or near-real-time tracking: *in vivo* measurements and *ex vivo* measurements on tissue biopsies. The use of *in vivo* Raman spectroscopy in patients has been primarily effective for the real-time diagnosis of cancerous tissues in head and neck,^15^ colon,^403^ breast,^404^ lung,^405^ brain,^406^ skin,^407^ and other cancers.^408^ In the case of therapy monitoring, surgery is now the most common treatment investigated *via* Raman spectroscopy in clinical studies, particularly for *in vivo* intraoperative characterization of tumor resection margins.^409,410^ For example, Jermyn *et al.* (2015) demonstrated the potential of Raman spectroscopy for intraoperative brain cancer detection using a handheld fiber-optic probe with a 785 nm laser. This probe provided rapid, real-time, *in vivo* measurements of glioma tissues during surgery, with a spectral resolution of 1.6–2.1 cm^−1^ and the ability to sample tissue at a depth of ∼1 mm. They identified increased lipidic content (700 and 1142 cm^−1^) in normal brain tissue, while higher nucleic acid content was observed (1540–1645 cm^−1^) in cancerous tissue. Using a boosted trees machine learning algorithm, they classified tissues with 92% accuracy, achieving a sensitivity of 93% and specificity of 91% for detecting invasive and dense cancer cells, integrating this protocol into the neurosurgical workflow to guide tumor resections. Similarly, Aaboubout *et al.* (2023) used high-wavenumber Raman spectroscopic thin fiber to differentiate oral cavity squamous cell carcinoma (OCSCC) from healthy tissue by analyzing (1) water concentration, calculated from the 3350–3550 cm^−1^ region, where tumor tissue exhibited higher water content; (2) lipid-to-protein ratio, determined from the Raman signal intensity ratio of the 2852–2884 cm^−1^ and 2910–2966 cm^−1^ bands, with tumors showing a lower ratio indicative of reduced lipid content; and (3) Raman-to-autofluorescence (Raman/AF) signal ratio in the 3350–3550 cm^−1^ region, where tumors exhibited lower autofluorescence.^411^ In this line, some of the authors established a clinical workflow for *in vivo* guided surgery in head and neck cancer.^15^ More recently, Calvarese *et al.* developed a multimodal CARS/TPEF/SHG endomicroscope that integrates femtosecond laser ablation. This cutting-edge technology, used to monitor the laser ablation treatment *in vivo* during surgery, was validated in a clinical trial involving 15 patients, achieving AI-based tumor tissue diagnosis with 88% sensitivity and 95% specificity. The system enables machine learning-driven, image-guided selective tissue removal. To the best of our knowledge, this is the first real-time *in vivo* monitoring of laser ablation treatment in human patients.^132^

The use of a fiber-optic Raman probe for intraoperative guidance is a promising method for real-time tumor assessment. However, it is not the only Raman-based approach available for intraoperative evaluation. The rapid analysis of resected unsliced and unstained tumor tissue, compared to traditional histology and H&E staining, not only improves surgical efficiency but also reduces the likelihood of leaving malignant tissue at the resection margins, leading to better surgical outcomes. In addition, healthy tissue can be spared, which in turn reduces the risk of complications and long-term functional disabilities. Building on this concept, Hollon *et al.* introduced a clinical SRS microscope, initially validated on 101 neurosurgical patients. This system, commercially available as INVENIO, demonstrated rapid *ex vivo* detection of brain tumor infiltration in fresh, unprocessed human tissue biopsies.^148^ As previously introduced in Section 3.2 (Raman spectroscopy in biomedicine), in 2020, they incorporated deep neural networks trained on over 2.5 million images, achieving predictions of brain tumor margins (*ex vivo*) in the operating room in under 150 seconds, significantly faster than conventional frozen section methods (*e.g.*, 20–30 minutes), with an overall accuracy of 94.6%.^13^ Furthermore, they developed FastGlioma, an AI-based model. Pretrained using a large dataset of approximately four million images, FastGlioma was fine-tuned to output a normalized score reflecting the extent of tumor infiltration within whole-slide optical images, significantly improving the detection of tumor infiltration during surgery.^14^

Intraoperative margin assessment currently represents the most mature clinical scenario for Raman-based cancer therapy monitoring, because the clinical question is well defined, the decision is immediate, and the optical readout can be directly compared with histopathology. Its main challenge is no longer proof of contrast, but robust integration into time-critical surgical workflows.

#### 7.2.2 Biofluid-based monitoring

The levels of biomolecules in blood, urine, and tissue samples show significant differences between cancer patients and healthy individuals.^412^ This, combined with the routine collection and biobanking of biofluids from cancer patients by clinical centers, has opened the door to a growing field of research focused on using Raman spectroscopy as a possible liquid biopsy tool for cancer detection. On the other hand, the possibility to use spectroscopic methods for biofluid analysis to gain information on the response to treatment remains underexplored.

De la Torre-Gutiérrez *et al.* (2022) employed Raman spectroscopy in blood serum samples to monitor chemotherapy response in breast cancer patients, revealing key spectral shifts linked to treatment efficacy. The study identified significant decreases in the Raman peak of glutathione at 450 cm^−1^ in patients responding positively to chemotherapy, indicating a reduction in oxidative stress and suggesting that glutathione could serve as a biomarker for chemotherapy effectiveness. Conversely, non-responders exhibited spectral profiles within the cancerous group, with no significant change in the glutathione signal, reflecting persistent oxidative stress. Additionally, peaks associated with amino acids (*e.g.*, phenylalanine at 1003 cm^−1^) and phospholipids (at 1083 cm^−1^) were altered.^413^

Giamougiannis *et al.* (2021) analyzed plasma, serum, and urine samples from 116 ovarian cancer patients. By comparing the spectral features of patients receiving neoadjuvant chemotherapy (NACT) with those of untreated patients, the study highlighted chemotherapy-induced metabolic changes, particularly lipid alterations, visible in the plasma and serum spectra. The increase in lipid-related peaks at 1450 cm^−1^ and 1750 cm^−1^ suggests chemotherapy-induced lipid accumulation and metabolic reprogramming, particularly a shift toward lipid metabolism in response to therapy. In contrast, untreated cancer patients exhibited a higher intensity of peaks at 880 cm^−1^ and 950 cm^−1^, associated with carbohydrates, and at 1000 cm^−1^, 1380 cm^−1^, and 1680 cm^−1^, related to amino acids (*e.g.*, phenylalanine, tryptophan, and glutamine). These findings reflect the active proliferative state of the tumor, characterized by enhanced carbohydrate metabolism for energy production and increased amino acid synthesis for protein biosynthesis.^414^

In a more recent example, Morasso *et al.*^237^ demonstrated a clinically oriented Raman liquid biopsy approach for therapy monitoring by measuring dried blood plasma from 32 colorectal cancer (CRC) patients (plus controls: 31 healthy, 37 IBD, 20 diverticular disease) and then following CRC patients after surgical resection (6 days, 30 days, 6 months). Using 785-nm Raman on 4 µL plasma drops dried on CaF_2_, they acquired spectra in the fingerprint region (multiple accumulations; three positions per sample, selected on the outer ring of the dried drop to reduce inhomogeneity), and applied baseline correction. They identified a compact panel of six discriminant Raman bands: 505 cm^−1^ (protein disulfide bridges), 760 cm^−1^ (tryptophan), 826 cm^−1^ (nucleic-acid O–P–O), 1003 cm^−1^ (phenylalanine), 1155 cm^−1^ (carotenoids), and 1420 cm^−1^ (lipids). CRC shows a characteristic shift *versus* non-CRC conditions, notably higher 505/760/826 cm^−1^ and reduced carotenoids, while inflammatory cohorts show a different pattern (*e.g.*, reduced phenylalanine), supporting that cancer-related changes are not simply inflammation-driven. A logistic regression model built on these six features (leave-one-patient-out) achieved 87.5% diagnostic accuracy. For therapy monitoring, the longitudinal results showed CRC-associated elevations (*e.g.*, disulfides, nucleic-acid O–P–O, lipids) progressively decreased after surgery, while carotenoids rise toward healthy-like levels, with the overall profile approaching controls by ∼6 months, and the classifier performance correspondingly drops when tested on 6-month post-surgery samples (47.8% accuracy), consistent with loss of the preoperative cancer signature. From a future perspective, cerumen analysis represents a promising candidate for the integration of Raman-based approaches into point-of-care monitoring of therapy response in HNSCC. Although it has thus far been primarily explored as a diagnostic tool, both label-free^415^ and SERS-based^416^ strategies could expand its application toward longitudinal treatment monitoring.

Biofluid-based Raman monitoring offers a complementary route to tissue-based approaches by enabling minimally invasive, repeatable sampling during therapy. Its translational potential will depend on whether spectral signatures can be linked to specific biological mechanisms and validated against longitudinal clinical endpoints, rather than used only as statistical classifiers of disease or treatment status.

## 8 Raman fingerprint of cancer therapy response

This section moves from study-by-study description to cross-treatment interpretation, asking whether recurrent Raman signatures of response and resistance can be identified across therapeutic modalities. The reviewed literature confirms that Raman spectroscopy is a powerful tool for decoding the complex biological heterogeneity of cancer therapy responses. This section provides an integrated discussion based on therapy response monitoring and prediction studies covered in Sections 4, 6, 7.1, and 7.2. It summarizes key findings using a Sankey diagram (Fig. 13a and Table S1) and a representative figure for each treatment evaluated (Fig. 13b). The aim of this section is to consolidate the key Raman shifts associated with therapy response monitoring and prediction, providing guidance on the types of Raman-based results that may be relevant for future studies.

**Fig. 13:**
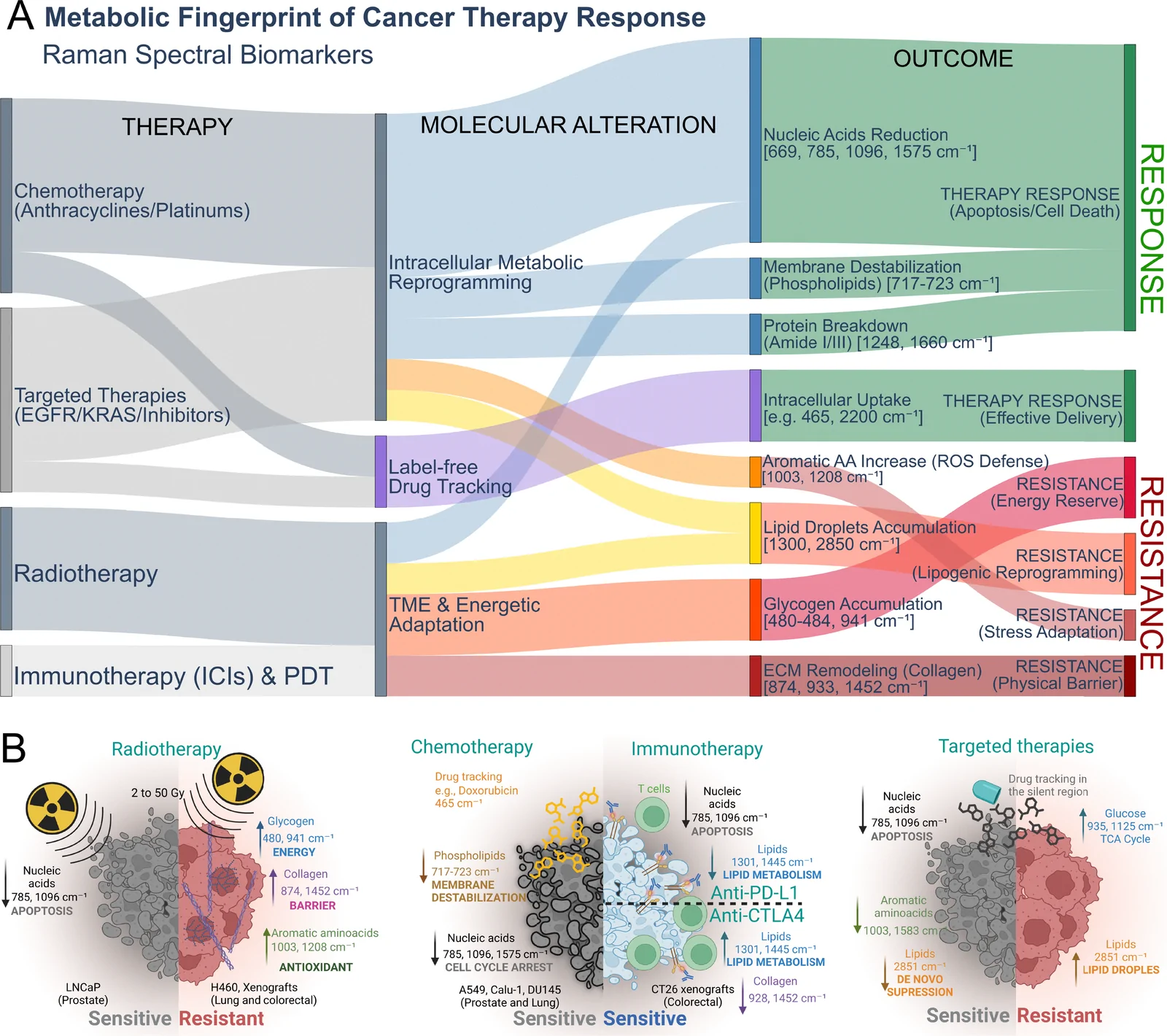
Metabolic fingerprint of cancer therapy response. (a) Sankey diagram illustrating the metabolic pathways affected by various cancer therapies, including chemotherapy (anthracyclines/platinums), targeted therapies (*e.g.*, EGFR/KRAS inhibitors), radiotherapy, and immunotherapy (ICIs & PDT). Each therapy is associated with distinct molecular alterations in the tumor microenvironment (TME), such as intracellular metabolic reprogramming, membrane destabilization, protein breakdown, and lipid metabolism. Raman spectral biomarkers are linked to these pathways, showing therapy-induced changes (*e.g.*, apoptosis, effective drug delivery) and resistance mechanisms (*e.g.*, energy reserve, lipogenic reprogramming). (b) Schematic representation showing the cellular and tissue-level effects of the therapies, highlighting the differences between sensitive and resistant models. The information used for the Sankey diagram can be found in Table S1.

Integrating data from radiotherapy, chemotherapy, immunotherapy, and targeted therapies suggests recurrent biochemical patterns associated with responsive and resistant phenotypes; the underlying studies, including the biological models, therapeutic contexts, and major spectral alterations reported, are summarized in Table S1. This spectral fingerprint goes beyond simple chemical bond identification, providing a direct readout of the cellular adaptations required for survival under therapeutic stress. Across various treatments, successful responses are consistently marked by the breakdown of macromolecular integrity, whereas resistant or partially responsive models tend to preserve these biochemical features or show attenuated changes (Fig. 13). In responsive samples, this is evident in the reduction of nucleic acid signals, notably the O-P-O backbone bands at 785 cm^−1^ and 1096 cm^−1^, a trend observed from, for example, lung cells treated with doxorubicin to colorectal cells responding to EGFR inhibition.^242,244,293,394^ Although this spectral feature is shared across treatment modalities, the intrinsic apoptosis-related processes differ substantially.^417^ Consequently, the reduction of DNA-associated bands represents a common spectral endpoint reached through distinct molecular pathways. In chemotherapy, it mainly reflects direct drug-induced alterations of DNA structure,^418^ whereas targeted therapies predominantly reduce these signals through cell-cycle arrest and subsequent apoptotic remodeling.^24^ In radiotherapy, the rapid loss of nucleic acid bands is associated with extensive radiation-induced DNA damage,^83,419^ while in immunotherapy it develops with the delayed kinetics of T-cell-mediated cytotoxicity.^420^ These mechanistic differences suggest that Raman spectroscopy may provide information not only on treatment response but also on the biological processes driving it. As shown in Fig. 13, this reduction is often coupled with membrane destabilization, signaled by changes in phospholipid bands around 717–723 cm^−1^, and protein denaturation, which are common spectral indicators consistent with apoptosis-associated irreversible damage.

Conversely, therapeutic resistance is not merely the absence of cell death, but a state of active cellular reprogramming. A central pillar of this adaptation is metabolic rewiring, although the behavior of specific biomolecules, such as glycogen (detected at 941 cm^−1^), remains highly context-dependent. For instance, while early *in vitro* studies linked glycogen accumulation to a survival mechanism in response to radiation,^280,383^ subsequent *in vivo* xenograft models suggested that this increase may instead represent an acute stress response in radiosensitive tumors.^388^ In targeted treatments, resistance is frequently based on the preservation or enhancement of oxidative metabolism. For example, cells evading subtoxic MAPK inhibition sustain TCA cycle activity, spectrally detectable *via* C–C stretching bands at 935 and 1126 cm^−1^, to bypass drug-induced metabolic bottlenecks.^399^

Lipid dynamics reveal an interesting contrast across therapeutic strategies. In chemotherapy and radiotherapy, lipid droplet accumulation, readily visualized by CRS through the strong CH_2_ stretching band at 2850 cm^−1^, is commonly associated with therapy-induced senescence^5^ and membrane remodeling, accompanied by alterations in phospholipid-associated bands around 717–723 cm^−1^.^242,383,399^ In contrast, under targeted therapies, lipid accumulation often reflects an adaptive survival mechanism rather than cellular damage. Resistant cells exposed to metabolic inhibitors, such as stearoyl-CoA desaturase-1 (SCD1) inhibitors or statins, enhance *de novo* lipogenesis, leading to the accumulation of unsaturated fatty acids and lipid droplets that help sustain membrane homeostasis and meet the energetic demands imposed by therapeutic stress.^402^

The TME also plays a crucial role in therapeutic outcomes. Raman mapping of xenografts has shown that radiation-resistant tumors often possess a collagen-rich barrier (874, 933, 1452 cm^−1^) that protects the tumor nest.^12,388^ A similar phenomenon is seen in immunotherapy, where resistance to anti-CTLA-4 checkpoint blockade is associated with collagen retention, whereas successful treatment leads to extracellular matrix degradation.^394^ Furthermore, Raman spectroscopy uniquely enables *in situ*, label-free pharmacokinetics by tracking the subcellular localization of drugs, such as doxorubicin *via* its intrinsic 465 cm^−1^ signal or targeted therapies utilizing silent-region tags, unavailable to traditional pathology.^240,396^ By simultaneously correlating the physical localization of therapeutics with target degradation (*e.g.*, DNA backbone collapse at 785 cm^−1^) and adaptive lipid/collagen signatures, Raman provides a holistic analytical platform for predicting therapeutic efficacy and identifying novel resistance mechanisms.

## 9 Challenges and future perspectives

### 9.1 Current limitations and challenges in clinical Raman spectroscopy for therapy response

The previous sections have highlighted the potential of Raman spectroscopy for monitoring and predicting cancer therapy response. Nevertheless, several challenges continue to limit its widespread clinical translation. These challenges can be grouped into four main domains: (1) technological constraints of Raman systems, (2) data transferability and standardization, (3) limitations of current preclinical validation models, and (4) regulatory and clinical integration barriers.

#### 9.1.1 Technological constraints of Raman systems

The intrinsically weak Raman scattering efficiency of conventional spontaneous Raman implementations remains a limitation, particularly for rapid imaging applications. Long acquisition times restrict throughput and limit the feasibility of real-time, spatially resolved mapping *in vivo*. Thus, Mo *et al.* developed a dual-modal, label-free Raman imaging approach, by combining bright-field images with SR maps. This method enhances imaging speed by up to 65 times compared to traditional SR techniques, while still producing high-quality “virtual staining”.^421^ More recently, a study by Raj *et al.* evaluated the use of compressive sensing (CS) in Raman spectroscopy, aiming to reduce acquisition time and hardware complexity. By collecting sparse spectral data and reconstructing missing information computationally, CS enabled faster measurements with minimal loss of accuracy.^422^ Nevertheless, whole-tumor mapping with sufficient depth, speed, and signal-to-noise ratio remains technically demanding. Conversely, advances in accelerated acquisition schemes and broadband coherent Raman techniques are progressively expanding the capabilities of Raman-based imaging.

As mentioned in Section 7.2.1, coherent Raman modalities, particularly clinical SRS microscopes such as INVENIO, have demonstrated impressive performance in rapid intraoperative virtual histology. However, their broader clinical implementation is constrained by bulky laser architectures and rigid optical configurations. For longitudinal *in vivo* monitoring, especially outside controlled surgical environments, compact and fiber-based implementations are required. The next technological milestone lies in translating ultrafast coherent Raman systems into portable, flexible and possibly low-cost platforms that enable repeated, spatially distributed measurements in accessible tumor sites such as head and neck or skin malignancies. The greatest challenges in this pathway lie in (i) the development of cheap, small-footprint and rugged (*i.e.* permanently aligned and insensitive to thermal fluctuations or mechanical stress) laser sources and (ii) the integration of compact detection systems (*e.g.*, spectrometers and lock-in amplifiers) with reliable data-analysis pipelines, including both pre-processing and post-processing, capable of removing noise while preserving the underlying biochemical information and avoiding the introduction of processing artifacts.

Depth-resolved chemical profiling represents another unresolved challenge. From a research point of view, the evaluation of 3D models allows the study of tumor architecture as a whole. Nevertheless, there are still limitations in accurately mapping the internal chemistry of tumors during treatment. In this context, Raman-based approaches such as SORS and SESORS are emerging to provide label-free internal chemical maps (see Section 3.2.1. “Modalities”).^423^ In parallel, Troncoso-Afonso *et al.* advanced the SERS field by creating 3D printed SERS-active hydrogel pillars within a bioprinted breast cancer model, enabling real-time tracking of drug dynamics in both tumor and stromal compartments.^424^

#### 9.1.2 Data transferability and standardization

Perhaps the most critical translational barrier to clinical adoption of Raman spectroscopy is the lack of inter-instrument reproducibility. Spectral variations arising from differences in laser wavelength, detector response, optical alignment, and preprocessing pipelines frequently impair cross-center comparability and undermine machine-learning model robustness.

Although statistical harmonization methods exist, many studies remain limited by small datasets and narrow cohorts; Krafft *et al.* therefore call for large, high-throughput efforts to build extensive datasets and robust classifiers.^425^ This makes large, multi-center trials essential, yet difficult to organize because subtle optical differences persist and fully standardized spectral normalization is still lacking. Recently, Blake *et al.* showed that using the same spectrometer model (a Renishaw compact benchtop Raman imaging system for biomedical use) enabled result transfer across three centers, but this is only a partial solution.^426^ To further improve portability of Raman-based cancer classification, interest is growing in deep learning, including foundation models trained on large, diverse datasets.^14^ In the context of the Raman4Clinics COST ACTION project, Guo *et al.* (2020) compared 35 Raman spectrometers across 15 European institutions and found substantial variability in peak positions, intensities, widths, and noise characteristics across spectrometers, highlighting the need for standardized calibration to ensure reproducibility.^427^ They advocated built-in calibration routines, access to raw data, and open data sharing. In the same framework, Fornasaro *et al.* (2020) reported the first large-scale interlaboratory study on quantitative SERS, involving 15 laboratories and 44 researchers, and proposed a community-driven methodology to assess reproducibility and trueness as an important step toward SERS protocol standardization.^428^ More recently, the COST Action Reliability4Photonics (CA25149) was approved to further promote standardization and harmonization of Raman and other label-free photonic technologies, including measurement protocols, data-analysis pipelines, metadata, and shared data infrastructures. Overall, current clinical Raman research increasingly prioritizes harmonized analysis/interpretation workflows and FAIR-aligned open repositories to enable AI-driven model development and advanced re-analysis.^185^ In line with these efforts, community-driven open-source initiatives are also emerging to facilitate the implementation of FAIR principles in Raman spectroscopy by providing standardized data structures, metadata organization, and interoperable analysis pipelines, thereby promoting reproducibility and data sharing across laboratories. FAIRaman is one recent example of this approach.^429^

#### 9.1.3 Limitations of current preclinical validation models

A diverse landscape of preclinical cancer models is available, ranging from conventional 2D cultures to xenografts and advanced three-dimensional systems. These platforms have enabled substantial progress in Raman-based mechanistic investigations. However, challenges remain when such models are used to establish predictive, patient-relevant therapy-response biomarkers. Standardized commercial cell lines, although experimentally reproducible, do not fully recapitulate inter-patient heterogeneity. In the context of therapy prediction, patient-specific models refer to systems directly derived from individual tumor tissues, preserving molecular and metabolic complexity. While patient-derived organoids and tumor slices represent important advances, the consistent integration of a functional immune microenvironment remains limited. Given the growing relevance of immunotherapy and immune-mediated resistance, this represents a non-trivial translational gap.

Moreover, advanced 3D models often involve substantial logistical demands. Organoid generation requires specialized growth conditions and may necessitate extended culture periods before reaching biological maturity. Such timelines may restrict rapid translational feedback and reduce applicability in time-sensitive decision-making scenarios. In line with these limitations, new approaches for therapy prediction, such as the patient-derived tumor organoids (PDOs), have demonstrated low sensitivity to certain drugs and require extended time periods to form tissues (approximately 2–8 weeks).^430^ New 3D models, such as patient-derived tumor slices,^431^ are now introduced to evaluate therapy simulation, with expected good predictability and a reduction of therapeutic failure rates.^432,433^ As a result, although current models are powerful tools for biomarker discovery, additional refinement is required to strengthen their predictive alignment with clinical therapy outcomes. However, ethical and regulatory considerations further complicate the integration of patient-derived and genetically modified systems into standardized translational pipelines.

#### 9.1.4 Regulatory and clinical integration: a roadmap from concept to clinic

Even when technical feasibility and biological relevance have been demonstrated, regulatory approval and clinical workflow integration remain major barriers to translation.^434^ As illustrated in Fig. 14, Raman spectroscopy in oncology can be applied through two complementary paradigms: baseline prediction and longitudinal monitoring. In the baseline approach, AI-assisted Raman analysis of initial liquid or solid biopsies supports therapy selection at diagnosis. In contrast, longitudinal monitoring uses serial liquid biopsies and patient-derived models (*e.g.*, organoids, tumor slices, and cells) to assess treatment response over time, enabling early identification of responders and non-responders, timely therapeutic adjustments, and improved understanding of resistance mechanisms. This transition toward continuous molecular profiling and deep phenotyping is a key step toward precision oncology.

**Fig. 14:**
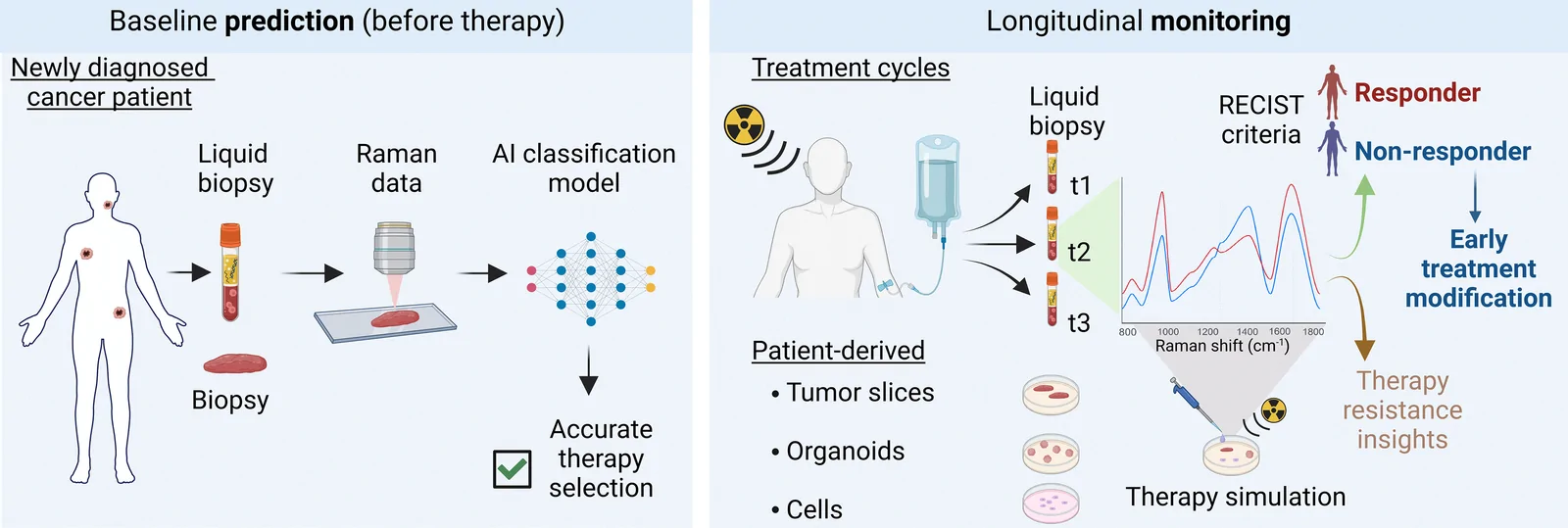
Proposed framework for AI-assisted Raman spectroscopy in oncology. Baseline therapy prediction using initial biopsies (left) with longitudinal monitoring during treatment cycles to differentiate clinical responders from non-responders and guide early treatment modifications (right).

From a regulatory standpoint, Raman-based systems intended for clinical decision support are classified as medical devices and must comply with national and international regulatory frameworks. In the United States, approval pathways are governed by the U.S. Food and Drug Administration (FDA), typically under the 510(k), *De Novo*, or Premarket Approval (PMA) processes, depending on risk classification. In the European Union, compliance with the Medical Device Regulation (EU MDR 2017/745) is required, including conformity assessment, clinical evaluation reports, and post-market surveillance obligations. These frameworks demand rigorous demonstration of analytical validity, clinical validity, and clinical utility. For Raman-based therapy monitoring, this requires (i) standardized acquisition protocols, (ii) reproducible measurements across instruments and clinical sites, (iii) a clearly defined intended use and risk classification, (iv) rigorous validation against established clinical endpoints, such as RECIST (Response Evaluation Criteria in Solid Tumors), progression-free survival, and overall survival, and (v) clear evidence that Raman-based approaches provide added clinical value beyond existing imaging and histopathological methods.

In this context, the clinical translation of Raman-based approaches for therapy response monitoring is generally considered a long-term process that would take 8 to 12 years while moving through established technology readiness levels (TRLs), as shown in Fig. 15.^435^ This process can be divided into five main phases, as outlined by Santos *et al.* (2017) for the translation of Raman spectroscopy into cancer detection and surgical guidance.^409^ Preclinical discovery (years 1–3, TRL 1–3) establishes proof-of-concept by identifying therapy-resistance signatures in patient-derived models before and after treatment, followed by analytical validation (years 3–5, TRL 4–5) to ensure technical robustness and harmonization across instruments and laboratory environments. At this stage, two major bottlenecks emerge: biological heterogeneity between experimental models and human disease,^436^ and the growing regulatory requirements for clinical AI systems, particularly regarding explainability and transparency (Fig. 15, left panels).^437^ Clinical validation (years 5–8, TRL 6–7) then assesses predictive performance in prospective multicenter studies using standardized clinical endpoints such as RECIST. Regulatory approval (years 8–10, TRL 8) requires authorization of both the Raman platform and its associated algorithms (*e.g.*, FDA clearance or CE marking), while clinical adoption (years 10–12+, TRL 9) depends on successful integration into routine workflows and demonstration of clinical and economic value, particularly through reducing ineffective or toxic treatments.^409^ Achieving this final stage represents overcoming the so-called translational “valley of death”, the critical gap between basic research and clinical implementation that prevents many promising technologies from reaching patient care.^438^ Additionally, when AI or ML algorithms are incorporated, further regulatory requirements arise. Software as a medical device (SaMD) frameworks require algorithm transparency, version control, cybersecurity compliance, and clearly defined update strategies. Continuous-learning systems introduce additional challenges, as regulatory authorities demand controlled change management and re-validation after relevant modifications. However, the integration of ML or AI tools may ultimately facilitate the clinical adoption of Raman-based technologies by providing simplified and interpretable outputs, addressing the difficulties many clinicians face when processing large and complex datasets.

**Fig. 15:**
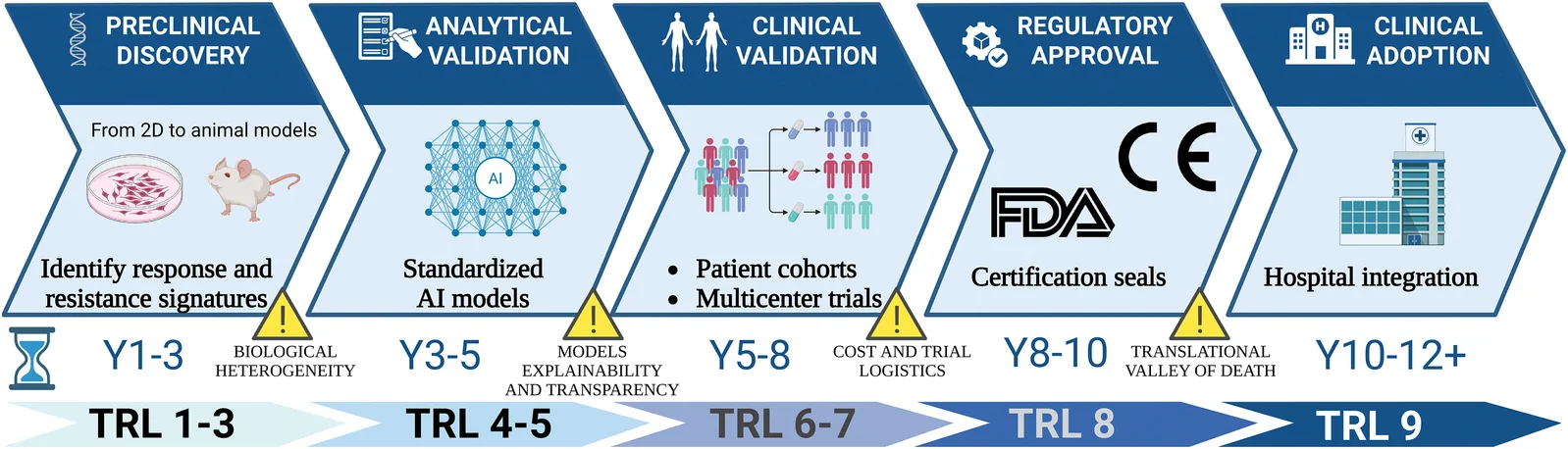
Roadmap for the clinical translation of Raman spectroscopy for therapy response monitoring. The pipeline illustrates the progression from preclinical discovery (TRL 1–3) to full clinical adoption (TRL 9) over a 12+ year timeline. Critical milestones include analytical AI validation, multicenter clinical trials, and regulatory approval, while explicitly highlighting major translational bottlenecks between phases, such as biological heterogeneity, AI explainability, trial logistics, and the translational valley of death.

Beyond regulatory approval, effective clinical translation of Raman-based technologies requires seamless integration into established healthcare workflows. In surgical environments, intraoperative Raman systems must avoid prolonging operative time, compromising sterile fields, or interfering with standard imaging modalities. Similarly, in pathology laboratories, Raman platforms should be compatible with existing specimen processing pipelines, digital pathology infrastructures, and reporting standards. Key determinants of adoption include rapid time-to-result, operational simplicity, and minimal training requirements. Importantly, regulatory clearance does not ensure clinical uptake. Broad implementation within national healthcare systems typically requires robust health technology assessment (HTA), including demonstration of cost-effectiveness, reduction of unnecessary treatments, or improved outcome prediction. Clinical adoption ultimately depends on clear added value in decision-making without disrupting routine practice.

The history of the Verisante Aura system exemplifies this translational gap. Verisante Aura is a Raman-based device using a 785 nm laser coupled to a fiber-optic probe for rapid (∼1 second) diagnosis of skin cancer, particularly melanoma.^407^ Clinical studies reported promising performance, leading to approval in Canada and CE marking in Europe in 2011. Despite regulatory authorization, the system did not achieve widespread clinical adoption, illustrating that approval alone is insufficient without clear clinical utility, workflow integration, and confidence among end users. Concerns were raised regarding limited sensitivity and the potential risk of missed melanoma diagnoses.^439^ To the best of our knowledge, the original Verisante Aura system is no longer in routine clinical use. This example highlights that successful translation of Raman-based therapy monitoring requires coordinated collaboration among engineers, clinicians, data scientists, regulatory experts, and health-policy stakeholders to ensure not only technical validation but true clinical integration. In parallel, INVENIO NIO laser imaging system provides a more encouraging example of Raman-based clinical translation: originating from stimulated Raman histology for intraoperative neurosurgical assessment, the platform obtained CE marking in 2021, is reported as FDA-registered in the United States, and has been used in more than 10 000 procedures, while its AI-based lung cancer reveal module recently received FDA breakthrough device designation.^13^

### 9.2 Clinical trials using Raman spectroscopy in therapy response monitoring and prediction

From a clinical perspective, a critical next step is the continuation of prospective trials demonstrating that Raman spectroscopy can predict therapeutic response. Solid tumors accessible at the body surface represent optimal initial models. For example, in head and neck cancer, Raman measurements could be performed (i) before neoadjuvant immunotherapy and after each treatment cycle, with response assessed by standard radiological imaging (MRI/CT), or (ii) prior to radiochemotherapy, with tumor response evaluated after six months by imaging or re-biopsy as the primary endpoint. In both scenarios, AI-based spectral analysis could be used to differentiate responders from non-responders. Given its non-destructive nature, Raman spectroscopy could be repeated longitudinally to determine whether temporal spectral changes enhance predictive accuracy. Implementation in multicenter trials will be essential, requiring harmonized instrumentation and strict standardization to ensure reproducible and comparable results across study sites.

As of July 2026, ClinicalTrials.gov listed 59 clinical trials involving Raman spectroscopy and SERS in the field of oncology. The most frequently evaluated malignancies include gastrointestinal (22 studies), hepatopancreatobiliary (18 studies), breast (13 studies), skin (11 studies), and lung cancers (11 studies). From this comprehensive dataset, a highly targeted subset of 24 trials explicitly evaluates therapeutic interventions, focusing their primary or secondary outcomes on treatment efficacy, patient response prediction, measurable residual disease (MRD) detection, or intraoperative margin assessment. Purely diagnostic or risk-stratification studies (35) aimed solely at differentiating benign from malignant lesions in untreated populations were strictly excluded from this subset. The clinical landscape represented by the selected 24 studies is currently dominated by surgery-guided tissue assessment (13 studies, 54.1%), indicating that intraoperative margin clearance is the most mature clinical application of Raman technologies. This is followed by systemic therapy monitoring *via* liquid biopsies (9 studies, 37.5%) and radiotherapy response evaluation (2 studies, 8.4%).

Intraoperative margin clearance utilizes Raman spectroscopy to directly influence surgical decision-making, with recent trials increasingly adopting multimodal optical platforms to overcome inherent field-of-view limitations. For example, in breast-conserving surgery, the Spectra-BREAST trial (NCT07111728) evaluates 104 patients by integrating hyperspectral imaging (400–1400 nm) to flag macroscopic suspicious regions, which are subsequently interrogated by point-wise Raman spectroscopy to acquire high-resolution molecular fingerprints and classify positive margins using artificial intelligence. Similarly, trial NCT06977698 utilizes an optical coherence tomography (OCT)-Raman system where OCT filters out normal adipose tissue based on reflectivity, directing Raman measurements exclusively to high-risk stromal regions to detect residual malignant cells. Other significant surgical applications include the 600-patient NCT03482622 trial testing an automated “Fast Raman” device to map chemical profiles of excised skin layers within 30 minutes during Mohs micrographic surgery, and the fluorescence enhanced stereotactic surgery (FESS) study (NCT07286903), which integrates 5-ALA fluorescence, Raman spectroscopy, and ultrasound into a biopsy needle to provide real-time feedback on high-grade glioma margins and microvessels.

In the area of systemic therapy, liquid biopsy-based Raman applications are rapidly advancing to track molecular changes and predict therapeutic outcomes. The most technologically complex example is NCT06772376 in advanced lung cancer, which combines catalytic hairpin assembly (CHA) mediated signal amplification with SERS for the direct detection of EGFR mutations (Del-19, T790M, L858R) in circulating tumor DNA, achieving over 95% sensitivity in preliminary phases to monitor targeted therapies. Another prominent trial, NCT05190094, aims to predict individual treatment efficacy for the combination of palbociclib and letrozole or anastrozole in advanced breast cancer using infrared laser spectroscopy and Raman benchmark analysis on serial liquid biopsies; this study employs deep data mining and support vector machines (SVM) with a polynomial kernel to differentiate responders from non-responders. Other studies focus on tissue biochemical fingerprints post-treatment, such as NCT04817449, which performs *ex vivo* Raman analysis on post-chemotherapy peritoneal or ovarian tissues to identify active cancer cells surviving within fibrotic tissue, and NCT06394050, which uses label-free Raman spectroscopy to discriminate between residual breast tumors and adjacent healthy tissues following neoadjuvant chemotherapy. Radiotherapy and MRD are also actively investigated; for instance, NCT06291428 directly compares Raman analysis of bone marrow aspirates against flow cytometry for MRD detection in acute lymphoblastic leukemia, while NCT00951535 predicts radiation response and toxicity in prostate cancer using pre- and post-treatment Raman spectra from lymphocytes and sera coupled with machine learning methodologies.

A critical methodological distinction across these 24 interventional trials is the reliance on native biomolecular signatures *versus* artificial contrast agents. The vast majority (21 studies) employ strictly label-free spontaneous Raman or label-free SERS, interrogating the intrinsic vibrational footprints of native lipids, proteins, and nucleic acids without functionalized tags. For example, trial NCT06087016 analyzes the urine of 1800 bladder cancer patients using bare silver nanoparticles to globally amplify the native metabolic profile rather than targeting a specific protein. Only a fraction utilizes explicitly targeted nanotags, such as the “sandwich” EpCAM-targeted SERS probes used in NCT07018505 to detect micro-metastases in sentinel lymph nodes (measuring peak areas within the 540–570 cm^−1^ Raman shift), or the engineered bacteria deployed in NCT06048835 to bind to circulating tumor cell membranes.

### 9.3 Future directions and innovations

The next phase of Raman spectroscopy in oncology should move beyond isolated proof-of-concept studies toward integrated workflows capable of supporting precision oncology. In this perspective, Raman-based approaches are unlikely to replace established imaging, pathology, or molecular assays, but may complement them by providing rapid, label-free, and spatially resolved biochemical information. Their strongest contribution may lie in settings where conventional methods provide limited molecular insight, such as early treatment assessment, residual disease detection, tumor heterogeneity mapping, and longitudinal monitoring of patient-derived models or samples obtained through minimally invasive procedures.

A major future direction will be the development of Raman-based deep phenotyping strategies. Rather than interpreting spectra only as collections of individual vibrational bands, future studies should connect Raman-derived chemical phenotypes with biological mechanisms, molecular pathways, and clinically relevant endpoints. Integration with histopathology, omics, imaging, and treatment-response data will be essential to link spectral fingerprints to mechanisms of sensitivity, resistance, metabolic adaptation, immune remodeling, and therapy-induced phenotypic changes.

Equally important will be the evolution toward intelligent multimodal platforms. Raman spectroscopy offers high molecular specificity, but its speed, sampling depth, and field of view remain limited compared with other imaging modalities. Future systems should therefore combine Raman readouts with faster wide-field or morphology-based techniques, using them to guide targeted Raman interrogation of biologically or clinically relevant regions. Artificial intelligence will be central in this transition, not only for classification, but also for region selection, quality control, spectral interpretation, and potentially adaptive acquisition strategies in which measurements are optimized in real time according to the information already collected.

Ultimately, successful innovation will depend on usability and clinical value. Raman-based tools will need to deliver robust, reproducible, and interpretable outputs that fit naturally into surgical, pathological, oncological, or laboratory workflows. This requires reducing acquisition and analysis time, minimizing operator dependency, ensuring inter-site reproducibility, and presenting results in a form that can support decision-making without requiring spectroscopy expertise. The future impact of Raman spectroscopy in cancer therapy monitoring will therefore depend not only on better instruments or algorithms, but on its ability to generate clinically validated and actionable information that adds clear value to existing standards of care.

## 10 General criteria

This review analyzed the state-of-the-art in label-free Raman spectroscopy for monitoring and predicting cancer therapy response. A rigorous search protocol was implemented across PubMed, Scopus, and Google Scholar, covering literature published between 2010 and 2026. The primary search string combined keywords such as “Raman spectroscopy,” “label-free,” “cancer,” “therapy response,” “monitoring,” and “prediction.” To establish a foundational technical guide for the new generation of researchers (Sections 3–5), the search scope was expanded to include critical technical papers on Raman data analysis and machine learning, and significant studies from the field of cancer diagnosis. This comprehensive approach ensures that the review not only covers the core clinical application but also provides a broad methodological framework suitable for various oncology research settings, illustrating the most utilized techniques across therapy assessment. Studies relying exclusively on exogenous Raman labels or tags for therapy response monitoring/prediction were excluded, whereas label-free SERS and approaches providing intrinsic biochemical information were considered.

## Author contributions

V. A.-R. and R. V. conceived the review. V. A.-R. performed the initial literature analysis, drafted the initial manuscript and created all original figures. V. A.-R., A. B., A. M., C. A., C. M., D. P., O. G.-L. and R. V. contributed to literature analysis, manuscript drafting, critical interpretation and revision. V. A.-R. and R. V. coordinated the preparation of the final manuscript. All authors reviewed and approved the final version of the manuscript.

## Conflicts of interest

There are no conflicts to declare.

## Supplementary Material

CS-055-D6CS00966B-s001

## Data Availability

No primary research results, software or code have been included and no new data were generated or analysed as part of this review. Supplementary information (SI) contains a comprehensive table summarising therapy-induced Raman spectral shifts, associated biomolecular mechanisms, and resistance phenotypes across preclinical and clinical cancer models. See DOI: https://doi.org/10.1039/d6cs00966b.
