# Supplementary material for: Label-free Raman spectroscopy for cancer therapy response: from methodological framework to clinical translation
Source: Chem Soc Rev. 2026 Oct 28;55(22):12122–89. doi: 10.1039/d6cs00966b (PMC13715935; doi:10.1039/d6cs00966b)
Supplement: Supplementary file 2 [file CS-055-D6CS00966B-s001.pdf]

Supplementary Information (SI) for Chemical Society Reviews.
This journal is © The Royal Society of Chemistry 2026
Supplementary Information (SI) for Chemical Society Reviews.
This journal is © The Royal Society of Chemistry

Label-free Raman Spectroscopy for Cancer Therapy Response: From Methodological Framework to Clinical Translation

Victor Alcolea-Rodriguez[1,2 *], Arianna Bresci[2,3], Anna Mühlig[4,5], Chiara Agrimi[1,2], Carlo Morasso[6], Dario Polli[1,2], Orlando Guntinas-Lichius[4,7], Renzo Vanna[1*]

[1]*CNR-Institute for Photonics and Nanotechnologies (IFN-CNR), P.zza Leonardo Da Vinci 32, 20133 Milan, Italy*

[2]*Department of Physics, Politecnico di Milano, P.zza Leonardo da Vinci 32, 20133 Milan, Italy*

[3]*Department of Mechanical Engineering, Massachusetts Institute of Technology, 77 Massachusetts Avenue, 02139 Cambridge MA, USA*
[4]*Clinical Biophotonics, Department of Otorhinolaryngology, Jena University Hospital, 07747 Jena, Germany.*

[5]*Comprehensive Cancer Center Central Germany, 07747 Jena, Germany.*

[6]*Laboratory of Nanomedicine, Istituti Clinici Scientifici Maugeri IRCCS, Pavia, Italy*

[7]*Department of Otorhinolaryngology, Jena University Hospital, 07747 Jena, Germany.*

*Correspondence email: victor.alcolea@polimi.it, renzo.vanna@cnr.it

Table S1. Comprehensive overview of therapy-induced Raman spectral shifts, biomolecular mechanisms, and resistance phenotypes across preclinical and clinical cancer models.

Increase/ decrease	***Raman shift (cm-1)***	***Biomolecule***	***Analysis type***	***Treatment***	***Drug/Dosis***	***Model (Cell/Tissue)***	***Cancer Type***	***Pathway/Mechanism***	***Sensitive or Resistant***	***Reference***
Increase	1003/1175/1208/1618	Aromatic Amino Acids (Phe/Tyr/Trp)	PCA	Radiotherapy	15-50 Gy (24-72h)	PC3/MDA-MB-231	Prostate/Breast	Protein Breakdown / ROS Scavenging	Resistant	Matthews et al. (2011)
Decrease	1600s	Beta-sheet Proteins	PCA	Radiotherapy	15-50 Gy	PC3/MDA-MB-231	Prostate/Breast	Protein Breakdown	Resistant	Matthews et al. (2011)
Increase	480/939/1388	Glycogen	PCA	Radiotherapy	2-10 Gy	H460/MCF7	Lung/Breast (p53 wt)	Metabolic Energy Reserve	Resistant	Harder et al. (2015)
Increase	717	Phospholipids	PCA	Radiotherapy	2-10 Gy	LNCaP	Prostate	Membrane Damage	Sensitive	Harder et al. (2015)
Increase	484/941/1378	Glycogen	PCA	Radiotherapy	0-10 Gy	H460/MCF7	Lung/Breast	Metabolic Adaptation	Resistant	Matthews et al. (2016)
Decrease	484/941	Glycogen	PCA	Metformin + Radiotherapy	Metformin	MCF7	Breast	Reversal of Resistance	Sensitive (Sensitized)	Matthews et al. (2016)
Increase	717	Phosphatidylcholine	GBR-NMF	Radiotherapy	Radiation	LNCaP	Prostate	Lipid Metabolism	Sensitive	Milligan et al. (2021)
Increase	484/1047/1127	Glycogen/Glucose	GBR-NMF	Radiotherapy	Radiation	H460/MCF7	Lung/Breast	Metabolic Biomarkers	Resistant	Milligan et al. (2021)
Increase	PC1 (Nucleic/AA)	Nucleic Acids/Amino Acids	PCA (Ex vivo)	Radiotherapy	15 Gy (Day 1)	MDA-MB-231 Xenograft	Breast	Transient S-phase Enrichment	Response	Van Nest et al. (2018)
Increase	Score	Glycogen/Alanine	GBR-NMF	Radiotherapy	15 Gy (Hypoxia)	H460 Xenograft	Lung	Hypoxia-induced Resistance	Resistant	Xinchen Deng et al. (2022)
Increase	484/1047/1340/1743	Glycogen/Lipids/AA	CNN/Grad-CAM	Radiotherapy	Radiation	H460/MCF7	Lung/Breast	Metabolic Reprogramming	Resistant	Fuentes et al. (2023)
Increase	1660/1245/1445	Proteins/Lipids	PCA	Radiotherapy	1.5-3 Gy	UVW	Glioma	Cell Cycle Modulation	Response	Hill et al. (2023)
Increase	Score	Lipids/Collagen	MCR-ALS	Radiotherapy	8 Gy	A549/UM-SCC-22B	Lung/H&N Xenograft	Matrix Remodeling	Sensitive	Paidi et al. (2019)
Increase	Score	Collagen	MCR-ALS	Radiotherapy	8 Gy	rA549/UM-SCC-47	Lung/H&N Xenograft (Resistant)	Extracellular Matrix Stiffness	Resistant	Paidi et al. (2019)
Increase	Signal	Lipids (Lipid-like)	In vivo Raman	Radiotherapy	2 Gy (48h)	UM-SCC-22B	Head & Neck Xenograft	Early Lipid Accumulation	Sensitive	Karunakaran et al. (2024)
Increase	Signal	Collagen	In vivo Raman	Radiotherapy	2 Gy (24h)	UM-SCC-47	Head & Neck Xenograft	Matrix Remodeling	Resistant	Karunakaran et al. (2024)
Decrease	669/728/782/830	DNA (Bases/Backbone)	PCA	Chemotherapy	Doxorubicin (IC50)	A549	Lung	DNA Intercalation / Apoptosis	Sensitive	Byrne et al. (2015)
Decrease	1303/1440	Lipids	PCA	Chemotherapy	Doxorubicin	A549	Lung	Membrane Integrity Loss	Sensitive	Byrne et al. (2015)
Increase	440/465/1215/1245	Drug Signal (DOX)	PCA	Chemotherapy	Doxorubicin	Calu-1	Lung	Nuclear Drug Accumulation	Response (Uptake)	Byrne et al. (2017)

Increase	1206	Drug Signal (DOX)	Confocal Raman	Chemotherapy	DNA-Nanodevice DOX	DU145	Prostate	Enzyme-Triggered Release	Sensitive	Tanwar et al. (2025)
Increase	Component	Lipids	MCR-ALS	Immunotherapy	Anti-CTLA4	CT26 Xenograft	Colorectal	Lipid Metabolism Alteration	Response	Paidi et al. (2021)
Decrease	Component	Lipids	MCR-ALS	Immunotherapy	Anti-PD-L1	CT26 Xenograft	Colorectal	Lipid Metabolism Alteration	Response	Paidi et al. (2021)
Decrease	654/668/783/ 1096	DNA (Bases/Backbon e)	Multivariat e	Targeted Therapy	Cetuximab	CC Cells	Colorectal (KRAS WT)	EGFR Inhibition	Sensitive	Cutshaw et al. (2024)
Increase	700	Cholesterol	Multivariat e	Targeted Therapy	Cetuximab	CC Cells	Colorectal (KRAS WT)	Membrane Stress	Sensitive	Cutshaw et al. (2024)
No Change	1096	DNA	Multivariat e	Targeted Therapy	Cetuximab	HCT116	Colorectal (KRAS G13D)	Lack of Response	Resistant	Cutshaw et al. (2024)
Decrease	2851	Lipids (CH2)	SRS Microscop y	Targeted Therapy	TOFA (Lipid Inhibitor)	LNCaP	Prostate	De Novo Lipogenesis Blockade	Sensitive	Hislop et al. (2022)
Decrease	3015	Unsaturated Lipids	SRS Microscop y	Targeted Therapy	TOFA (Lipid Inhibitor)	LNCaP/PC3	Prostate	Lipid Droplet Remodeling	Sensitive	Hislop et al. (2022)
Decrease	752/1545/165 1	Cytochrome C/Proteins	Spontaneo us Raman	Targeted Therapy	Tamoxifen	HepG2	Liver	Mitochondrial Stress	Response	Ali et al. (2019)
Decrease	3022	Unsaturated Lipids	Hyperspec tral SRS	Targeted Therapy	SCD1 Inhibitor	Melanoma (Mesenchymal)	Melanoma	Ferroptosis/Lipid Peroxidation	Sensitive	Du et al. (2020)
Increase	2851/3010	Lipids (Droplets)	SRS Phaser	Targeted Therapy	Atorvastatin	MCF-7	Breast (ER+)	Compensatory Lipid Accumulation	Resistant	Tipping et al. (2022)
Decrease	1546-1620	Amino Acids (Trp/Phe)	PC-LDA	Photodynamic Therapy	Chlorin photodithazine	Rat Breast Tumor	Breast (In vivo)	Protein Breakdown/Necrosis	Sensitive	Bhattacharjee et al. (2018)
Increase	874/1452	Collagen	PC-LDA	Photodynamic Therapy	Chlorin photodithazine	Rat Breast Tumor	Breast (In vivo)	Matrix Remodeling/Repair	Response	Bhattacharjee et al. (2018)
Increase	1665/1454	Proteins (Amide I)/Lipids	PCA-LDA	Ablation	Microwave Ablation	Lung Tissue	Lung	Thermal Coagulation	Response	Song et al. (2020)
Decrease	450	Glutathione	PCA	Chemotherapy	Neoadjuvant	Breast Patients	Breast (Clinical)	Oxidative Stress Marker	Sensitive	De la Torre-Gutiérrez et al. (2022)
Increase	1450/1750	Lipids	PLS-DA	Chemotherapy	Neoadjuvant (NACT)	Ovarian Patients	Ovarian (Biofluids)	Systemic Hyperlipidemia	Response	Giamougiannis et al. (2021)
Decrease	2852/2910 ratio	Lipid/Protein Ratio	High-wavenumb er Raman	Surgery (Intraoperative)	Resection Margins	OCSCC Patients	Oral Cavity	Tumor vs Healthy Tissue	Diagnostic	Aaboubout et al. (2023)
Increase	1276/1330	Drug Signal (6-MP)	SERS	Chemotherapy	6-Mercaptopurine	A549	Lung	Intracellular Metabolism	Response	Han et al. (2014)
Increase	2221	Drug Signal (Alkyne)	SRS	Targeted Therapy	Ponatinib	KCL-22	CML (Leukemia)	Lysosomal Sequestration	Response	Sepp et al. (2019)
